# Global burden associated with rare infectious diseases of poverty in 2021: findings from the Global Burden of Disease Study 2021

**DOI:** 10.1186/s40249-024-01249-6

**Published:** 2024-11-13

**Authors:** Yujia Bao, Yongxuan Li, Yibin Zhou, Ne Qiang, Tianyun Li, Yuzheng Zhang, Marc K. C. Chong, Shi Zhao, Xiaobei Deng, Xiaoxi Zhang, Lefei Han, Jinjun Ran

**Affiliations:** 1 School of Public Health, Shanghai Jiao Tong University School of Medicine, Shanghai, China; 2 Minhang District Center for Disease Control and Prevention, Shanghai, China; 3 School of Global Health, Chinese Center for Tropical Diseases Research, Shanghai Jiao Tong University School of Medicine, Shanghai, China; 4 China National Health Development Research Centre, Beijing, China; 5 Jockey Club School of Public Health and Primary Care, Chinese University of Hong Kong, Hong Kong, China; 6 School of Public Health, Tianjin Medical University, Tianjin, China

**Keywords:** Rare infectious diseases of poverty, Disability-adjusted life-years, Global burden of disease, Neglected tropical diseases, One Health

## Abstract

**Background:**

Rare infectious diseases of poverty (rIDPs) involve more than hundreds of tropical diseases, which dominantly affect people living in impoverished and marginalized regions and fail to be prioritized in the global health agenda. The neglect of rIDPs could impede the progress toward sustainable development. This study aimed to estimate the disease burden of rIDPs in 2021, which would be pivotal for setting intervention priorities and mobilizing resources globally.

**Methods:**

Leveraging data from the Global Burden of Disease Study 2021, the study reported both numbers and age-standardized rates of prevalence, mortality, disability-adjusted life-years (DALYs), years lived with disability, and years of life lost of rIDPs with corresponding 95% uncertainty intervals (UIs) at global, regional, and national levels. The temporal trends between 1990 and 2021 were assessed by the joinpoint regression analysis. A Bayesian age-period-cohort model was used to project the disease burden for 2050.

**Results:**

In 2021, there were 103.76 million (95% UI: 102.13, 105.44 million) global population suffered from rIDPs with an age-standardized DALY rate of 58.44 per 100,000 population (95% UI: 42.92, 77.26 per 100,000 population). From 1990 to 2021, the age-standardized DALY rates showed an average annual percentage change of − 0.16% (95% confidence interval: − 0.22, − 0.11%). Higher age-standardized DALY rates were dominated in sub-Saharan Africa (126.35 per 100,000 population, 95% UI: 91.04, 161.73 per 100,000 population), South Asia (80.80 per 100,000 population, 95% UI: 57.31, 114.10 per 100,000 population), and countries with a low socio-demographic index. There was age heterogeneity in the DALY rates of rIDPs, with the population aged under 15 years being the most predominant. Females aged 15–49 years had four-times higher age-standardized DALY rates of rIDPs than males in the same age. The projections indicated a slight reduction in the disease burden of rIDPs by 2050.

**Conclusions:**

There has been a slight reduction in the disease burden of rIDPs over the past three decades. Given that rIDPs mainly affect populations in impoverished regions, targeted health strategies and resource allocation are in great demand for these populations to further control rIDPs and end poverty in all its forms everywhere.

**Graphical Abstract:**

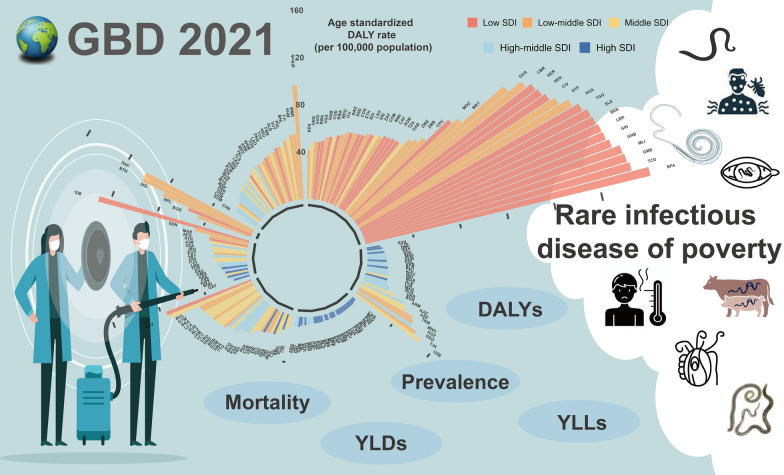

**Supplementary Information:**

The online version contains supplementary material available at 10.1186/s40249-024-01249-6.

## Background

Infectious diseases of poverty (IDPs) involve a diverse group of conditions of parasitic, bacterial, viral, and non-communicable origin, such as neglected tropical diseases (NTDs), malaria, tuberculosis, and human immunodeficiency virus/acquired immunodeficiency syndrome, which disproportionately affected poor and marginalized populations [1, 2]. The World Health Organization (WHO) and other international communities have played active roles in promoting control, elimination, or eradication of IDPs. However, hundreds of IDPs have not been prioritized due to their sporadic incidence or the lack of effective diagnostic tools, and they are more prevalent among poorer populations, causing devastating impacts on impoverished communities [3]. The group of diseases is called rare infectious diseases of poverty (rIDPs), which represents an overarching concept to put the emphasis on the poor and vulnerable individuals who have weak intervention capacity. Most rIDPs belong to NTDs, including relapsing fevers, typhus fever, epidemic myalgia, Ross River disease, taeniasis, trichinellosis, enterobiasis, congenital toxoplasmosis, and others. People prevalent with rIDPs also endure disability, stigmatization, debilitation, and poverty-promoting chronic conditions or preventable deaths [4, 5].

Globally, rIDPs predominantly occur in low- and middle-income regions, such as Africa, South Asia, Latin America, and the Caribbean, and are the root causes of poverty and hunger in these regions [5]. Evidence suggests the disease burden of rIDPs could account for more than a quarter of major NTDs, despite a sparse prevalence of any single type of rIDPs [3]. Benefited from a series of global interventions and five core strategies raised by the WHO, including preventive chemotherapy, individual disease management, vector control, veterinary public health, as well as water, sanitation, and hygiene, there has been a significant progression in the control, elimination, or eradiation of rIDPs or NTDs over the past decades [6, 7]. The Global Burden of Disease Study (GBD) 2019 reported a decreasing burden of 15 specific major NTDs with an aggregated age-standardized disability-adjusted life-year (DALY) rate from 463.4 per 100,000 population in 1990 to 170.9 per 100,000 population in 2019 [5]. However, due to the diverse underlying causes, various associated outcomes, and lack of diagnostic tools and effective surveillance systems, it lacks timely estimation of the disease burden of rIDPs. Meanwhile, the changed external environments, such as the persistent recession of major global economies, global climate crisis, increasing social unrest in countries and regions, poor hygiene and sanitation, inadequate public health and health system infrastructure, and low levels of education, might lead to parts of rIDPs turn into a new crisis if there are not enough resources and attention supporting the established elimination strategies [8–11]. In recent, several diseases seem to be resurging even in developed regions [12, 13].

Currently, rIDPs remain absent from the priorities of the global health agenda with limited resources and global funding for disease control and prevention. The extreme imbalance in resource allocation will exacerbate health inequities, further entrenching poverty in all its forms and hindering efforts to eradicate it globally. This imbalance will also impede the promotion of good health and well-being, posing significant challenges to achieving the first and third Sustainable Development Goals (SDGs) by 2030 [14]. On the other hand, resources and attention will be hard to accumulate or allocate if lacking studies on estimating and monitoring the temporal-spatial distribution and disease burden of rIDPs, while information about the burden of rIDPs is quite limited.

Leveraging the GBD study 2021, this study reports the most updated disease burden estimates of rIDPs at global, regional, and national levels by age, sex, and social-demographic index (SDI). The secular trend (1990–2021), a projected disease burden for 2050, and a summarized road map for rIDPs interventions are also presented [15]. The results will provide a timely and systematic evaluation of the effectiveness of current rIDPs interventions across various countries and territories over the past three decades, and greatly contribute to the subsequent promotion of a global surveillance network and comprehensive elimination strategies targeted for rIDPs.

## Methods

### Data source

The global burden of rIDPs was obtained from the GBD database via the Global Health Data Exchange query tool (http://ghdx.healthdata.org/gbd-results-tool), which is a large health-related database maintained by the Institute for Health Metrics and Evaluation. The GBD 2021 encompassed the annual estimates of morbidity and mortality for 371 diseases and injuries, obtained from 204 countries and territories during 1990–2021. The detailed methodology for GBD 2021 is available elsewhere [16, 17]. As described in previous studies, a Bayesian meta-regression tool (DisMod-MR 2.1) was utilized to model and derive estimates of the disease burden metrics of rIDPs. The model incorporated all available data for rIDPs and corrected for known biases to derive specific estimates of the prevalence and burden. This study retrieved the following annual information from 1990 to 2021 for concern: GBD Estimate (cases of death or injury), measure [prevalence, deaths, DALYs, years lived with disability (YLDs), and years of life lost (YLLs)], metric (rate, number), cause (other neglected tropical diseases), location (global, SDI regions, GBD super regions, GBD sub-regions, and 204 countries and territories), age (all ages, age-standardized, age groups from < 5 years to 95+ years at a 5-year interval), sex (both, female, male), year (between 1990 and 2021). Countries and territories were divided into five groups based on SDI values as follows: low, low-middle, middle, high-middle, and high SDI quintiles (Additional file 1: Table S1). SDI is a standardized composite measurement index that ranges from 0 to 1 and is comparable across geographical locations and over time. It was calculated according to the national per capita income, the average educational years of individuals over 15 years, and the general birth rate [16, 17]. In addition, GBD classified regions into seven super regions and 21 sub-regions based on the epidemiologic similarity and geographical proximity. The details of region classification are provided in Additional file 1: Table S2. Population data from 1990 to 2050 was retrieved from the 2024 Revision of World Population Prospects (WPP) for each country and territory [18].

### Definition

The rIDPs include a diverse group of diseases caused by parasites, bacteria, viruses, and non-communicable diseases. Due to the sparse nature of epidemiological data for single-type rIDPs, the disease burden metrics of rIDPs in GBD 2021 were the aggregated values of other NTDs encompassing the following subtypes identified by the International Classification of Diseases (ICD-10) codes, including relapsing fevers (A68–A68.9), Lyme disease (A69.2–A69.29), other specified spirochaetal infections (A69.8–A69.9), typhus fever (A75–A75.9), spotted fever (A77–A77.9), Q fever (A79), other rickettsioses (A79–A79.9), other mosquito-borne viral fevers (A92–A92.9), oropouche virus disease (A93–A93.8), unspecified arthropod-borne viral fever (A94–A94.0), arenaviral hemorrhagic fever (A96–A96.9), other viral hemorrhagic fevers (A98–A98.8), other viral diseases (B33.0–B33.1), other protozoal diseases (B60–B60.8), unspecified protozoal disease (B64), Echinococcus multilocularis infection (B67.5–B67.7), teniasis (B68–B68.9), diphyllobothriasis, sparganosis, and other cestode infections (B70–B71.9), filariasis and trichinellosis (B74.3–B75), strongyloidiasis (B78–B78.9), enterobiasis, unspecified intestinal parasitism, and other intestinal helminthiases (B80–B83.8), unspecified parasitic disease (B89), congenital toxoplasmosis (P37.1). The detailed ICD-10 code of each disease is shown in Additional file 1: Table S3.

### Data analysis

The study retrieved the disease burden metrics with 95% uncertainty intervals (UIs) including the absolute numbers and age-standardized rates (ASR, per 100,000 population) of prevalence, mortality, DALY, YLD, and YLL presented as age-standardized prevalence rate (ASPR), age-standardized mortality rate (ASMR), age-standardized DALY rate, age-standardized YLD rate, and age-standardized YLL rate. Briefly, the ASR was computed by adding the age-specific rate and the number of persons belonging to the same age subgroup of the standard population, and then dividing by the sum of the standard population weights. The ASR was calculated as follows:

$$\text{ASR}=\frac{\sum_{i=1}^{N}{a}_{i}{w}_{i}}{\sum_{i=1}^{A}{w}_{i}}$$

where *a*_*i*_ is the age-specific rate for the *i*-th age group, *w*_*i*_ is the number of individuals in the corresponding age group in the GBD standard population, and *N* is the number of age groups. The 95% UI is defined as the 2.5th and 97.5th values of the ordered 1000 draws. The percentage change in each burden estimate between 1990 and 2021 was retrieved to compare the temporal change. The equation of percentage change is ${(values}_{1990}-{values}_{2021})/{values}_{1990}\times 100\%$. Meanwhile, a joinpoint regression analysis was also used to understand the temporal trends of rIDPs. The analysis used the grid search method (GSM) to identify years with notable changes in ASRs (joinpoints) during the entire period from 1990 to 2021 and segmented the entire period into multiple subperiods based on the observed joinpoints. The model selection was optimized by the Monte Carlo permutation test and the modified Bayesian Information Criterion with the minimized mean squared error to identify the preferred number of joinpoints and the lengths of each subperiod [19–21]. Then, the annual percentage change (APC) with a 95% confidence interval (*CI*) was calculated to represent the annual rate of change for each specified subperiod. The average annual percentage change (AAPC) with a 95% *CI* was also calculated as a weighted average of APCs based on the length of each segmented period to represent a summary measure of the trend over the entire period. The following log-transformed linear regression model was used for APC calculation:

$$\text{log}\left({R}_{y}\right)={b}_{0}+{b}_{i}y$$

where $\text{log}\left({R}_{y}\right)$ is the natural log of the ASR in the year $y.$ ${b}_{i}$ represents the slope coefficient for the *i*-th segmented subperiod, and ${b}_{0}$ represents the intercept. The APC from year $y$ to $y+1$ was calculated as follows:

$$APC= \left[\frac{{R}_{y+1-}{R}_{y}}{{R}_{y}}\right]=({e}^{{b}_{i}}-1)\times 100$$

When denoted ${w}_{i}$ as the length of each segmented subperiod, the AAPC across the entire period was calculated as follows:

$$AAPC=({e}^{\frac{\sum {w}_{i}{b}_{i}}{\sum {w}_{i}}}-1)\times 100$$

This study employed a maximum of five joinpoints to calculate APC and AAPC. The statistical significance was determined by two-sided *P* values < 0.05. A decreasing temporal change was identified if the upper boundary of the 95% UI or 95% *CI* of percentage change, APC, or AAPC was below zero. Conversely, an increasing temporal change was considered if the lower boundary of the 95% UI or *CI* was greater than zero. A non-significant change or a stable trend was considered if the 95% UI or 95% *CI* was across zero. The subgroup analyses were conducted with stratification by age group (20 groups at 5-year intervals), sex (female and male), SDI level (low SDI, low-middle SDI, middle SDI, high-middle SDI, and high SDI), super region (seven regions), 21 sub-region, and country (204 countries or territories). In addition, choropleth circle plots and overall trends of rIDPs were developed to show the geographical and temporal variation of the ASRs. The statistical significance between groups was considered if their UIs or *CI*s did not overlap.

A smoothing spline model with the Locally Weighted Scatterplot Smoothing (LOWESS) method was used to evaluate the relationship between the burden of rIDPs and SDI values for seven super regions and 204 countries and territories. The expected values were determined by a calculation considering the SDI values and disease rates across all locations. LOWESS allowed for automatically determining the degree, number, and location of nodes (knots) based on the data and the span parameter.

A Bayesian age-period-cohort (BAPC) model with default parameters was applied to project the ASPR, ASMR, age-standardized DALY, YLD, and YLL rates of rIDPs by multiple sex and location groups for the year 2050 [22, 23]. The BAPC model enables nonarbitrary and reasonable projections without strong parametric assumptions, which shows greater performance compared with other prediction models, such as the generalized additive model, smooth spline model, and Poisson regression [24]. The world population from the WPP between 1990 and 2050 was retrieved to calculate the predicted burden metrics. The BAPC model with integrated nested Laplace approximations examined the projected ASR ($\text{log}({\lambda }_{ij})$) and the multiplicative effects of age (${\alpha }_{i}$), period (${\beta }_{j}$), cohort (${\gamma }_{k}$), and general effect (intercept: $\mu$) with the following equation:

$$\text{log}({\lambda }_{ij})=\mu +{\alpha }_{i}+{\beta }_{j}+{\gamma }_{k}$$

The APCs and AAPCs were calculated by the Joinpoint Regression Program (Version 5.2.0, National Cancer Institute: Rockville, MD, United States). All the other analyses were conducted by the statistical software R 4.2.2 (Lucent Technologies, Jasmine Mountain, USA). The prediction results were calculated using BAPC and INLA packages in R and were presented by projected ASRs for the year 2050 with 95% *CI*s and estimated annual percentage changes (EAPC) in 2022–2050 with 95% *CI*s. The study was carried out in the public domain secondary database, without any nominal identity, hence, the ethical approval was exempted. The study conformed to the Precise and Transparent Health Estimates Reporting Guidelines [25].

## Results

### Global burden and temporal trends of rIDPs

In 2021, there were 103.76 million (95% UI: 102.13, 105.44 million) global population suffered from rIDPs, leading to 21.94 thousand (95% UI: 15.21, 26.89 thousand) associated deaths. The ASMR, age-standardized DALY rate, and age-standardized YLD rate of rIDPs were 0.30 per 100,000 population (95% UI: 0.21, 0.38 per 100,000 population), 58.44 per 100,000 population (95% UI: 42.92, 77.26 per 100,000 population), and 37.94 per 100,000 population (95% UI: 25.47, 54.88 per 100,000 population), respectively (Table 1 and Additional file 1: Table S4–S7). From 1990 to 2021, there were clear reductions in the global ASPR, age-standardized DALY rates, and age-standardized YLD rates, with AAPCs of − 0.28% (95% *CI:* − 0.32, − 0.25%), − 0.16% (95% *CI:* − 0.22, − 0.11%), and − 0.58% (95% *CI:* − 0.61, − 0.54%), respectively (Table 2 and Additional file 1: Tables S8–S12). In contrast, there were increased AAPCs in the global ASMR (0.53%, 95% *CI:* 0.46, 0.60%) and age-standardized YLL rate (AAPC: 0.77%, 95% *CI:* 0.67, 0.86%) during the same period (Additional file 1: Tables S8–S12).

**Table 1:** Age-standardized DALY rates and age-standardized YLD rates per 100,000 population for rIDPs and the percentage changes in the age-standardized rates, by sex, SDI levels, GBD super regions and sub-regions, from 1990 to 2021

Group	DALY^a^			YLD^b^		
	1990 (95% UI)	2021 (95% UI)	Change, %^c^ (95% UI)	1990 (95% UI)	2021 (95% UI)	Change, %^c^ (95% UI)
Global	61.60 (45.81, 81.26)	58.44 (42.92, 77.26)	− 5.14 (− 22.69, 8.34)	45.36 (30.89, 64.06)	37.94 (25.47, 54.88)	− 16.37 (− 19.58, − 12.90)
Female	71.41 (51.81, 96.22)	69.16 (50.16, 93.33)	− 3.15 (− 16.95, 7.10)	57.36 (39.17, 81.01)	52.02 (35.03, 74.72)	− 9.32 (− 12.88, − 5.82)
Male	52.12 (39.91, 67.93)	47.97 (34.02, 61.97)	− 7.96 (− 29.63, 12.04)	33.69 (22.78, 47.75)	24.24 (16.13, 35.25)	− 28.04 (− 32.96, − 22.36)
Low SDI^d^	135.83 (103.47, 178.98)	120.00 (88.43, 153.30)	− 11.65 (− 37.26, 7.07)	80.54 (54.60, 112.64)	63.8 (42.77, 91.37)	− 20.79 (− 26.03, − 16.07)
Low-middle SDI	97.83 (72.93, 131.58)	78.76 (57.50, 107.18)	− 19.49 (− 30.35, − 10.53)	79.77 (54.37, 112.73)	58.56 (39.11, 83.95)	− 26.59 (− 30.83, − 22.33)
Middle SDI	51.33 (38.63, 68.24)	36.91 (26.72, 50.63)	− 28.10 (− 35.87, − 21.47)	39.46 (26.80, 56.26)	28.38 (18.93, 41.09)	− 28.09 (− 31.23, − 24.84)
High-middle SDI	29.32 (21.44, 40.43)	15.79 (11.19, 21.95)	− 46.14 (− 49.62, − 42.71)	24.21 (15.99, 35.30)	13.41 (8.81, 19.55)	− 44.62 (− 48.05, − 41.06)
High SDI	10.10 (7.10, 14.47)	7.40 (5.31, 10.44)	− 26.68 (− 31.35, − 21.47)	8.65 (5.62, 13.00)	5.98 (3.92, 8.98)	− 30.89 (− 35.60, − 25.49)
Central Europe, Eastern Europe, and Central Asia	38.02 (26.87, 53.67)	29.75 (20.99, 42.76)	− 21.75 (− 27.13, − 16.30)	34.33 (23.04, 49.94)	26.81 (18.05, 39.64)	− 21.90 (− 27.47, − 15.86)
Central Asia	67.34 (46.33, 96.93)	53.44 (37.89, 75.80)	− 20.65 (− 27.83, − 12.09)	63.70 (42.89, 92.97)	48.76 (33.12, 71.08)	− 23.46 (− 30.92, − 15.19)
Central Europe	30.57 (20.58, 43.83)	16.47 (11.01, 24.00)	− 46.13 (− 50.90, − 41.36)	28.54 (18.34, 41.70)	15.74 (10.20, 23.31)	− 44.83 (− 49.88, − 39.19)
Eastern Europe	30.30 (21.60, 43.04)	20.88 (14.93, 29.34)	− 31.11 (− 39.50, − 23.44)	25.58 (16.81, 38.2)	17.78 (11.88, 26.27)	− 30.48 (− 40.30, − 21.69)
High-income	7.30 (5.10, 10.54)	5.91 (4.27, 8.30)	− 18.99 (− 26.00, − 11.06)	5.91 (3.72, 9.18)	4.48 (2.80, 6.88)	− 24.16 (− 32.09, − 15.43)
Australasia	5.81 (3.71, 8.98)	4.18 (2.60, 6.84)	− 28.15 (− 47.93, 1.31)	5.00 (2.93, 8.17)	3.80 (2.23, 6.47)	− 23.98 (− 47.21, 10.33)
High-income Asia Pacific	5.08 (3.67, 7.23)	6.64 (5.10, 8.78)	30.75 (12.81, 49.86)	3.76 (2.35, 5.87)	4.07 (2.51, 6.25)	8.27 (− 10.70, 30.48)
High-income North America	6.53 (4.20, 10.27)	4.49 (2.90, 6.96)	− 31.26 (− 44.36, − 15.76)	5.44 (3.10, 9.19)	3.75 (2.14, 6.17)	− 31.12 (− 45.57, − 12.50)
Southern Latin America	11.39 (7.05, 18.71)	8.10 (5.30, 12.80)	− 28.84 (− 52.71, 6.43)	9.38 (5.07, 16.73)	6.03 (3.25, 10.67)	− 35.78 (− 63.03, 5.85)
Western Europe	8.91 (6.23, 12.59)	5.56 (3.85, 8.09)	− 37.56 (− 44.2, − 30.28)	7.43 (4.78, 11.09)	4.92 (3.17, 7.42)	− 33.78 (− 41.49, − 24.89)
Latin America and Caribbean	64.56 (53.50, 78.98)	33.40 (24.93, 44.20)	− 48.27 (− 54.94, − 42.12)	34.02 (22.55, 48.53)	24.24 (15.82, 35.16)	− 28.75 (− 36.14, − 21.28)
Andean Latin America	54.27 (40.35, 72.57)	25.77 (18.26, 36.03)	− 52.51 (− 59.28, − 43.76)	36.67 (23.39, 53.10)	19.65 (12.72, 29.15)	− 46.41 (− 55.68, − 37.04)
Caribbean	51.65 (35.03, 72.36)	47.60 (31.79, 67.13)	− 7.85 (− 20.05, 5.41)	47.08 (30.33, 67.91)	43.24 (27.61, 62.90)	− 8.17 (− 21.77, 6.50)
Central Latin America	64.05 (56.76, 73.85)	22.39 (17.13, 29.41)	− 65.04 (− 70.76, − 59.09)	18.67 (12.38, 27.28)	13.34 (8.66, 19.79)	− 28.55 (− 33.16, − 23.45)
Tropical Latin America	69.75 (53.81, 90.11)	45.66 (33.32, 61.63)	− 34.54 (− 44.84, − 23.36)	47.26 (31.68, 68.19)	34.43 (22.27, 50.38)	− 27.14 (− 40.03, − 12.84)
North Africa and Middle East	48.62 (33.87, 66.95)	35.21 (25.04, 49.33)	− 27.57 (− 32.63, − 23.04)	43.92 (28.76, 62.45)	31.85 (21.43, 46.09)	− 27.48 (− 31.55, − 23.65)
North Africa and Middle East	48.62 (33.87, 66.95)	35.21 (25.04, 49.33)	− 27.57 (− 32.63, − 23.04)	43.92 (28.76, 62.45)	31.85 (21.43, 46.09)	− 27.48 (− 31.55, − 23.65)
South Asia	106.84 (75.74, 147.56)	80.80 (57.31, 114.10)	− 24.37 (− 29.36, − 18.89)	99.96 (68.47, 141.00)	73.70 (49.48, 106.76)	− 26.27 (− 30.62, − 21.56)
South Asia	106.84 (75.74, 147.56)	80.80 (57.31, 114.10)	− 24.37 (− 29.36, − 18.89)	99.96 (68.47, 141.00)	73.70 (49.48, 106.76)	− 26.27 (− 30.62, − 21.56)
Southeast Asia, East Asia, and Oceania	34.77 (25.51, 47.24)	20.57 (15.07, 28.05)	− 40.85 (− 46.33, − 36.19)	27.51 (18.32, 39.50)	15.90 (10.49, 23.24)	− 42.20 (− 45.07, − 38.66)
East Asia	28.88 (21.17, 38.94)	11.34 (8.10, 15.62)	− 60.73 (− 63.75, − 57.68)	23.01 (15.36, 33.07)	9.11 (5.91, 13.21)	− 60.41 (− 62.56, − 58.36)
Oceania	46.58 (31.01, 65.64)	42.86 (28.80, 65.23)	− 7.99 (− 26.88, 12.82)	38.55 (23.75, 57.02)	35.81 (21.90, 58.10)	− 7.11 (− 29.01, 17.84)
Southeast Asia	48.51 (34.74, 66.63)	35.82 (26.04, 48.54)	− 26.17 (− 35.96, − 17.76)	37.64 (24.61, 54.07)	26.70 (17.58, 38.72)	− 29.05 (− 35.02, − 22.31)
Sub-Saharan Africa	138.66 (106.83, 181.43)	126.35 (91.04, 161.73)	− 8.88 (− 40.12, 14.41)	60.64 (41.18, 85.52)	51.48 (34.06, 74.01)	− 15.11 (− 21.21, − 8.99)
Central sub-Saharan Africa	78.28 (51.82, 105.53)	54.44 (34.81, 76.23)	− 30.45 (− 47.54, − 11.36)	53.07 (35.25, 77.85)	33.30 (21.46, 49.73)	− 37.25 (− 52.17, − 21.17)
Eastern sub-Saharan Africa	87.50 (60.17, 117.30)	65.64 (44.02, 91.24)	− 24.98 (− 41.23, − 13.03)	61.15 (41.53, 85.50)	43.66 (29.40, 63.64)	− 28.60 (− 34.91, − 21.70)
Southern sub-Saharan Africa	73.67 (55.30, 98.54)	69.53 (50.27, 90.83)	− 5.62 (− 16.56, 6.92)	56.88 (37.93, 80.97)	51.21 (34.65, 71.68)	− 9.98 (− 20.17, 1.98)
Western sub-Saharan Africa	222.21 (172.62, 306.19)	203.12 (141.67, 260.86)	− 8.59 (− 45.32, 21.77)	63.66 (42.99, 92.40)	63.67 (41.46, 92.39)	0.02 (− 10.87, 12.35)

*DALYs* disability-adjusted life-years; *GBD* global burden of disease; *rIDPs* rare infectious diseases of poverty; *SDI* socio-demographic index; *UI* uncertainty interval; *YLD* years lived with disability

^a^Age-standardized DALY rates per 100,000 populations; ^b^ Age-standardized YLD rates per 100,000 population; ^c^ Percentage change in age-standardized rates between 1990 and 2021; ^d^ SDI is an indicator of a country’s development level and is comprised of the lag-dependent income per capita, the gross domestic product per capita smoothed over the previous 10 years, education level among the population aged ≥ 15 years old and the total fertility rate < 25 years old

**Table 2:** Temporal trends in age-standardized DALY rates of rIDPs by sex and SDI levels, from 1990 to 2021

Groups	Time periods identified		Temporal trend^a^					
			APC			AAPC		
			%	95% *CI*	*P* value	%	95% *CI*	*P* value
Global	Period1	1990–1997	− 0.29	(− 0.29, − 0.18)	< 0.001	− 0.16	(− 0.22, − 0.11)	< 0.001
	Period2	1997–2002	0.09	(0.09, 0.37)	0.003			
	Period3	2002–2006	− 0.20	(− 0.20, 0.23)	0.899			
	Period4	2006–2009	− 1.13	(− 1.13, − 0.31)	0.002			
	Period5	2009–2015	0.05	(0.05, 0.24)	0.004			
	Period6	2015–2021	− 0.62	(− 0.62, − 0.48)	< 0.001			
Female	Period1	1990–1993	− 0.50	(− 0.50, − 0.20)	0.001	− 0.10	(− 0.14, − 0.07)	< 0.001
	Period2	1993–1998	− 0.17	(− 0.17, 0.02)	0.117			
	Period3	1998–2006	0.14	(0.14, 0.22)	< 0.001			
	Period4	2006–2009	− 0.82	(− 0.82, − 0.23)	0.002			
	Period5	2009–2016	0.10	(0.10, 0.20)	0.000			
	Period6	2016–2021	− 0.60	(− 0.60, − 0.47)	< 0.001			
Male	Period1	1990–1997	− 0.36	(− 0.36, − 0.21)	0.001	− 0.26	(− 0.34, − 0.19)	< 0.001
	Period2	1997–2002	0.06	(0.06, 0.43)	0.013			
	Period3	2002–2006	− 0.42	(− 0.42, 0.13)	0.288			
	Period4	2006–2009	− 1.52	(− 1.52, − 0.45)	0.001			
	Period5	2009–2015	− 0.06	(− 0.06, 0.20)	0.251			
	Period6	2015–2021	− 0.80	(− 0.80, − 0.60)	< 0.001			
Low SDI	Period1	1990–1999	− 0.70	(− 0.70, − 0.61)	< 0.001	− 0.40	(− 0.47, − 0.33)	< 0.001
	Period2	1999–2006	− 0.28	(− 0.28, − 0.11)	< 0.001			
	Period3	2006–2011	− 0.93	(− 0.93, − 0.64)	< 0.001			
	Period4	2011–2014	0.43	(0.43, 1.33)	0.001			
	Period5	2014–2017	− 0.35	(− 0.35, 0.55)	0.650			
	Period6	2017–2021	− 1.17	(− 1.17, − 0.85)	< 0.001			
Low− middle SDI	Period1	1990–1993	− 0.87	(− 0.87, − 0.67)	< 0.001	− 0.70	(− 0.73, − 0.67)	< 0.001
	Period2	1993–2006	− 0.51	(− 0.51, − 0.48)	< 0.001			
	Period3	2006–2009	− 0.94	(− 0.94, − 0.55)	< 0.001			
	Period4	2009–2016	− 0.56	(− 0.56, − 0.49)	< 0.001			
	Period5	2016–2019	− 1.47	(− 1.47, − 1.07)	< 0.001			
	Period6	2019–2021	− 1.78	(− 1.78, − 1.37)	< 0.001			
Middle SDI	Period1	1990–1998	− 1.25	(− 1.25, − 1.16)	< 0.001	− 1.06	(− 1.11, − 1.00)	< 0.001
	Period2	1998–2002	− 0.63	(− 0.63, − 0.18)	< 0.001			
	Period3	2002–2009	− 1.04	(− 1.04, − 0.89)	< 0.001			
	Period4	2009–2014	− 0.77	(− 0.77, − 0.48)	< 0.001			
	Period5	2014–2018	− 2.14	(− 2.14, − 1.67)	< 0.001			
	Period6	2018–2021	− 1.56	(− 1.56, − 1.08)	< 0.001			
High-middle SDI	Period1	1990–1998	− 1.56	(− 1.56, − 1.44)	< 0.001	− 1.99	(− 2.07, − 1.90)	< 0.001
	Period2	1998–2001	− 1.46	(− 1.46, − 0.30)	0.005			
	Period3	2001–2004	− 2.92	(− 2.92, − 1.79)	< 0.001			
	Period4	2004–2010	− 3.40	(− 3.40, − 3.14)	< 0.001			
	Period5	2010–2018	− 2.26	(− 2.26, − 2.10)	< 0.001			
	Period6	2018–2021	− 1.19	(− 1.19, − 0.58)	< 0.001			
High SDI	Period1	1990–1993	− 2.82	(− 2.82, − 2.17)	< 0.001	− 0.99	(− 1.07, − 0.90)	< 0.001
	Period2	1993–1999	− 2.05	(− 2.05, − 1.75)	< 0.001			
	Period3	1999–2003	− 0.75	(− 0.75, − 0.11)	0.011			
	Period4	2003–2010	− 1.00	(− 1.00, − 0.78)	< 0.001			
	Period5	2010–2013	− 0.57	(− 0.57, 0.70)	0.836			
	Period6	2013–2021	− 0.54	(− 0.54, − 0.41)	< 0.001			

*APC* annual percentage change; *AAPC* average annual percentage change; *CI* confidence interval; *DALYs* disability-adjusted life-years; *GBD* global burden of disease; *rIDPs* rare infectious diseases of poverty; *SDI* social-demographic index

^a^Temporal trend in age-standardized DALY rates for rIDPs were analyzed by the Joinpoint Regression Program (Version 5.2.0, National Cancer Institute: Rockville, MD, United States)

### Regional burden and temporal trends of rIDPs

**Fig. 1:**
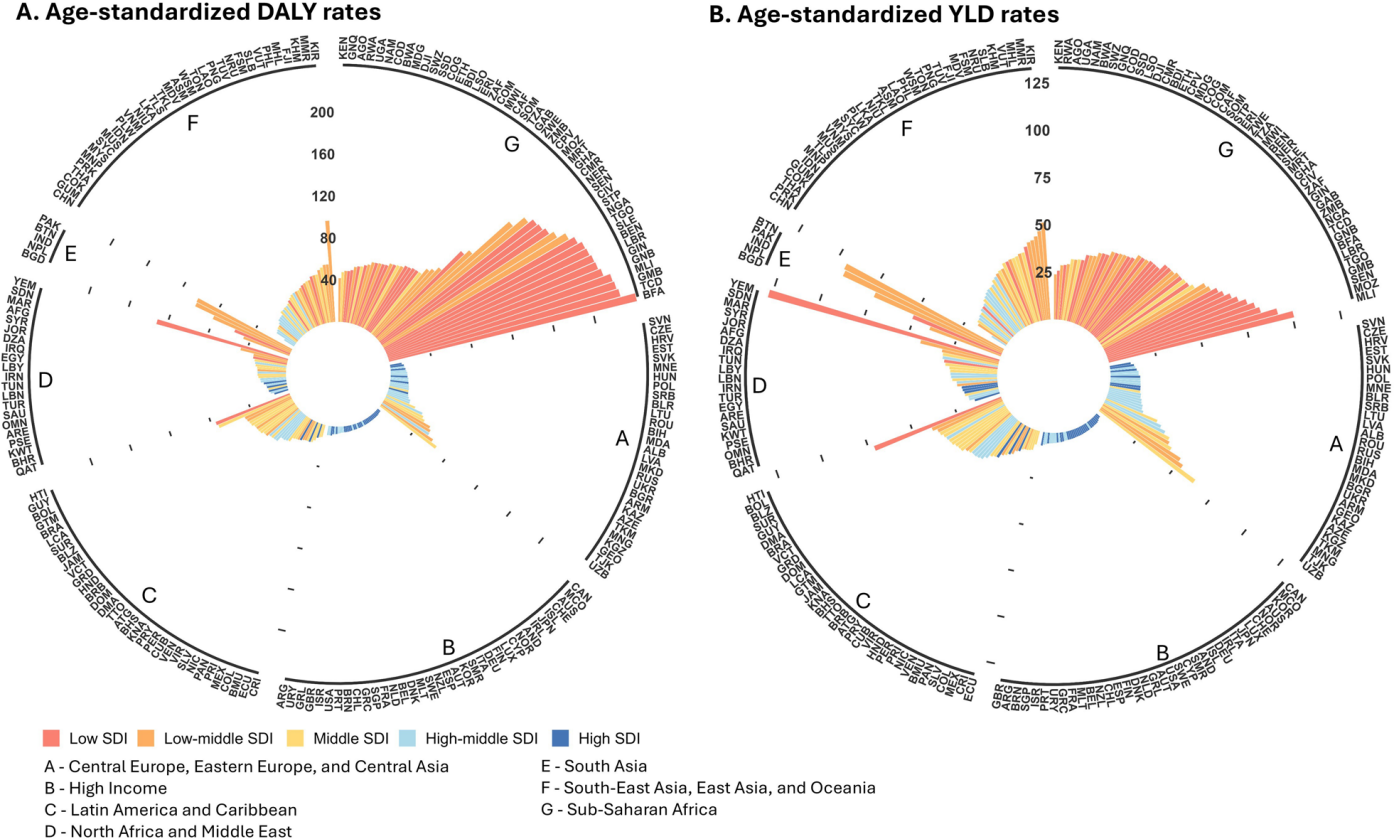
Age-standardized DALY and YLD rates (per 100,000 population) of rIDPs by countries and territories, in 2021. (**A** Age-standardized DALY rates of rIDPs. **B** Age-standardized YLD rates of rIDPs). The colors indicate the SDI levels of countries and territories. The black lines with capital letters indicate the GBD super regions for countries and territories. Countries and territories were abbreviated by ISO-3 code, and the full names can be found in Additional file 2. *DALY* disability-adjusted life-year; *YLD* year lived with disability; *rIDP* rare infectious disease of poverty

The geographic distribution showed that in 2021, the disease burden of rIDPs was dominated in sub-Saharan Africa and South Asia with age-standardized DALY rates of 126.35 per 100,000 population (95% UI: 91.04, 161.73 per 100,000 population) and 80.80 per 100,000 population (95% UI: 57.31, 114.10 per 100,000 population), respectively (Table 1). The two regions also showed less reductions in age-standardized DALY rates compared to other regions (AAPC in sub-Saharan Africa: − 0.30%, 95% *CI:* − 0.35, − 0.24% and South Asia: − 0.90%, 95% *CI:* − 0.92, − 0.87%) between 1990 and 2021. Among the most suffered countries and territories, Burkina Faso (243.16 per 100,000 population, 95% UI: 165.42, 337.39 per 100,000 population), Chad (229.10 per 100,000 population, 95% UI: 161.25, 336.69 per 100,000 population), and Gambia (227.39 per 100,000 population, 95% UI: 139.52, 327.70 per 100,000 population) accounted for the highest age-standardized DALY rates in 2021. For age-standardized YLD rates, Yemen (126.81 per 100,000 population, 95% UI: 87.86, 174.23 per 100,000 population), Mali (100.75 per 100,000 population, 95% UI: 66.18, 144.78 per 100,000 population), and Bhutan (94.47 per 100,000 population, 95% UI: 56.26, 140.72 per 100,000 population) accounted for the highest burden among countries and territories (Fig. 1). The global distributions of ASPR, ASMR, and age-standardized YLL rates are also shown in Additional file 1: Table S13. The AAPCs of 204 countries and territories between 1990 and 2021 can be found in Additional file 1: Table S14.

### Relationship between rIDPs burdens and SDI

**Fig. 2:**
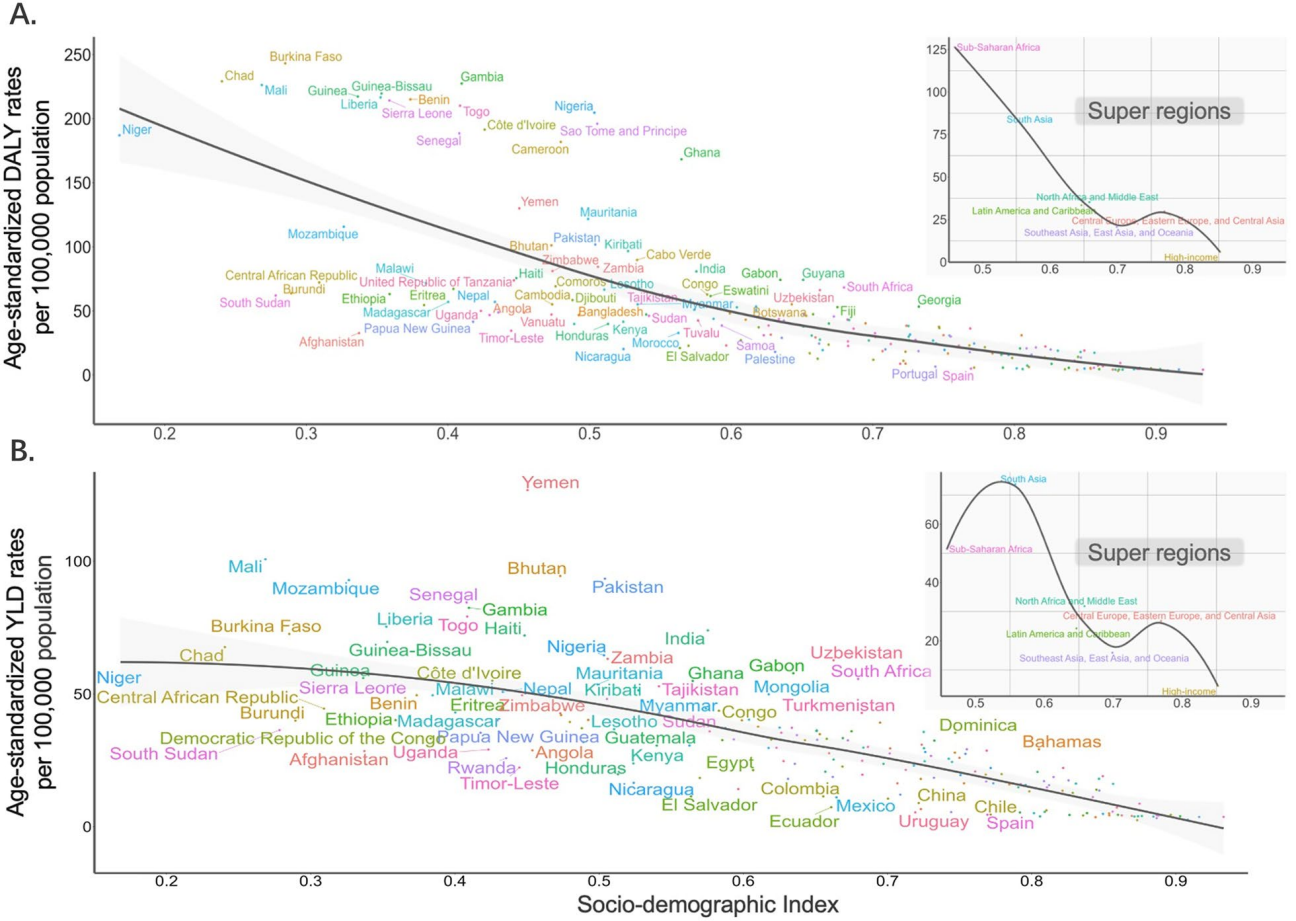
Associations of age-standardized DALY rates and age-standardized YLD rates of rIDPs (per 100,000 population) with SDI values by countries and territories and GBD super regions in 2021. (**A** Association between age-standardized DALY rates and SDI values. **B** Association between age-standardized YLD rates and SDI values.) The top right panels show the classification of GBD super regions. The expected values based on age-standardized rates and SDI values by a smoothing spline model with Locally Weighted Scatterplot Smoothing method are shown in the black lines. *DALY* disability-adjusted life-year; *GBD* global burden of disease; *rIDP* rare infectious disease of poverty; *SDI* socio-demographic index; *YLD* year lived with disability

**Fig. 3:**
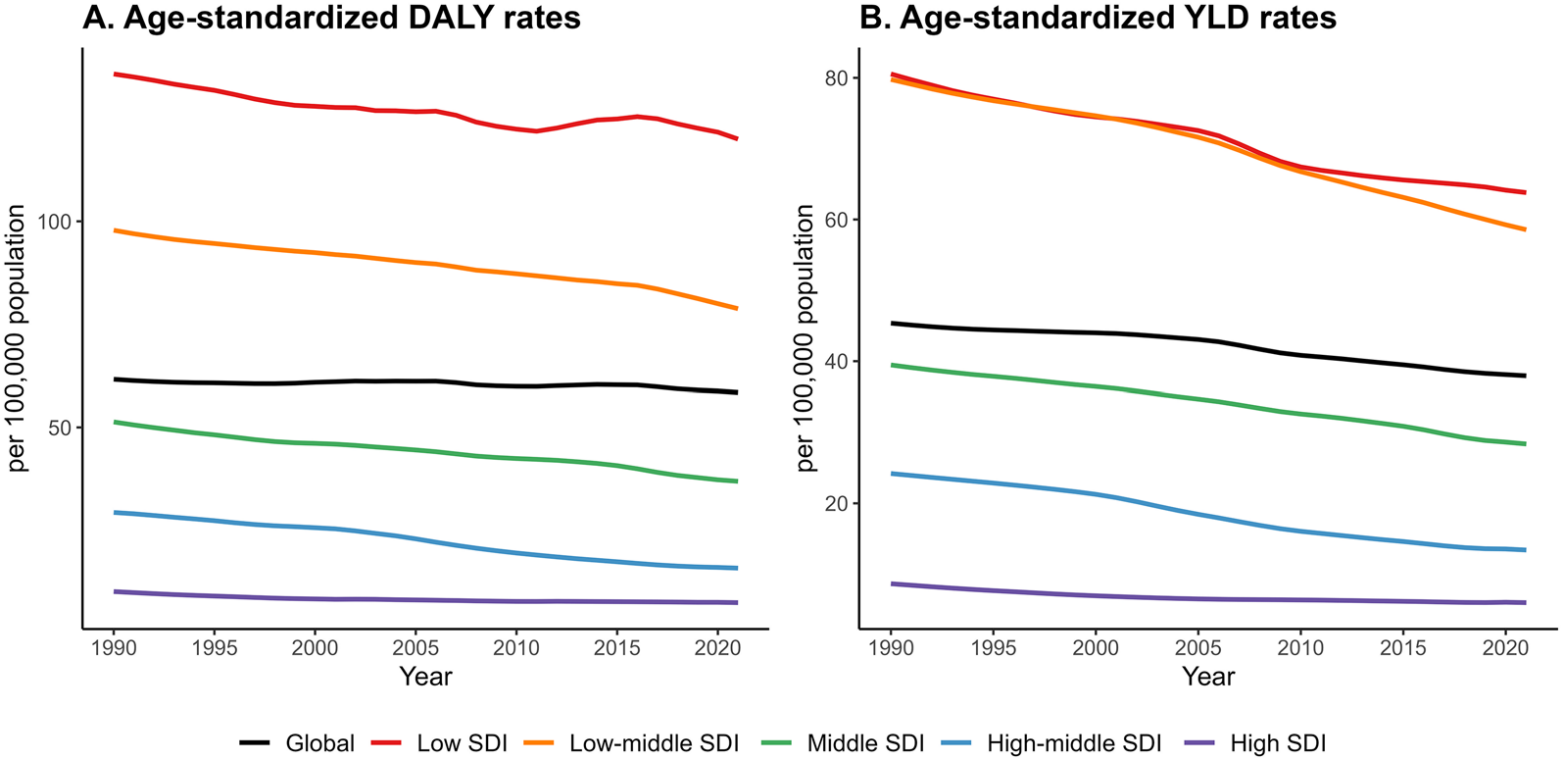
Temporal trends of age-standardized DALY rates and age-standardized YLD rates of rIDPs by SDI levels from 1990 to 2021. *DALY* disability-adjusted life-year; *rIDP* rare infectious disease of poverty; *SDI* socio-demographic index; *YLD* year lived with disability

For countries, territories, and regions with different SDI values, the ASPRs, ASMRs, age-standardized DALY, YLD, and YLL rates were negatively associated with SDI values (Fig. 2 and Additional file 1: Fig. S1–S3). In 2021, the age-standardized DALY rate in low SDI regions was 120.00 per 100,000 population (95% UI: 88.43, 153.30 per 100,000 population). In comparison, the value was 7.40 per 100,000 population (95% UI: 5.31, 10.44 per 100,000 population) in high SDI regions. There were also inverse relationships between temporal changes in the rIDPs burden metrics and SDI values. The temporal-spatial distributions of disease burden for rIDPs by SDI levels are shown in Fig. 3 and Additional file 1: Fig. S4. Low SDI regions also had the least reduction in AAPC of age-standardized DALY rate (− 0.40%, 95% *CI:* − 0.47, − 0.33%), compared to the largest AAPC reduction in high-middle SDI regions (− 1.99%, 95% *CI:* − 2.07, − 1.90%). Despite an overall downward trend of rIDPs burden globally, there have been sporadic resurgences in ASMR over the past three decades. For instance, a clear ASMR increase was observed in low SDI regions during 2011–2014 with an APC of 1.58% (95% *CI:* 0.74, 2.42%). Low-middle SDI regions showed an increasing ASMR trend during 2005–2010 (APC: 2.59%, 95% *CI:* 2.28, 2.89%). Even high SDI regions also showed a resurgence in ASMR of rIDPs in 2000–2003 (APC: 7.54%, 95% *CI:* 2.63, 12.69%) and 2010–2017 (APC: 1.85%, 95% *CI:* 1.22, 2.48%) (Additional file 1: Tables S8–S12).

### Age- and sex-specific patterns of rIDPs

**Fig. 4:**
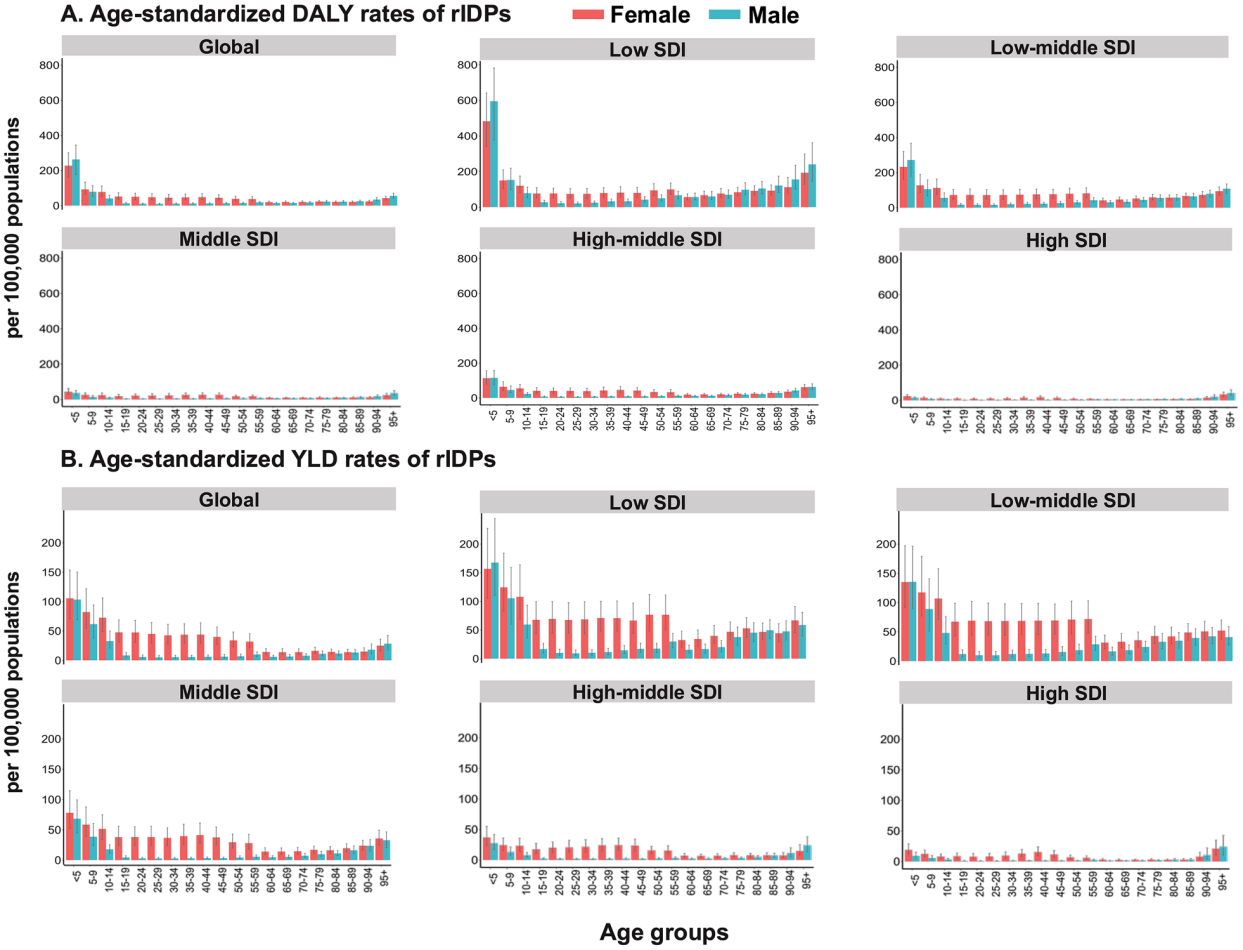
Age-standardized DALY rates and age-standardized YLD rates of rIDPs (per 100,000 population) by age groups, sex, and SDI levels in 2021. (**A** Age-standardized DALY rates of rIDPs. **B** Age-standardized YLD rates of rIDPs). In each panel, the horizontal coordinate represents age stratification at a 5-year interval, and the color of the columns represents sex stratification (females in red and males in blue). Error bars represent the corresponding 95% uncertainty intervals. *DALY* disability-adjusted life-year; *rIDP* rare infectious disease of poverty; *SDI* socio-demographic index; *YLD* year lived with disability

Age and sex heterogeneity in disease burden were observed for the age-standardized DALY rates associated with rIDPs, and the populations aged under 15 years and females were nearly predominated. In 2021, the age-standardized DALY rates in females were 227.35 per 100,000 population (95% UI: 162.72, 301.01 per 100,000 population) among people aged < 5 years, 92.43 per 100,000 population (95% UI: 64.19, 133.97 per 100,000 population) among those aged 5–9 years, and 77.90 per 100,000 population (95% UI: 54.01, 111.73 per 100,000 population) among those aged 10–14 years. In comparison, the values were 263.19 per 100,000 population (95% UI: 176.06, 344.74 per 100,000 population), 79.08 per 100,000 population (95% UI: 52.65, 113.90 per 100,000 population), and 40.76 per 100,000 population (95% UI: 28.12, 58.43 per 100,000 population) among the three serial age subgroups in males (Fig. 4). The sex disparity of age-standardized DALY rates was clear in the population aged 15–49 years, with estimates of 48.05 per 100,000 population (95% UI: 33.02, 67.19 per 100,000 population) in females and 11.95 per 100,000 population (95% UI: 8.81, 15.78 per 100,000 population) in males. Similarly, the sex disparity in age-standardized YLD rates was observed in the same age group, with the value of 44.41 per 100,000 population (95% UI: 29.59, 63.54 per 100,000 population) in females and 6.15 per 100,000 population (95% UI: 4.02, 9.56 per 100,000 population) in males. During 1990–2021, males had a larger reduction in ASPR (AAPC: − 0.61%, 95% *CI:* − 0.65, − 0.57%) and age-standardized DALY rate (AAPC: − 0.26%, 95% *CI:* − 0.34, − 0.19%), compared with females (AAPC of ASPR: − 0.08%, 95% *CI:* − 0.10, − 0.06% and AAPC of age-standardized DALY rate: − 0.10%, 95% *CI:* − 0.14, − 0.07%). The age- and sex-specific patterns for age-standardized prevalence, mortality, and YLL rates of rIDPs are presented in Additional file 1: Fig. S5.

### Projecting disease burden

Projection results from the BAPC model suggest that the global burden of rIDPs would continue to decline mildly. By 2050, the projected ASPR of rIDPs would be 1205.17 per 100,000 population (95% *CI:* 682.39, 1727.95 per 100,000 population), and the age-standardized DALY rate would be 53.21 per 100,000 population (95% *CI:* 18.30, 88.13 per 100,000 population). Countries and territories with low SDI levels, particularly in Western sub-Saharan Africa, are projected to suffer the highest burden of rIDPs. Meanwhile, the ASPRs in Oceania, South Asia, and Western sub-Saharan Africa are projected to remain high (Additional file 1: Table S15). The projected disease burden by sex showed declined trends in the same direction, while females would still have higher ASPR, age-standardized DALY and YLD rates than males by 2050 (Table 3).

**Table 3:** Predicted age-standardized DALY and YLD rates (per 100,000 population) of rIDPs from 2022–2050, by sex, SDI levels, GBD super regions and sub-regions, based on the Bayesian age-period-cohort model

Groups	DALY		YLD	
	2050 ASR (95% *CI*)	2022–2050 EAPC^a^ (95% *CI*)	2050 ASR (95% *CI*)	2022–2050 EAPC^a^ (95% *CI*)
Global	53.21 (18.30, 88.13)	− 0.10 (− 0.10, − 0.09)	30.57 (13.66, 47.49)	− 0.53 (− 0.54, − 0.53)
Female	62.39 (30.01, 94.78)	− 0.20 (− 0.21, − 0.20)	45.11 (25.57, 64.65)	− 0.38 (− 0.39, − 0.38)
Male	41.77 (7.60, 88.19)	− 0.15 (− 0.16, − 0.14)	16.81 (3.21, 30.41)	− 0.88 (− 0.90, − 0.87)
Low SDI	108.61 (62.77, 154.45)	− 1.31 (− 1.32, − 1.31)	52.80 (32.47, 73.14)	− 1.66 (− 1.67, − 1.65)
Low-middle SDI	43.81 (26.20, 61.42)	− 0.82 (− 0.82, − 0.82)	28.52 (18.35, 38.69)	− 1.27 (− 1.27, − 1.27)
Middle SDI	34.27 (20.88, 47.65)	− 1.88 (− 1.90, − 1.86)	29.66 (17.40, 41.93)	− 1.50 (− 1.51, − 1.49)
High-middle SDI	6.22 (0.70, 11.73)	− 1.60 (− 1.60, − 1.59)	6.61 (0.67, 13.89)	− 0.85 (− 0.89, − 0.82)
High SDI	4.70 (3.11, 6.30)	− 1.36 (− 1.39, − 1.34)	3.56 (2.10, 5.03)	− 1.60 (− 1.63, − 1.58)
Central Europe, Eastern Europe, and Central Asia	21.97 (14.78, 29.17)	− 0.87 (− 0.88, − 0.87)	21.52 (14.62, 28.41)	− 0.59 (− 0.60, − 0.58)
Central Asia	40.28 (15.31, 65.26)	− 0.77 (− 0.77, − 0.77)	36.48 (13.07, 59.89)	− 0.79 (− 0.79, − 0.79)
Central Europe	8.07 (5.46, 10.68)	− 2.39 (− 2.41, − 2.38)	7.99 (5.69, 10.30)	− 2.28 (− 2.29, − 2.27)
Eastern Europe	10.26 (6.99, 13.54)	− 2.26 (− 2.29, − 2.23)	9.65 (6.71, 12.60)	− 1.97 (− 1.99, − 1.95)
High-income	4.32 (2.80, 5.84)	− 0.94 (− 0.96, − 0.92)	3.07 (1.72, 4.42)	− 1.17 (− 1.20, − 1.14)
Australasia	2.70 (1.61, 3.78)	− 1.32 (− 1.34, − 1.30)	2.58 (1.61, 3.56)	− 1.11 (− 1.13, − 1.10)
High-income Asia Pacific	3.02 (1.69, 4.35)	− 1.30 (− 1.31, − 1.28)	2.51 (1.48, 3.55)	− 1.35 (− 1.37, − 1.33)
High-income North America	7.61 (3.83, 11.39)	0.62 (0.62, 0.62)	3.44 (2.33, 4.56)	− 0.45 (− 0.49, − 0.40)
Southern Latin America	5.31 (2.55, 8.07)	− 1.19 (− 1.21, − 1.16)	4.01 (1.39, 6.64)	− 1.12 (− 1.14, − 1.10)
Western Europe	3.16 (1.99, 4.33)	− 1.74 (− 1.75, − 1.72)	3.05 (1.76, 4.34)	− 1.43 (− 1.44, − 1.41)
Latin America and Caribbean	13.07 (7.53, 18.61)	− 3.12 (− 3.15, − 3.09)	18.92 (12.15, 25.69)	− 0.83 (− 0.83, − 0.83)
Andean Latin America	14.21 (5.65, 22.77)	− 2.14 (− 2.15, − 2.13)	17.71 (5.02, 30.41)	− 0.43 (− 0.45, − 0.40)
Caribbean	46.19 (26.16, 66.21)	− 0.23 (− 0.23, − 0.22)	45.49 (25.81, 65.17)	0.05 (0.05, 0.06)
Central Latin America	6.13 (1.78, 10.48)	− 4.11 (− 4.14, − 4.08)	8.18 (4.95, 11.41)	− 1.46 (− 1.47, − 1.45)
Tropical Latin America	20.78 (13.53, 28.02)	− 2.80 (− 2.84, − 2.77)	30.95 (18.13, 43.77)	− 0.53 (− 0.53, − 0.52)
North Africa and Middle East	21.69 (16.53, 26.86)	− 2.53 (− 2.55, − 2.50)	20.94 (15.89, 25.99)	− 2.31 (− 2.33, − 2.28)
North Africa and Middle East	21.69 (16.53, 26.86)	− 2.53 (− 2.55, − 2.50)	20.94 (15.89, 25.99)	− 2.31 (− 2.33, − 2.28)
South Asia	58.88 (33.96, 83.81)	− 0.93 (− 0.93, − 0.92)	52.24 (30.62, 73.85)	− 1.03 (− 1.03, − 1.03)
South Asia	58.88 (33.96, 83.81)	− 0.93 (− 0.93, − 0.92)	52.24 (30.63, 73.85)	− 1.03 (− 1.03, − 1.03)
Southeast Asia, East Asia, and Oceania	14.13 (3.96, 24.30)	− 1.09 (− 1.11, − 1.07)	11.67 (3.67, 19.68)	− 0.92 (− 0.95, − 0.89)
East Asia	4.06 (1.96, 6.16)	− 3.39 (− 3.42, − 3.36)	3.95 (1.56, 6.34)	− 2.83 (− 2.83, − 2.82)
Oceania	43.66 (1.19, 91.51)	− 0.13 (− 0.16, − 0.11)	48.45 (22.67, 74.24)	0.11 (0.07, 0.14)
Southeast Asia	27.42 (17.81, 37.03)	− 0.76 (− 0.77, − 0.75)	19.66 (12.71, 26.62)	− 0.91 (− 0.92, − 0.90)
Sub-Saharan Africa	84.13 (51.76, 116.50)	− 1.14 (− 1.15, − 1.13)	32.42 (21.98, 42.86)	− 1.34 (− 1.35, − 1.32)
Central sub-Saharan Africa	19.83 (14.67, 24.99)	− 2.98 (− 3.00, − 2.95)	10.53 (6.84, 14.21)	− 3.50 (− 3.55, − 3.46)
Eastern sub-Saharan Africa	33.75 (22.73, 44.77)	− 1.93 (− 1.95, − 1.92)	21.11 (13.18, 29.04)	− 2.11 (− 2.12, − 2.09)
Southern sub-Saharan Africa	48.53 (13.69, 83.37)	− 0.82 (− 0.83, − 0.81)	36.69 (14.80, 58.57)	− 0.72 (− 0.72, − 0.71)
Western sub-Saharan Africa	169.29 (91.06, 247.52)	− 0.64 (− 0.64, − 0.64)	59.12 (35.63, 82.61)	− 0.25 (− 0.25, − 0.25)

*ASR* age-standardized rate; *CI* confidence interval; *DALYs* disability-adjusted life-years; *EAPC* estimated annual percentage change; *GBD* global burden of disease; *rIDPs* rare infectious diseases of poverty; *SDI* socio-demographic index; *YLD* years lived with disability

^a^EAPC was used to quantify the estimated changing trends from 2022 to 2050. A linear regression model was fitted based on the natural logarithm of the ASR (*y* = *α* + *βx* + *ε*), where *y* was equal to logarithmically transformed ASR. EAPC was calculated as 100 × (*e*^*β*^ − 1), and 95% *CI* was obtained from the linear regression model

## Discussion

This study summarized the number and ASRs of prevalence, mortality, DALYs, YLDs, and YLLs of rIDPs with comparisons of temporal trends and heterogeneity in SDI levels, regions, countries and territories, age, and sex. Overall, in 2021, the burden of rIDPs varied by age, sex, SDI, and region, especially higher in the groups aged below 15 years, females, and regions with low SDI values, such as sub-Saharan Africa and South Asia. Globally, there were slight declines in the ASPRs, age-standardized DALY rates, and age-standardized YLD rates of rIDPs, and slightly increasing trends of ASMR and age-standardized YLL rates from 1990 to 2021.

### Efforts of rIDP control in the past decades and potential risks in the future

The generally slight change in the disease burden of rIDPs indicated insufficient efforts on rIDPs over the past three decades. The results could be disentangled into several aspects. The slightly declined ASPRs, age-standardized DALY rates, and age-standardized YLD rates of rIDPs might be involved in the fact that rIDPs have gained unprecedented support around the world, such as the launch of the NTDs Road Map by WHO and the release of the London Declaration with certain actions in early detection and treatment of cases, vector management, social mobilization, monitoring, and operational research. These actions and strategies have made significant contributions to the global momentum and reduced the burden of rIDPs [26–29]. Despite of substantial efforts, the effectiveness of rIDPs control was disproportionally shown in regions with different SDI levels. Specifically, regions with low SDI values received less progress than regions with high SDI levels. It suggested the requirement for long-term interventions of rIDPs in those vulnerable areas. In addition, there were slightly increasing trends of ASMRs with twists and turns between 1990 and 2021 in either low, low-middle, or even high SDI regions. With the increased human mobility, economic downturn, and outbreak of wars in various countries, there is a possibility of rIDPs recurrence and our findings suggest that the recurrence could occur even in low-burden regions, such as high SDI areas [12]. The recurrence risks require a more rational allocation of health resources to promote the capacity of local communities on disease diagnosis and treatment in poor areas and improve the awareness of rIDPs’ recurrence in regions even with a lower disease burden [30].

### Vulnerable populations of rIDPs

In 2021, a higher age-standardized DALY rate among the population aged lower than 15 years, particularly among those aged lower than 5 years, might be attributed to insufficient healthcare coverage among this group. Immunization and joint delivery of preventive chemotherapy are the key interventions for this population. However, the recent progression report indicated it is still far from achieving a satisfactory intervention coverage rate for several certain rIDPs [7]. More efforts should be engaged with cross-sectional approaches to promote mass vaccination, drug administration, and health education for those vulnerable populations. The higher burden of rIDPs in females might be explained by gender role factors, such as caring for infected children, rather than biological ones [31]. Meanwhile, rIDPs might disproportionately influence women, making them experience more stigma and discrimination and affecting their fertility and mental health [32]. The local governments, for areas suffering high disease burden of IDPs, such as sub-Saharan Africa and South Asia, should pay more attention to the vulnerable population of children and women, enhance the locals’ awareness of rIDPs, take actions to reduce the stigma of patients, and strengthen the targeted monitoring and prevention, which not only improves the equity of the rIDPs programmes, but also improves the efficacy of overall elimination and control strategies.

### Challenges and potential solutions in the prevention and control of rIDPs

rIDPs in this study included more than 100 subtypes of NTDs and most of them were not listed as priorities of prevention, control, elimination, or eradication. Because of the rarity of single rIDP subtype, the prevalence, mortality, and other data could be extremely low, leading to the aggregation of these diseases as rIDPs for analyses in this study. It should be noted that the aggregated burden of rIDPs accounted for over 25% of disease burdens of total NTDs [17], suggesting the importance of identifying universal control strategies for those groups of diseases. So far, the vast majority of rIDPs lack targeted detection tools, effective vaccination, and therapeutic drugs against pathogens [33]. Most responses against rIDPs require joint interventions aligned with economic development—better housing, sanitation, and public hygiene—alongside increased government investment. Given a complex interplay of factors, including poverty, environmental degradation, and changing ecosystems, marginalized people are more vulnerable to those rIDPs, causing huge economic losses through the establishment of vicious circles of disease, and reduced work ability and poverty [34]. To reach disenfranchised populations who continue to struggle with the burden of poverty and diseases and to reduce the risk of zoonotic disease spillover and transmission, integrated control strategies with the One Health concept are needed. These strategies, which consider the holistic well-being of humans, animals, and the environment, are expected to strengthen health infrastructure and improve service delivery in impoverished areas by ensuring safe drinking water, decent sanitation, and effective waste disposal [35–37].

The findings would raise awareness of rIDPs, which should be listed as the local poverty priority. Health systems need to be more positive in dealing with them to break the vicious cycle between rIDPs and poverty, especially among remote and marginalized populations. For local governments, the priorities of rIDPs prevention and control should be improved with strong political incentives and high investment. Effective allocation of health resources can be highly cost-effective, resulting in both direct savings (such as reduced medical costs) and indirect savings (through increased productivity and reduced losses in work time), and further promoting the achievement of SDGs. Although there have been several innovative diagnostic methods for rIDPs [38], there are still no targeted first-line, oral drugs for widespread access to early treatment for most rIDPs [28]. Endemic countries and pharmaceutical companies should cooperate more closely to further improve the large-scale availability of drugs or commodities, for human or animal use, such as taeniasis and trichinellosis [39].

### The road map for achieving SDG goals

**Fig. 5:**
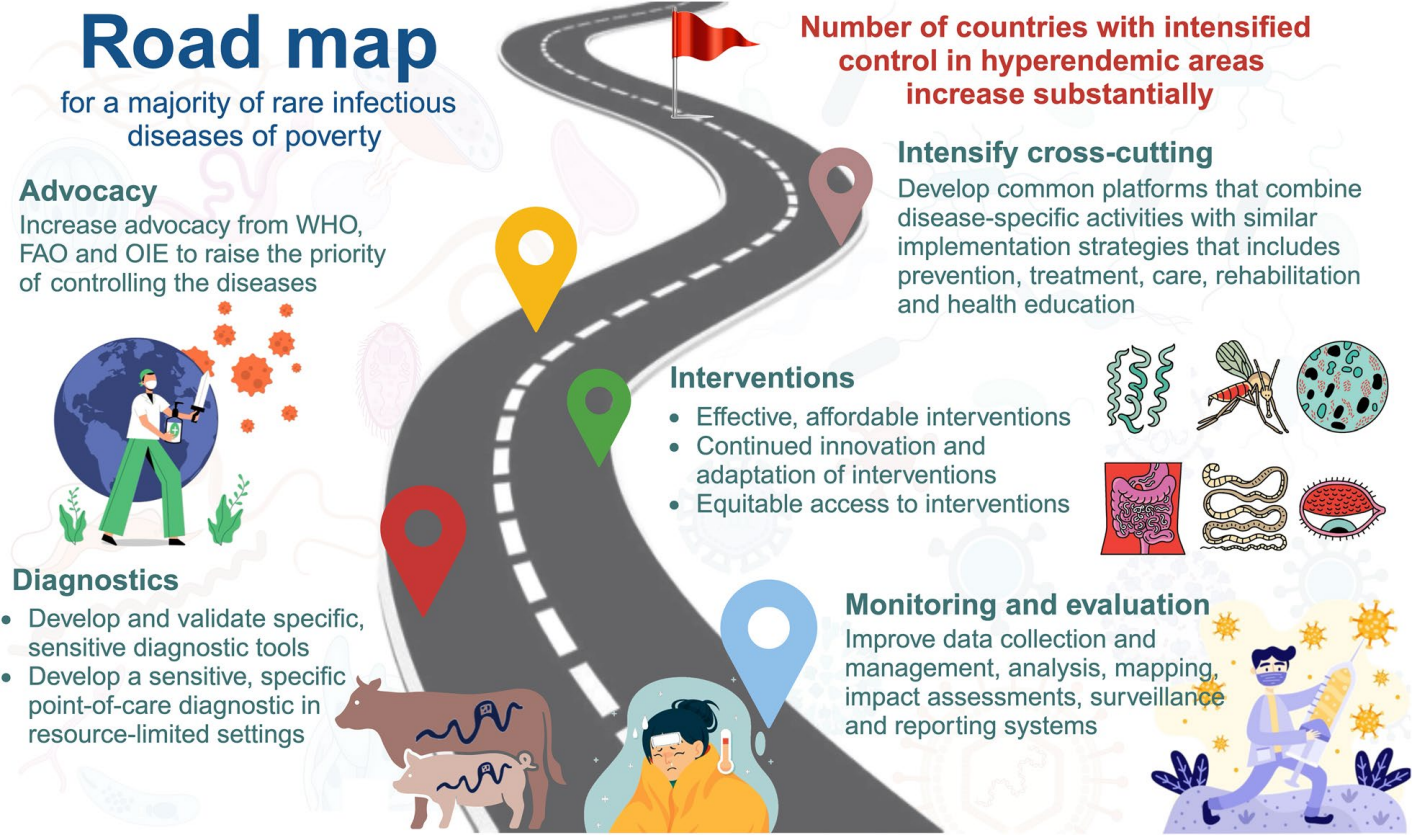
Road map for majority of rare infectious diseases of poverty. In order from the bottom up, measures are implemented in aspects of monitoring and evaluation, diagnosis, interventions, advocacy, and intensified cross-cutting to gradually achieve the ultimate goal, which is the number of countries with intensified control in hyperendemic areas increases substantially

Despite some concerns raised that COVID-19 would disrupt the progression of ending rIDPs, this study did not identify clear changes in the disease burden of rIDPs before and after 2020. However, there remained several targets failed to be achieved for rIDPs and NTDs in 2020. WHO released an updated road map for ending NTDs during 2021–2030 to achieve SDG, which proposed a 75% reduction in NTD DALYs by 2030 [26]. However, there was only an 11% reduction in NTDs between 2015 and 2019. Our estimation only showed an − 0.16% AAPC reduction in the age-standardized DALY rate of rIDPs from 1990 to 2021, and the projected results by 2050 suggest the target of SDG would be hard to achieve if only following the current strength of intervention. It is noted that the projection estimates for 2022–2050, particularly in sub-regions, should be cautiously explained because the estimates suffer from large uncertainty. The reason might be small variations of previous trends, the sparse nature of data, and the large uncertainty of the estimation in 1990–2021. Nevertheless, the results still highlight more tightened engagement in rIDPs prevention and interventions for DALY reduction to achieve the SDG targets. Modified by the road map of NTDs, we drew up a road map for actions to prevent and control rIDPs (Fig. 5). The main objective of the road map is to enable governments to lead the way in implementing rIDPs interventions through a cross-cutting approach. For example, the number of countries with enhanced control in areas with a high prevalence of taeniasis needs to increase from 2 to 17 in 2020–2030. Accordingly, surveillance and evaluation of rIDPs are essential for the achievement of 2030 targets, national governments can develop more cost-effective information-gathering strategies to provide support for carrying out group medicine-taking activities. In addition, two priorities for diagnosis were pointed out: (i) the development and validation of specific, sensitive diagnostic tools for porcine cysticercosis; and (ii) the development of a sensitive, specific point-of-care diagnosis for human taeniasis and neurocysticercosis in a resource-limited environment. To enhance control efforts, it is valuable to develop high-throughput tests to assist in mapping endemic regions, and evaluate the effectiveness of targeted interventions in high-endemicity and resource-constrained areas [40]. Moreover, great importance should be attached to the advocacy from the WHO, Food and Agriculture Organization of the United Nations, and World Organization for Animal Health to raise the priority of controlling diseases. To combine the activities of rIDPs with similar delivery strategies, an integrated platform based on artificial intelligence is demanded, which could provide support for the most neglected rIDPs, ensuring that they are systematically addressed and that action is proportionate to need.

### Limitations

This study has several limitations. First, although GBD has a wide range of inclusion criteria for various data sources, data are not available for certain locations to capture true variations across geography, sex, and time, and estimates are based on predictive covariates or trends from adjacent locations, which might bias the results, particularly in low- and middle-income countries. More epidemiological studies on rIDPs to inform and improve the future estimates of the disease burden are in demand, including more data to inform the disability weights and health status of rIDPs. Second, the analysis might underestimate the burden of rIDPs since a number of individuals with rIDPs might not seek medical attention and evade epidemiological assessment. Moreover, poor areas lacked the requisite healthcare facilities for rIDPs’ diagnosis, which would result in the underestimation of current burden estimates for those regions. Such underreporting or underdiagnosis could not be fully addressed by GBD modeling, where the estimates depend on the quality and quantity of input data. Third, the time lag between the date of data collection and the database input might lead to delays in the evaluation of the rIDPs. Fourth, in the GBD studies, racial differences were ignored, while it has a far-reaching influence on the disease onset and death to a certain degree. Further studies on the etiology and risk factors of rIDPs are needed. Last, due to unavailable incidence rates of rIDPs in the GBD database, we used limited indicators, including prevalence, mortality, DALYs, YLLs, and YLDs, to evaluate the burden of rIDPs, which produced incomplete results. We are looking forward to the governments or communities investing more health resources for rIDPs to obtain more information, trace the disease situations timely and control the development of rIDPs.

## Conclusions

The rIDPs remained a major challenge for global public health in 2021, especially in sub-Saharan Africa and South Asia regions. The burden of rIDPs was mainly distributed in the age group under 15 years, females, and regions with lower SDI levels. From 1990 to 2021, the study observed the decline of the global trends of rIDPs’ ASPR, age-standardized DALY rate, and age-standardized YLD rate, whereas there was a mild increase in ASMR. The slight decline in the burden of rIDPs suggests that achieving the SDG target by 2030 might be challenging. It required more powerful strategies to enhance and expand rIDPs surveillance and interventions at the regional or national level, and to formulate targeted strategies for poverty eradication.

## Supplementary Information

**Additional file 1**. Including additional results: **Table S1.** Countries/territories grouped by socio-demographic index. **Table S2**. Regional categories of 204 countries/territories by GBD. **Table S3**. Definition and ICD-10 code of rIDPs. **Table S4**. Numbers and age-standardized prevalence rates (per 100,000 population) of rIDPs, and the percentage changes in the age-standardized rates, by sex, SDI levels, GBD super regions and sub-regions, from 1990 to 2021. **Table S5**. Age-standardized mortality and YLL rates (per 100,000 population) of rIDPs and the percentage changes in the age-standardized rates, by sex, SDI levels, GBD super regions and sub-regions, from 1990 to 2021. **Table S6**. Number of mortality and DALYs of rIDPs by sex, SDI levels, GBD super regions and sub-regions, from 1990 to 2021. **Table S7**. Number of YLLs and YLDs of rIDPs by sex, SDI levels, GBD super regions and sub-regions, from 1990 to 2021. **Table S8**. Temporal trends in age-standardized prevalence rates of rIDPs by sex, SDI levels, and GBD super regions, from 1990 to 2021. **Table S9**. Temporal trends in age-standardized mortality rates of rIDPs by sex, SDI levels, and GBD super regions, from 1990 to 2021. **Table S10**. Temporal trends in age-standardized DALY rates of rIDPs by GBD super regions, from 1990 to 2021. **Table S11**. Temporal trends in age-standardized YLD rates of rIDPs by sex, SDI levels, and GBD super regions, from 1990 to 2021. **Table S12**. Temporal trends in age-standardized YLL rates of rIDPs by sex, SDI levels, and GBD super regions, from 1990 to 2021. **Table S13**. Global distribution of age-standardized prevalence, mortality, DALY, YLD, and YLL rates (per 100,000 population) of rIDPs by countries and territories in 2021, and the percentage changes in the age-standardized rates from 1990 to 2021. **Table S14**. Temporal trends in age-standardized prevalence, mortality, DALY, YLD, and YLL rates of rIDPs by countries and territories, from 1990 to 2021. **Table S15**. Predicted age-standardized prevalence, mortality, and YLL rates (per 100,000 population) of rIDPs from 2022–2050 with standard deviation, by sex, SDI levels, GBD super regions and sub-regions, based on the Bayesian age-period-cohort model. **Fig. S1** Association between age-standardized prevalence rates (per 100,000 population) of rIDPs and SDI values by countries and territories and GBD super regions, in 2021. **Fig. S2** Association between age-standardized mortality rates (per 100,000 population) of rIDPs and SDI values by countries and territories and GBD super regions in 2021. **Fig. S3** Association between age-standardized YLL rates (per 100,000 population) of rIDPs and SDI values by countries and territories and GBD super regions in 2021. **Fig. S4** Temporal trends of age-standardized prevalence, mortality, and YLL rates of rIDPs by SDI levels from 1990 to 2021. **Fig. S5** Age-standardized prevalence, mortality, and YLL rates (per 100,000 population) of rIDPs by age, sex, and SDI levels in 2021.

**Additional file 2**: Table. ISO-3 country code.

## Data Availability

The datasets analyzed during the current study are available at http://ghdx.healthdata.org/gbd-results-tool and https://population.un.org/wpp/.

## Abbreviations

AAPC: Average annual percentage change
APC: Annual percent change
ASPR: Age-standardized prevalence rate
ASMR: Age-standardized mortality rate
ASR: Age-standardized rate
BAPC: Bayesian age-period-cohort
*CI*: Confidence interval
DALY: Disability-adjusted life-year
EAPC: Estimated annual percentage change
GBD: Global Burden of Disease
GSM: Grid search method
ICD: International Classification of Diseases
IDP: Infectious disease of poverty
LOWESS: Locally Weighted Scatterplot Smoothing
NTD: Neglected tropical disease
rIDP: Rare infectious disease of poverty
SD: Standard deviation
SDG: Sustainable Development Goal
SDI: Socio-demographic Index
UI: Uncertainty interval
WHO: World Health Organization
WPP: World Population Prospects
YLD: Year lived with disability
YLL: Year of life lost
