# Supplementary material for: Global burden associated with rare infectious diseases of poverty in 2021: findings from the Global Burden of Disease Study 2021
Source: Infect Dis Poverty. 2024 Nov 13;13:85. doi: 10.1186/s40249-024-01249-6 (PMC11558835; doi:10.1186/s40249-024-01249-6)
Supplement: Supplementary file 1 — Additional file 1. Including additional results: Table S1. Countries/territories grouped by socio-demographic index. Table S2. Regional categories of 204 countries/territories by GBD. Table S3. Definition and ICD-10 code of rIDPs. Table S4. Numbers and age-standardized prevalence rates (per 100,000 population) of rIDPs, and the percentage changes in the age-standardized rates, by sex, SDI levels, GBD super regions and sub-regions, from 1990 to 2021. Table S5. Age-standardized mortality and YLL rates (per 100,000 population) of rIDPs and the percentage changes in the age-standardized rates, by sex, SDI levels, GBD super regions and sub-regions, from 1990 to 2021. Table S6. Number of mortality and DALYs of rIDPs by sex, SDI levels, GBD super regions and sub-regions, from 1990 to 2021. Table S7. Number of YLLs and YLDs of rIDPs by sex, SDI levels, GBD super regions and sub-regions, from 1990 to 2021. Table S8. Temporal trends in age-standardized prevalence rates of rIDPs by sex, SDI levels, and GBD super regions, from 1990 to 2021. Table S9. Temporal trends in age-standardized mortality rates of rIDPs by sex, SDI levels, and GBD super regions, from 1990 to 2021. Table S10. Temporal trends in age-standardized DALY rates of rIDPs by GBD super regions, from 1990 to 2021. Table S11. Temporal trends in age-standardized YLD rates of rIDPs by sex, SDI levels, and GBD super regions, from 1990 to 2021. Table S12. Temporal trends in age-standardized YLL rates of rIDPs by sex, SDI levels, and GBD super regions, from 1990 to 2021. Table S13. Global distribution of age-standardized prevalence, mortality, DALY, YLD, and YLL rates (per 100,000 population) of rIDPs by countries and territories in 2021, and the percentage changes in the age-standardized rates from 1990 to 2021. Table S14. Temporal trends in age-standardized prevalence, mortality, DALY, YLD, and YLL rates of rIDPs by countries and territories, from 1990 to 2021. Table S15. Predicted age-standardized prevalence, mortalit [file 40249_2024_1249_MOESM1_ESM.docx]

Additional file 1

**Global burden associated with rare infectious diseases of poverty in 2021: findings from the Global Burden of Disease Study 2021**

[Table S1. Countries/territories grouped by socio-demographic index 3](#_Toc178164059)

[Table S2. Regional categories of 204 countries/territories by GBD. 4](#_Toc178164060)

[Table S3. Definition and ICD-10 code of rIDPs 5](#_Toc178164061)

[Table S4. Numbers and age-standardized prevalence rates (per 100,000 population) of rIDPs, and the percentage changes in the age-standardized rates, by sex, SDI levels, GBD super regions and sub-regions, from 1990 to 2021 8](#_Toc178164062)

[Table S5. Age-standardized mortality and YLL rates (per 100,000 population) of rIDPs and the percentage changes in the age-standardized rates, by sex, SDI levels, GBD super regions and sub-regions, from 1990 to 2021 10](#_Toc178164063)

[Table S6. Number of mortality and DALYs of rIDPs by sex, SDI levels, GBD super regions and sub-regions, from 1990 to 2021 12](#_Toc178164064)

[Table S7. Number of YLLs and YLDs of rIDPs by sex, SDI levels, GBD super regions and sub-regions, from 1990 to 2021 14](#_Toc178164065)

[Table S8. Temporal trends in age-standardized prevalence rates of rIDPs by sex, SDI levels, and GBD super regions, from 1990 to 2021. 16](#_Toc178164066)

[Table S9. Temporal trends in age-standardized mortality rates of rIDPs by sex, SDI levels, and GBD super regions, from 1990 to 2021. 18](#_Toc178164067)

[Table S10. Temporal trends in age-standardized DALY rates of rIDPs by GBD super regions, from 1990 to 2021. 20](#_Toc178164068)

[Table S11. Temporal trends in age-standardized YLD rates of rIDPs by sex, SDI levels, and GBD super regions, from 1990 to 2021. 21](#_Toc178164069)

[Table S12. Temporal trends in age-standardized YLL rates of rIDPs by sex, SDI levels, and GBD super regions, from 1990 to 2021. 23](#_Toc178164070)

[Table S13. Global distribution of age-standardized prevalence, mortality, DALY, YLD, and YLL rates (per 100,000 population) of rIDPs by countries and territories in 2021, and the percentage changes in the age-standardized rates from 1990 to 2021 25](#_Toc178164071)

[Table S14. Temporal trends in age-standardized prevalence, mortality, DALY, YLD, and YLL rates (per 100,000 population) of rIDPs by countries and territories, from 1990 to 2021. 36](#_Toc178164072)

[Table S15. Predicted age-standardized prevalence, mortality, and YLL rates (per 100,000 population) of rIDPs from 2022-2050 with standard deviation, by sex, SDI levels, GBD super regions and sub-regions, based on the Bayesian age-period-cohort model 43](#_Toc178164073)

[Fig. S1 Association between age-standardized prevalence rates (per 100,000 population) of rIDPs and SDI values by countries and territories and GBD super regions in 2021. 45](#_Toc178164074)

[Fig. S2 Association between age-standardized mortality rates (per 100,000 population) of rIDPs and SDI values by countries and territories and GBD super regions in 2021. 46](#_Toc178164075)

[Fig. S3 Association between age-standardized YLL rates (per 100,000 population) of rIDPs and SDI values by countries and territories and GBD super regions in 2021. 47](#_Toc178164076)

[Fig. S4 Temporal trends of age-standardized prevalence, mortality, and YLL rates of rIDPs by SDI levels from 1990 to 2021. 48](#_Toc178164077)

[Fig. S5 Age-standardized prevalence, mortality, and YLL rates (per 100,000 population) of rIDPs by age, sex, and SDI levels in 2021. 49](#_Toc178164078)

# Table S1. Countries/territories grouped by socio-demographic index

| **SDI-quintile** | **lower-upper bound** | **Countries and territories** |
| --- | --- | --- |
| Low SDI | 0.0000-0.4658 | Afghanistan, Benin, Burkina Faso, Burundi, Central African Republic, Chad, Côte d'Ivoire, Democratic Republic of the Congo, Eritrea, Ethiopia, Gambia, Guinea, Guinea-Bissau, Haiti, Liberia, Madagascar, Malawi, Mali, Mozambique, Nepal, Niger, Papua New Guinea, Rwanda, Senegal, Sierra Leone, Solomon Islands, Somalia, South Sudan, Tanzania, Timor-Leste, Togo, Uganda, Yemen |
| Low-middle SDI | 0.4658-0.6188 | Angola, Bangladesh, Belize, Bhutan, Bolivia, Cabo Verde, Cambodia, Cameroon, Comoros, Congo (Brazzaville), Djibouti, Egypt, El Salvador, Eswatini, Federated States of Micronesia, Ghana, Guatemala, Honduras, India, Kenya, Kiribati, Kyrgyzstan, Laos, Lesotho, Marshall Islands, Mauritania, Mongolia, Morocco, Myanmar, Namibia, Nicaragua, Nigeria, North Korea, Pakistan, Samoa, São Tomé and Príncipe, Sudan, Tajikistan, Tuvalu, Vanuatu, Venezuela, Zambia, Zimbabwe |
| Middle SDI | 0.6188-0.7120 | Albania, Algeria, Armenia, Azerbaijan, Botswana, Brazil, Colombia, Costa Rica, Cuba, Dominican Republic, Ecuador, Equatorial Guinea, Fiji, Gabon, Grenada, Guyana, Indonesia, Iran, Iraq, Jamaica, Maldives, Mexico, Nauru, Palestine, Panama, Paraguay, Peru, Philippines, Saint Lucia, Saint Vincent and the Grenadines, South Africa, Sri Lanka, Suriname, Syria, Thailand, Tokelau, Tonga, Tunisia, Turkmenistan, Uzbekistan, Viet Nam |
| High-middle SDI | 0.7120-0.8103 | American Samoa, Antigua and Barbuda, Argentina, Bahamas, Bahrain, Barbados, Belarus, Bosnia and Herzegovina, Brunei, Bulgaria, Chile, China, Cook Islands, Croatia, Dominica, Georgia, Greece, Guam, Hungary, Israel, Italy, Jordan, Kazakhstan, Lebanon, Libya, Malaysia, Malta, Mauritius, Moldova, Montenegro, Niue, North Macedonia, Northern Mariana Islands, Oman, Palau, Portugal, Romania, Russia, Saint Kitts and Nevis, Serbia, Seychelles, Slovakia, Spain, Trinidad and Tobago, Türkiye, Ukraine, Uruguay, |
| High SDI | 0.8103-1.0000 | Andorra, Australia, Austria, Belgium, Bermuda, Canada, Cyprus, Czechia, Denmark, Estonia, Finland, France, Germany, Greenland, Iceland, Ireland, Japan, Kuwait, Latvia, Lithuania, Luxembourg, Monaco, Netherlands, New Zealand, Norway, Poland, Puerto Rico, Qatar, San Marino, Saudi Arabia, Singapore, Slovenia, South Korea, Sweden, Switzerland, Taiwan (Province of China), United Arab Emirates, United Kingdom, United States of America, Virgin Islands |

Abbreviation: SDI: socio-demographic index

# Table S2. Regional categories of 204 countries/territories by GBD.

| **Super regions** | **21 sub regions** | **Countries and territories** |
| --- | --- | --- |
| Central Europe, Eastern Europe, and Central Asia | Central Europe | Albania, Bosnia and Herzegovina, Bulgaria, Croatia, Czechia, Hungary, Montenegro, North Macedonia, Poland, Romania, Serbia, Slovakia, Slovenia |
|  | Eastern Europe | Belarus, Estonia, Latvia, Lithuania, Moldova, Russia, Ukraine |
|  | Central Asia | Armenia, Azerbaijan, Georgia, Kazakhstan, Kyrgyzstan, Mongolia, Tajikistan, Turkmenistan, Uzbekistan |
| High Income | Australasia | Australia, New Zealand |
|  | High-income Asia Pacific | Brunei, Japan, South Korea, Singapore |
|  | High-income North America | Canada, Greenland, USA |
|  | Southern Latin America | Argentina, Chile, Uruguay |
|  | Western Europe | Andorra, Austria, Belgium, Cyprus, Denmark, Finland, France, Germany, Greece, Iceland, Ireland, Israel, Italy, Luxembourg, Malta, Monaco, Netherlands, Norway, Portugal, San Marino, Spain, Sweden, Switzerland, UK |
| Latin America and Caribbean | Andean Latin America | Bolivia, Ecuador, Peru |
|  | Central Latin America | Colombia, Costa Rica, El Salvador, Guatemala, Honduras, Mexico, Nicaragua, Panama, Venezuela |
|  | Tropical Latin America | Brazil, Paraguay |
|  | Caribbean | Antigua and Barbuda, Bahamas, Barbados, Belize, Bermuda, Cuba, Dominica, Dominican Republic, Grenada, Guyana, Haiti, Jamaica, Puerto Rico, Saint Kitts and Nevis, Saint Lucia, Saint Vincent and the Grenadines, Suriname, Trinidad and Tobago, Virgin Islands |
| North Africa and Middle East | North Africa and Middle East | Afghanistan, Algeria, Bahrain, Egypt, Iran, Iraq, Jordan, Kuwait, Lebanon, Libya, Morocco, Oman, Palestine, Qatar, Saudi Arabia, Sudan, Syria, Tunisia, Türkiye, United Arab Emirates, Yemen |
| South Asia | South Asia | Bangladesh, Bhutan, India, Nepal, Pakistan |
| South-East Asia, East Asia, and Oceania | East Asia | China, North Korea, Taiwan (province of China), |
|  | Oceania | American Samoa, Cook Islands, Federated States of Micronesia, Fiji, Guam, Kiribati, Marshall Islands, Nauru, Niue, Northern Mariana Islands, Palau, Papua New Guinea, Samoa, Solomon Islands, Tokelau, Tonga, Tuvalu, Vanuatu |
|  | Southeast Asia | Cambodia, Indonesia, Laos, Malaysia, Maldives, Mauritius, Myanmar, Philippines, Seychelles, Sri Lanka, Thailand, Timor-Leste, Viet Nam |
| Sub-Saharan Africa | Central sub-Saharan Africa | Angola, Central African Republic, Congo (Brazzaville), DR Congo, Equatorial Guinea, Gabon |
|  | Eastern sub-Saharan Africa | Burundi, Comoros, Djibouti, Eritrea, Ethiopia, Kenya, Madagascar, Malawi, Mozambique, Rwanda, Somalia, South Sudan, Uganda, Tanzania, Zambia |
|  | Southern sub-Saharan Africa | Botswana, Eswatini, Lesotho, Namibia, South Africa, Zimbabwe |
|  | Western sub-Saharan Africa | Benin, Burkina Faso, Cabo Verde, Cameroon, Chad, Côte d'Ivoire, The Gambia, Ghana, Guinea, Guinea-Bissau, Liberia, Mali, Mauritania, Niger, Nigeria, São Tomé and Príncipe, Senegal, Sierra Leone, Togo |

Abbreviation: GBD, global burden of disease.

# Table S3. Definition and ICD-10 code of rIDPs

| ICD-10 | Disease |
| --- | --- |
| A68 | Relapsing fevers |
| A68.0 | Louse-borne relapsing fever |
| A68.1 | Tick-borne relapsing fever |
| A68.9 | Relapsing fever, unspecified |
| A69.2 | Lyme disease |
| A69.8 | Other specified spirochaetal infections |
| A69.9 | Spirochaetal infection, unspecified |
| A75 | Typhus fever |
| A75.0 | Epidemic louse-borne typhus fever due to Rickettsia prowazekii |
| A75.1 | Recrudescent typhus [Brill disease] |
| A75.2 | Typhus fever due to Rickettsia typhi |
| A75.3 | Typhus fever due to Rickettsia tsutsugamushi |
| A75.9 | Typhus fever, unspecified |
| A77 | Spotted fever |
| A77.0 | Spotted fever due to Rickettsia rickettsii |
| A77.1 | Spotted fever due to Rickettsia conorii |
| A77.2 | Spotted fever due to Rickettsia sibirica |
| A77.3 | Spotted fever due to Rickettsia australis |
| A77.8 | Other spotted fevers |
| A77.9 | Spotted fever, unspecified |
| A92 | Other mosquito-borne viral fevers |
| A92.0 | Chikungunya virus disease |
| A92.1 | O'nyong-nyong fever |
| A92.2 | Venezuelan equine fever |
| A92.3 | West Nile virus infection |
| A92.4 | Rift Valley fever |
| A92.5 | Zika virus disease |
| A92.8 | Other specified mosquito-borne viral fevers |
| A92.9 | Mosquito-borne viral fever, unspecified |
| A93 | Other arthropod-borne viral fevers, not elsewhere classified |
| A93.0 | Oropouche virus disease |
| A93.1 | Sandfly fever |
| A93.2 | Colorado tick fever |
| A93.8 | Other specified arthropod-borne viral fevers |
| A94 | Unspecified arthropod-borne viral fever |
| A96 | Arenaviral haemorrhagic fever |
| A96.0 | Junin haemorrhagic fever |
| A96.1 | Machupo haemorrhagic fever |
| A96.2 | Lassa fever |
| A96.8 | Other arenaviral haemorrhagic fevers |
| A96.9 | Arenaviral haemorrhagic fever, unspecified |
| A98 | Other viral haemorrhagic fevers, not elsewhere classified |
| A98.0 | Crimean-Congo haemorrhagic fever |
| A98.1 | Omsk haemorrhagic fever |
| A98.2 | Kyasanur Forest disease |
| A98.3 | Marburg virus disease |
| A98.5 | Haemorrhagic fever with renal syndrome |
| A98.8 | Other specified viral haemorrhagic fevers |
| A99 | Unspecified viral haemorrhagic fever |
| B33.0 | Epidemic myalgia |
| B33.1 | Ross River disease |
| B60 | Other protozoal diseases, not elsewhere classified |
| B60.0 | Babesiosis |
| B60.1 | Acanthamoebiasis |
| B60.2 | Naegleriasis |
| B60.8 | Other specified protozoal diseases |
| B64 | Unspecified protozoal disease |
| B67.5 | Echinococcus multilocularis infection of liver |
| B67.6 | Echinococcus multilocularis infection, other and multiple sites |
| B67.7 | Echinococcus multilocularis infection, unspecified |
| B68 | Taeniasis |
| B68.0 | Taenia solium taeniasis |
| B68.1 | Taenia saginata taeniasis |
| B68.9 | Taeniasis, unspecified |
| B70 | Diphyllobothriasis and sparganosis |
| B70.0 | Diphyllobothriasis |
| B70.1 | Sparganosis |
| B71 | Other cestode infections |
| B71.0 | Hymenolepiasis |
| B71.1 | Dipylidiasis |
| B71.8 | Other specified cestode infections |
| B71.9 | Cestode infection, unspecified |
| B74.3 | Loiasis |
| B74.4 | Mansonelliasis |
| B74.8 | Other filariases |
| B74.9 | Filariasis, unspecified |
| B75 | Trichinellosis |
| B78 | Strongyloidiasis |
| B78.0 | Intestinal strongyloidiasis |
| B78.1 | Cutaneous strongyloidiasis |
| B78.7 | Disseminated strongyloidiasis |
| B78.9 | Strongyloidiasis, unspecified |
| B80 | Enterobiasis |
| B81 | Other intestinal helminthiases, not elsewhere classified |
| B81.0 | Anisakiasis |
| B81.1 | Intestinal capillariasis |
| B81.2 | Trichostrongyliasis |
| B81.3 | Intestinal angiostrongyliasis |
| B81.4 | Mixed intestinal helminthiases |
| B81.8 | Other specified intestinal helminthiases |
| B82 | Unspecified intestinal parasitism |
| B82.0 | Intestinal helminthiasis, unspecified |
| B82.9 | Intestinal parasitism, unspecified |
| B83 | Other helminthiases |
| B83.0 | Visceral larva migrans |
| B83.1 | Gnathostomiasis |
| B83.2 | Angiostrongyliasis due to Parastrongylus cantonensis |
| B83.3 | Syngamiasis |
| B83.4 | Internal hirudiniasis |
| B83.8 | Other specified helminthiases |
| B83.9 | Helminthiasis, unspecified |
| B89 | Unspecified parasitic disease |
| P37.1 | Congenital toxoplasmosis |

Abbreviation: ICD, International Classification of Diseases; rIDPs, rare infectious diseases of poverty.

# Table S4. Numbers and age-standardized prevalence rates (per 100,000 population) of rIDPs, and the percentage changes in the age-standardized rates, by sex, SDI levels, GBD super regions and sub-regions, from 1990 to 2021

| **Location** | **Prevalence,**  **Number** | | **Prevalence,**  **per 100,000 population** | | |
| --- | --- | --- | --- | --- | --- |
|  | **1990**  **(95% UI)** | **2021**  **(95% UI)** | **1990**  **(95% UI)** | **2021**  **(95% UI)** | **change, %^a^**  **(95% UI)** |
| Global | 84530285.39  (83248717.34, 85748168.82) | 103762442.30  (102134927.45, 105436286.84) | 1504.49 (1482.51, 1525.10) | 1381.18 (1358.63, 1403.46) | -8.20 (-9.94, -6.20) |
| Female | 50571171.38  (49785525.88, 51337595.07) | 67391508.79  (66187518.75, 68578666.53) | 1840.26 (1812.20, 1867.93) | 1794.6 (1761.99, 1825.37) | -2.48 (-4.61, -0.52) |
| Male | 33959114.01  (33098474.82, 34787728.71) | 36370933.51  (35220784.66, 37577359.52) | 1180.89 (1152.81, 1207.38) | 979.78 (948.66, 1012.22) | -17.03 (-20.00, -13.95) |
| Low SDI^b^ | 14229737.79  (13825034.55, 14600093.94) | 27628145.40  (26751744.78, 28471755.53) | 2446.61 (2384.88, 2501.46) | 2196.37 (2135.89, 2258,17) | -10.23 (-13.12, -7.26) |
| Low-middle SDI | 30709536.09  (29981958.12, 31449487.13) | 39878394.32  (38856843.50, 41063045.61) | 2385.88 (2335.63, 2439.10) | 2049.82 (1999.46, 2107.19) | -14.09 (-16.68, -10.94) |
| Middle SDI | 25173707.86  (24676035.57, 25704352.74) | 25222573.91  (24707661.05, 25742711.96) | 1392.73 (1367.00, 1420.29) | 1103.24 (1078.55, 1127.53) | -20.79 (-22.85, -18.90) |
| High-middle SDI | 10234470.32  (9922288.73, 10565582.00) | 7429881.57  (7165769.29, 7716098.59) | 988.72 (959.00, 1021.48) | 645.20 (619.22, 672.38) | -34.74 (-37.67, -31.71) |
| High SDI | 4110671.30  (3886794.95, 4356143.25) | 3527833.67  (3350846.24, 3722025.10) | 502.14 (474.79, 529.93) | 362.54 (343.40, 383.37) | -27.80 (-33.12, -21.95) |
| Central Europe, Eastern Europe, and Central Asia | 5217327.62  (5036670.59, 5430677.26) | 3983491.51  (3818459.80, 4166298.69) | 1298.45 (1255.30, 1350.90) | 1091.54 (1044.61, 1146.64) | -15.94 (-20.23, -11.00) |
| Central Asia | 1552728.55  (1465278.82, 1652360.47) | 1688745.10  (1592946.63, 1817739.69) | 2026.26 (1918.38, 2148.24) | 1727.06 (1628.31, 1861.03) | -14.77 (-21.92, -6.69) |
| Central Europe | 1482722.82  (1406731.99, 1563485.54) | 832239.36  (789060.24, 886499.57) | 1280.13 (1212.90, 1354.74) | 872.03 (819.69, 940.15) | -31.88 (-37.05, -26.31) |
| Eastern Europe | 2181876.24  (2042422.16, 2334770.77) | 1462507.05  (1354146.03, 1587484.83) | 1018.18 (959.42, 1087.62) | 786.20 (723.91, 858.84) | -22.78 (-30.12, -15.36) |
| High-income | 3460485.72  (3218254.05, 3741419.92) | 2965545.82  (2763489.68, 3186718.23) | 416.77 (387.21, 450.91) | 310.2 (286.17, 339.52) | -25.57 (-33.11, -16.54) |
| Australasia | 77053.43  (61587.05, 113484.99) | 80384.21  (67424.89, 110552.32) | 417.53 (319.84, 650.59) | 301.36 (238.45, 460.81) | -27.82 (-56.73, 19.09) |
| High-income Asia Pacific | 788293.09  (720586.86, 868554.71) | 942285.16  (857141.45, 1065707.71) | 283.76 (260.49, 311.45) | 269.55 (242.47, 304.52) | -5.01 (-17.62, 9.87) |
| High-income North America | 844679.11  (715597.91, 998468.87) | 582886.85  (502835.58, 688862.87) | 517.85 (435.96, 621.92) | 341.18 (285.24, 408.77) | -34.12 (-48.42, -12.59) |
| Southern Latin America | 390085.90  (290925.96, 541717.25) | 319835.48  (213310.41, 483046.61) | 780.11 (586.89, 1075.86) | 559.48 (366.65, 860.43) | -28.28 (-57.77, 18.68) |
| Western Europe | 1360374.19  (1232359.05, 1541499.26) | 1040154.12  (963895.21, 1156707.63) | 413.76 (373.74, 465.84) | 288.29 (264.78, 321.4) | -30.32 (-40.00, -19.37) |
| Latin America and Caribbean | 5323587.77  (5041205.25, 5635670.79) | 5353725.69  (5081875.13, 5704446.76) | 1245.60 (1182.57, 1315.99) | 951.22 (902.39, 1013.46) | -23.63 (-28.98, -17.44) |
| Andean Latin America | 652649.07  (584712.13, 748254.05) | 595268.41  (537620.73, 691497.61) | 1497.76 (1351.83, 1693.54) | 912.52 (825.53, 1064.72) | -39.07 (-48.22, -25.73) |
| Caribbean | 617307.61  (587068.63, 653397.78) | 756547.31  (711289.62, 809762.31) | 1653.04 (1574.11, 1741.01) | 1702.45 (1595.7, 1825.51) | 2.99 (-5.21, 12.88) |
| Central Latin America | 1518046.03  (1448605.73, 1594748.07) | 1427310.82  (1379784.67, 1493948.96) | 799.77 (767.36, 835.85) | 602.13 (582.27, 629.41) | -24.71 (-28.76, -20.46) |
| Tropical Latin America | 2535585.06  (2297139.54, 2802817.41) | 2574599.16  (2329408.59, 2886456.25) | 1564.24 (1423.70, 1725.63) | 1201.39 (1087.70, 1361.69) | -23.2 (-32.86, -10.65) |
| North Africa and Middle East | 6082999.67  (5837788.46, 6375590.8) | 7441711.10  (7182492.55, 7746681.53) | 1575.70 (1517.27, 1646.35) | 1177.34 (1136.95, 1224.37) | -25.28 (-28.80, -22.05) |
| North Africa and Middle East | 6082999.67  (5837788.46, 6375590.8) | 7441711.10  (7182492.55, 7746681.53) | 1575.70 (1517.27, 1646.35) | 1177.34 (1136.95, 1224.37) | -25.28 (-28.80, -22.05) |
| South Asia | 34264704.93  (33412748.04, 35089389.54) | 45892623.95  (44755328.93, 47170863.57) | 2856.28 (2794.40, 2914.03) | 2532.10 (2470.01, 2599.37) | -11.35 (-14.30, -8.28) |
| South Asia | 34264704.93  (33412748.04, 35089389.54) | 45892623.95  (44755328.93, 47170863.57) | 2856.28 (2794.40, 2914.03) | 2532.10 (2470.01, 2599.37) | -11.35 (-14.30, -8.28) |
| Southeast Asia, East Asia, and Oceania | 18647705.09  (18145676.99, 19190722.87) | 13583034.65  (13115862.23, 14016121.92) | 1091.46 (1063.14, 1121.62) | 680.01 (656.81, 701.48) | -37.70 (-39.75, -35.54) |
| East Asia | 10857354.29  (10519352.96, 11196240.47) | 5649957.63  (5435305.18, 5848650.96) | 894.20 (867.72, 922.17) | 403.30 (387.56, 417.54) | -54.90 (-56.12, -53.62) |
| Oceania | 134557.36  (120791.8, 153818.79) | 265559.26  (228888.30, 311763.76) | 1869.45 (1702.96, 2094.97) | 1741.66 (1517.64, 2024.04) | -6.84 (-21.82, 11.23) |
| Southeast Asia | 7655793.44  (7320202.83, 8031219.98) | 7667517.77  (7327185.55, 7987321.45) | 1549.51 (1485.71, 1616.40) | 1137.66 (1088.78, 1184.80) | -26.58 (-30.44, -21.99) |
| Sub-Saharan Africa | 11533474.59  (11171186.14, 11884209.07) | 24542309.57  (23693825.88, 25457725.18) | 1963.69 (1903.92, 2018.21) | 1861.76 (1804.50, 1921.92) | -5.19 (-8.94, -1.28) |
| Central sub-Saharan Africa | 1250898.99  (1136676.26, 1388273.59) | 2227573.80  (1999207.85, 2499991.14) | 2005.76 (1843.53, 2183.05) | 1461.20 (1328.63, 1627.11) | -27.15 (-35.02, -17.08) |
| Eastern sub-Saharan Africa | 4547975.53  (4353631.17, 4727882.69) | 8318792.45  (7890955.89, 8731611.71) | 1950.64 (1879.62, 2017.45) | 1686.87 (1614.07, 1759.51) | -13.52 (-17.95, -8.78) |
| Southern sub-Saharan Africa | 1001066.05  (941119.83, 1066956.90) | 1254300.92  (1180643.47, 1343008.78) | 1689.27 (1597.60, 1799.63) | 1520.05 (1434.01, 1623.41) | -10.02 (-16.93, -1.73) |
| Western sub-Saharan Africa | 4733534.02  (4467401.86, 5003409.40) | 12741642.4  (12106877.64, 13462583.00) | 2042.77 (1939.61, 2156.37) | 2193.41 (2101.89, 2297.78) | 7.37 (-0.22, 14.43) |

Abbreviation: GBD: Global Burden of Disease; SDI, socio-demographic index; rIDPs, rare infectious diseases of poverty; UI: uncertainty interval.

**a** percentage change in age-standardized rates between 1990 and 2021; **b** SDI is an indicator of a country’s development level and is comprised of the lag-dependent income per capita, the gross domestic product per capita smoothed over the previous 10 years, education level among the population aged ≥15 years old and the total fertility rate <25 years old.

# Table S5. Age-standardized mortality and YLL rates (per 100,000 population) of rIDPs and the percentage changes in the age-standardized rates, by sex, SDI levels, GBD super regions and sub-regions, from 1990 to 2021

| **Location** | **Mortality^a^** | | | **YLLs^b^** | | |
| --- | --- | --- | --- | --- | --- | --- |
|  | **1990**  **(95% UI)** | **2021**  **(95% UI)** | **change, %^c^**  **(95% UI)** | **1990**  **(95% UI)** | **2021**  **(95% UI)** | **change, %^c^**  **(95% UI)** |
| Global | 0.26 (0.21, 0.33) | 0.30 (0.21, 0.38) | 17.49 (-27.39, 52.87) | 16.24 (12.30, 22.50) | 20.50 (13.04, 26.56) | 26.23 (-31.97, 76.61) |
| Female | 0.21 (0.18, 0.28) | 0.25 (0.18, 0.32) | 17.08 (-29.14, 59.68) | 14.05 (10.68, 19.82) | 17.14 (11.54, 22.80) | 22.03 (-35.02, 76.58) |
| Male | 0.31 (0.25, 0.39) | 0.36 (0.23, 0.45) | 16.18 (-26.28, 52.24) | 18.43 (13.27, 25.43) | 23.73 (14.34, 31.27) | 28.75 (-30.15, 81.58) |
| Low SDI^d^ | 0.99 (0.73, 1.36) | 0.96 (0.95, 1.24) | -2.60 (-43.89, 31.44) | 55.29 (36.84, 87.64) | 56.21 (36.60, 73.05) | 1.66 (-50.12, 50.82) |
| Low-middle SDI | 0.34 (0.29, 0.44) | 0.36 (0.25, 0.44) | 8.30 (-27.39, 37.46) | 18.06 (14.86, 22.04) | 20.20 (12.39, 26.27) | 11.85 (-37.01, 50.90) |
| Middle SDI | 0.22 (0.20, 0.25) | 0.16 (0.11, 0.20) | -28.74 (-45.81, -11.7) | 11.87 (9.91, 13.28) | 8.53 (5.44, 11.30) | -28.12 (-53.63, -4.78) |
| High-middle SDI | 0.09 (0.07, 0.11) | 0.05 (0.05, 0.06) | -42.79 (-51.10, -33.53) | 5.12 (3.69, 5.77) | 2.39 (1.87, 2.78) | -53.31 (-62.67, -42.44) |
| High SDI | 0.04 (0.03, 0.04) | 0.04 (0.03, 0.04) | 9.26 (-0.15, 15.76) | 1.45 (1.33, 1.55) | 1.42 (1.24, 1.54) | -1.52 (-10.51, 6.90) |
| Central Europe, Eastern Europe, and Central Asia | 0.06 (0.06, 0.07) | 0.05 (0.05, 0.06) | -15.75 (-24.62, -6.19) | 3.69 (3.35, 3.96) | 2.94 (2.44, 3.42) | -20.35 (-33.25, -6.66) |
| Central Asia | 0.06 (0.05, 0.07) | 0.09 (0.07, 0.11) | 51.86 (13.73, 97.18) | 3.64 (2.88, 4.67) | 4.68 (3.56, 5.98) | 28.49 (-11.85, 77.52) |
| Central Europe | 0.05 (0.05, 0.06) | 0.02 (0.02, 0.03) | -59.94 (-65.57, -53.85) | 2.03 (1.69, 2.32) | 0.72 (0.60, 0.87) | -64.42 (-70.83, -56.36) |
| Eastern Europe | 0.07 (0.06, 0.08) | 0.06 (0.05, 0.06) | -23.67 (-31.15, -14.74) | 4.73 (4.38, 5.13) | 3.10 (2.71, 3.43) | -34.53 (-42.45, -25.74) |
| High-income | 0.04 (0.03, 0.04) | 0.04 (0.03, 0.04) | 8.31 (-2.21, 14.60) | 1.39 (1.31, 1.44) | 1.43 (1.25, 1.56) | 3.02 (-7.23, 10.71) |
| Australasia | 0.02 (0.02, 0.03) | 0.01 (0.01, 0.01) | -44.71 (-54.39, -34.61) | 0.81 (0.72, 0.9) | 0.38 (0.30, 0.45) | -53.81 (-63.26, -43.47) |
| High-income Asia Pacific | 0.03 (0.03, 0.03) | 0.07 (0.06, 0.07) | 114.78 (101.26, 127.76) | 1.32 (1.23, 1.38) | 2.57 (2.29, 2.79) | 94.77 (79.64, 111.32) |
| High-income North America | 0.03 (0.03, 0.03) | 0.03 (0.01, 0.03) | -14.02 (-56.44, 0.97) | 1.09 (1.01, 1.27) | 0.74 (0.45, 0.86) | -31.93 (-60.13, -20.85) |
| Southern Latin America | 0.04 (0.04, 0.05) | 0.05 (0.04, 0.06) | 15.99 (1.16, 35.19) | 2.01 (1.81, 2.19) | 2.08 (1.73, 2.46) | 3.65 (-12.33, 23.36) |
| Western Europe | 0.04 (0.04, 0.04) | 0.02 (0.02, 0.02) | -46.12 (-50.38, -41.83) | 1.48 (1.39, 1.56) | 0.64 (0.56, 0.72) | -56.51 (-60.64, -52.19) |
| Latin America and Caribbean | 0.48 (0.46, 0.50) | 0.18 (0.16, 0.21) | -62.41 (-67.73, -56.89) | 30.54 (28.85, 32.51) | 9.16 (7.39, 11.06) | -70.02 (-76.24, -63.36) |
| Andean Latin America | 0.32 (0.26, 0.38) | 0.13 (0.10, 0.18) | -58.8 (-68.73, -44.79) | 17.60 (13.67, 21.67) | 6.12 (4.24, 8.65) | -65.24 (-75.78, -47.92) |
| Caribbean | 0.08 (0.07, 0.10) | 0.10 (0.08, 0.12) | 19.18 (-5.00, 46.39) | 4.57 (3.30, 5.39) | 4.36 (3.33, 5.51) | -4.60 (-28.81, 25.89) |
| Central Latin America | 0.73 (0.68, 0.77) | 0.16 (0.13, 0.20) | -77.37 (-81.20, -72.54) | 45.38 (41.9, 48.76) | 9.05 (6.66, 11.76) | -80.05 (-84.94, -74.13) |
| Tropical Latin America | 0.35 (0.31, 0.39) | 0.23 (0.20, 0.25) | -34.59 (-46.71, -24.56) | 22.49 (19.42, 27.21) | 11.23 (9.64, 12.89) | -50.08 (-63.09, -37.85) |
| North Africa and Middle East | 0.10 (0.08, 0.12) | 0.07 (0.05, 0.08) | -29.56 (-46.70, -10.51) | 4.70 (3.04, 5.80) | 3.37 (1.56, 4.29) | -28.40 (-54.83, -1.38) |
| North Africa and Middle East | 0.10 (0.08, 0.12) | 0.07 (0.05, 0.08) | -29.56 (-46.70, -10.51) | 4.70 (3.04, 5.80) | 3.37 (1.56, 4.29) | -28.40 (-54.83, -1.38) |
| South Asia | 0.17 (0.14, 0.24) | 0.19 (0.14, 0.24) | 7.62 (-27.99, 42.76) | 6.87 (5.28, 9.50) | 7.10 (5.47, 9.22) | 3.32 (-29.57, 42.09) |
| South Asia | 0.17 (0.14, 0.24) | 0.19 (0.14, 0.24) | 7.62 (-27.99, 42.76) | 6.87 (5.28, 9.50) | 7.10 (5.47, 9.22) | 3.32 (-29.57, 42.09) |
| Southeast Asia, East Asia, and Oceania | 0.14 (0.11, 0.16) | 0.10 (0.06, 0.11) | -33.17 (-48.15, -18.69) | 7.26 (4.57, 9.04) | 4.67 (2.59, 5.71) | -35.73 (-56.61, -17.81) |
| East Asia | 0.10 (0.09, 0.13) | 0.05 (0.04, 0.06) | -52.55 (-63.60, -41.23) | 5.87 (4.33, 7.06) | 2.23 (1.61, 2.81) | -61.99 (-72.55, -49.97) |
| Oceania | 0.46 (0.32, 0.81) | 0.42 (0.29, 0.59) | -8.13 (-40.41, 22.54) | 8.03 (6.06, 12.06) | 7.05 (4.86, 9.67) | -12.25 (-36.59, 14.40) |
| Southeast Asia | 0.25 (0.15, 0.30) | 0.21 (0.12, 0.25) | -16.03 (-34.00, 2.41) | 10.88 (4.88, 16.36) | 9.11 (4.22, 11.45) | -16.20 (-47.67, 10.86) |
| Sub-Saharan Africa | 1.41 (1.07, 1.97) | 1.31 (0.89, 1.70) | -6.59 (-43.47, 24.67) | 78.02 (54.15, 114.72) | 74.87 (47.09, 99.39) | -4.04 (-51.85, 39.61) |
| Central sub-Saharan Africa | 0.66 (0.19, 0.98) | 0.60 (0.16, 0.98) | -8.92 (-41.42, 25.87) | 25.21 (5.88, 43.01) | 21.14 (5.12, 32.48) | -16.14 (-54.91, 24.32) |
| Eastern sub-Saharan Africa | 0.67 (0.20, 0.91) | 0.58 (0.16, 0.91) | -13.95 (-44.08, 10.32) | 26.35 (6.54, 43.84) | 21.98 (5.97, 33.73) | -16.59 (-55.99, 16.78) |
| Southern sub-Saharan Africa | 0.34 (0.26, 0.42) | 0.36 (0.25, 0.46) | 5.60 (-13.98, 32.37) | 16.78 (11.91, 20.82) | 18.32 (11.27, 24.52) | 9.15 (-22.22, 49.21) |
| Western sub-Saharan Africa | 2.60 (2.03, 4.40) | 2.28 (1.53, 3.03) | -12.26 (-47.64, 22.82) | 158.54 (115.95, 234.73) | 139.44 (85.35, 188.10) | -12.05 (-56.73, 31.01) |

Abbreviation: GBD: global burden of disease; SDI, socio-demographic index; rIDPs, rare infectious diseases of poverty; UI: uncertainty interval; YLLs, years of life lost.

**a** age-standardized mortality rates per 100,000 population; **b** age-standardized YLL rates per 100,000 population; **c** percentage change in age-standardized rates between 1990 and 2021; **d** SDI is an indicator of a country’s development level and is comprised of the lag-dependent income per capita, the gross domestic product per capita smoothed over the previous 10 years, education level among the population aged ≥15 years old and the total fertility rate <25 years old.

# Table S6. Number of mortality and DALYs of rIDPs by sex, SDI levels, GBD super regions and sub-regions, from 1990 to 2021

| **Location** | **Mortality** | | **DALYs** | |
| --- | --- | --- | --- | --- |
|  | **Number, 1990**  **(95% UI)** | **Number, 2021**  **(95% UI)** | **Number, 1990**  **(95% UI)** | **Number, 2021**  **(95% UI)** |
| Global | 13642.74 (10702.89, 17837.21) | 21942.91 (15207.29, 26891.43) | 3528751.63 (2619635.44, 4663679.76) | 4218302.25 (3095236.34, 5602012.77) |
| Female | 5744.11 (4628.63, 7673.79) | 9092.08 (6701.98, 11401.57) | 1987388.26 (1440079.06, 2691735.58) | 2505328.34 (1807484.04, 3391379.14) |
| Male | 7898.63 (5962.63, 10489.79) | 12850.82 (8501.47, 16143.21) | 1541363.37 (1172881.60, 2005697.64) | 1712973.90 (1220730.04, 2219097.85) |
| Low SDI^d^ | 5456.85 (3498.99, 8802.46) | 10693.12 (6961.92, 13896.99) | 906032.12 (670661.96, 1252447.86) | 1635820.54 (1194415.59, 2105333.65) |
| Low-middle SDI | 3692.50 (3080.01, 4535.99) | 6093.08 (4072.00, 7522.75) | 1307848.29 (971095.27, 1761876.62) | 1513313.79 (1101763.99, 2064325.40) |
| Middle SDI | 3237.28 (2774.35, 3538.78) | 3594.74 (2594.92, 4299.67) | 936206.84 (704383.81, 1249414.52) | 813153.93 (590424.42, 1115710.92) |
| High-middle SDI | 912.20 (693.09, 1018.86) | 849.92 (648.05, 986.08) | 297051.07 (216307.38, 410696.43) | 180901.03 (129393.29, 248688.56) |
| High SDI | 339.05 (307.01, 359.60) | 705.05 (593.71, 771.80) | 79538.33 (55921.38, 114432.46) | 73055.60 (53753.73, 100747.32) |
| Central Europe, Eastern Europe, and Central Asia | 249.29 (218.52, 268.33) | 246.32 (216.35, 272.95) | 150240.80 (105889.22, 212646.01) | 105697.21 (74751.63, 149511.67) |
| Central Asia | 42.99 (34.48, 54.16) | 82.37 (65.34, 102.21) | 52642.45 (36150.36, 75501.24) | 52288.96 (37094.58, 74046.68) |
| Central Europe | 59.67 (51.37, 65.74) | 41.70 (35.94, 48.42) | 34943.90 (23401.33, 50394.00) | 15934.89 (10816.47, 22922.84) |
| Eastern Europe | 146.63 (125.04, 161.85) | 122.25 (109.55, 132.99) | 62654.45 (44416.93, 90407.50) | 37473.36 (26968.45, 53204.13) |
| High-income | 376.92 (351.57, 394.81) | 746.55 (634.46, 816.17) | 59320.43 (41674.72, 85226.70) | 59371.89 (44217.64, 81205.13) |
| Australasia | 4.69 (4.17, 5.21) | 6.65 (5.46, 7.79) | 1105.33 (707.65, 1683.36) | 1197.60 (771.48, 1875.11) |
| High-income Asia Pacific | 98.46 (90.42, 104.21) | 371.65 (333.60, 402.89) | 13913.92 (10080.13, 19642.23) | 24291.89 (19118.35, 31629.29) |
| High-income North America | 50.64 (46.47, 55.10) | 123.19 (60.59, 149.99) | 10382.37 (6659.20, 16133.28) | 8895.39 (6087.23, 12846.61) |
| Southern Latin America | 20.75 (18.90, 22.61) | 39.54 (34.07, 45.32) | 5667.45 (3481.38, 9443.96) | 4826.92 (3233.10, 7566.72) |
| Western Europe | 202.37 (186.85, 214.52) | 205.53 (176.58, 227.98) | 28251.36 (20077.87, 39279.74) | 20160.10 (14447.8, 28264.33) |
| Latin America and Caribbean | 1871.54 (1774.25, 1978.25) | 1034.49 (901.08, 1167.54) | 289537.70 (239330.01, 353804.30) | 183896.91 (137402.21, 243089.88) |
| Andean Latin America | 115.29 (91.08, 141.03) | 81.36 (59.64, 109.64) | 24580.22 (18119.44, 32877.43) | 16531.42 (11710.81, 23156.22) |
| Caribbean | 27.67 (23.40, 31.45) | 50.80 (40.86, 61.14) | 19483.04 (13136.77, 27287.67) | 20873.67 (14015.86, 29347.08) |
| Central Latin America | 1244.32 (1149.72, 1333.69) | 382.66 (315.97, 454.81) | 130620.17 (115763.09, 150183.26) | 51730.66 (39672, 67775.68) |
| Tropical Latin America | 484.26 (427.26, 567.08) | 519.67 (469.82, 564.87) | 114854.27 (87348.17, 149039.95) | 94761.16 (70177.35, 127626.35) |
| North Africa and Middle East | 279.62 (187.90, 340.57) | 361.86 (210.92, 445.05) | 196605.11 (135400.08, 272628.87) | 221439.82 (156653.72, 311003.35) |
| North Africa and Middle East | 279.62 (187.90, 340.57) | 361.86 (210.92, 445.05) | 196605.11 (135400.08, 272628.87) | 221439.82 (156653.72, 311003.35) |
| South Asia | 1343.60 (1038.01, 1906.28) | 2817.47 (2140.78, 3652.20) | 1292276.45 (907087.75, 1794508.26) | 1442285.63 (1018059.25, 2034658.58) |
| South Asia | 1343.60 (1038.01, 1906.28) | 2817.47 (2140.78, 3652.20) | 1292276.45 (907087.75, 1794508.26) | 1442285.63 (1018059.25, 2034658.58) |
| Southeast Asia, East Asia, and Oceania | 1962.48 (1315.89, 2267.74) | 2161.69 (1329.32, 2552.15) | 585854.04 (428363.76, 801436.39) | 396790.55 (293601.55, 538903.28) |
| East Asia | 1086.39 (887.16, 1302.45) | 859.54 (645.02, 1078.74) | 340177.54 (248176.17, 460215.33) | 155565.43 (112376.92, 213837.99) |
| Oceania | 8.26 (6.24, 12.11) | 19.47 (13.26, 26.84) | 3162.01 (2027.30, 4530.58) | 6120.77 (3961.43, 9697.25) |
| Southeast Asia | 867.83 (420.53, 1218.65) | 1282.68 (666.94, 1565.22) | 242514.49 (170154.94, 335369.07) | 235104.36 (171115.04, 319601.61) |
| Sub-Saharan Africa | 7559.3 (5093.13, 11332.11) | 14574.53 (9149.11, 19360.17) | 954917.10 (708439.77, 1321850.54) | 1808820.24 (1275272.49, 2332600.69) |
| Central sub-Saharan Africa | 251.53 (54.44, 473.83) | 468.27 (111.73, 718.13) | 52219.34 (34462.78, 75306.81) | 80172.80 (53436.51, 113518.58) |
| Eastern sub-Saharan Africa | 916.26 (215.23, 1683.91) | 1540.17 (420.70, 2361.67) | 211611.43 (144156.59, 304297.90) | 316515.16 (219403.11, 440734.56) |
| Southern sub-Saharan Africa | 153.38 (110.59, 190.42) | 246.05 (162.09, 317.42) | 45258.74 (33808.15, 61035.98) | 56398.83 (40592.30, 73957.34) |
| Western sub-Saharan Africa | 6238.12 (4454.66, 9244.45) | 12320.03 (7477.43, 16693.18) | 645827.58 (481050.46, 907313.44) | 1355733.44 (922780.18, 1768850.76) |

Abbreviation: DALYs, disability-adjusted life years; GBD: global burden of disease; SDI, socio-demographic index; rIDPs, rare infectious diseases of poverty; UI: uncertainty interval.

**a** SDI is an indicator of a country’s development level and is comprised of the lag-dependent income per capita, the gross domestic product per capita smoothed over the previous 10 years, education level among the population aged ≥15 years old and the total fertility rate <25 years old.

# Table S7. Number of YLLs and YLDs of rIDPs by sex, SDI levels, GBD super regions and sub-regions, from 1990 to 2021

| **Location** | **YLLs** | | **YLDs** | |
| --- | --- | --- | --- | --- |
|  | **Number, 1990**  **(95% UI)** | **Number, 2021**  **(95% UI)** | **Number, 1990**  **(95% UI)** | **Number, 2021**  **(95% UI)** |
| Global | 948322.46 (709023.99, 1327991.44) | 1404589.30 (905698.81,1803424.61) | 2580429.16 (1753582.98, 3648312.63) | 2813712.94 (1890225.02, 4064281.16) |
| Female | 404065.01 (301772.53, 577715.09) | 572409.92 (392177.13, 749354.26) | 1583323.25 (1079416.56, 2236246.56) | 1932918.42 (1302203.48, 2771490.44) |
| Male | 544257.46 (382493.34, 767595.87) | 832179.38 (510465.65, 1086979.86) | 997105.91 (673909.02, 1412522.10) | 880794.52 (586559.35, 1285112.49) |
| Low SDI^a^ | 415558.49 (246568.43, 712691.23) | 804868.83 (509964.24,1047630.33) | 490473.63 (331047.12, 685759.68) | 830951.72 (552837.33, 1197359.10) |
| Low-middle SDI | 259119.10 (202854.69, 325171.22) | 374706.53 (226434.13,489433.63) | 1048729.19 (711846.65, 1488013.33) | 1138607.26 (759769.65, 1635684.80) |
| Middle SDI | 211748.26 (171425.31, 239810.35) | 177497.41 (115188,228038.75) | 724458.58 (491166.62, 1033563.17) | 635656.53 (424127.09, 920793.19) |
| High-middle SDI | 49707.47 (35619.08, 55738.20) | 29749.42 (22696.85, 34411.23) | 247343.60 (163356.21, 360397.24) | 151151.61 (100972.08, 219488.26) |
| High SDI | 11931.44 (10912.54, 12717.03) | 17468.88 (15048.76, 18881.98) | 67606.89 (43830.19, 102342.29) | 55586.71 (36405.15, 83151.33) |
| Central Europe, Eastern Europe, and Central Asia | 13785.36 (12373.13, 14832.83) | 10726.14 (9155.95, 12216.06) | 136455.44 (91447.50, 198896.02) | 94971.07 (63791.41, 138477.22) |
| Central Asia | 2934.75 (2289.80, 3884.76) | 4502.54 (3401.70, 5769.60) | 49707.70 (33395.97, 72392.18) | 47786.42 (32447.67, 69590.94) |
| Central Europe | 2236.72 (1875.97, 2526.78) | 1041.36 (870.13, 1232.52) | 32707.18 (20994.38, 48010.76) | 14893.53 (9696.93, 21889.15) |
| Eastern Europe | 8613.89 (7770.12, 9422.09) | 5182.24 (4615.15, 5679.29) | 54040.56 (35507.07, 81611.14) | 32291.12 (21860.23, 48188.27) |
| High-income | 12291.25 (11585.68, 12759.42) | 17879.48 (15614.35, 19276.6) | 47029.18 (29298.53, 73253.14) | 41492.41 (26219.1, 63181.35) |
| Australasia | 152.45 (136.82, 169.47) | 135.66 (111.48, 160.92) | 952.88 (563.81, 1531.50) | 1061.94 (640.80, 1741.80) |
| High-income Asia Pacific | 3572.78 (3309.96, 3747.50) | 10148.14 (9156.22, 10931.66) | 10341.14 (6460.19, 15990.61) | 14143.75 (8721.26, 21597.18) |
| High-income North America | 1728.05 (1593.92, 2023.39) | 2261.78 (1141.01, 2711.46) | 8654.32 (4917.56, 14401.18) | 6633.61 (3982.92, 10412.89) |
| Southern Latin America | 982.70 (885.10, 1073.08) | 1343.95 (1133.54, 1553.93) | 4684.76 (2507.26, 8467.04) | 3482.97 (1877.54, 6196.22) |
| Western Europe | 5855.28 (5493.98, 6173.73) | 3989.95 (3498.58, 4392.66) | 22396.07 (14321.87, 33347.08) | 16170.15 (10293.78, 24239.49) |
| Latin America and Caribbean | 138952.72 (130715.98, 148596.76) | 49641.68 (40883.53, 58948.05) | 150584.98 (99900.64, 216221.12) | 134255.23 (87472.93, 195327.73) |
| Andean Latin America | 7844.25 (5956.85, 9731.39) | 3865.87 (2676.41, 5453.24) | 16735.96 (10718.66, 24379.11) | 12665.56 (8170.37, 18824.72) |
| Caribbean | 1734.68 (1162.17, 2075.06) | 1973.27 (1531.68, 2463.40) | 17748.36 (11439.15, 25664.08) | 18900.41 (12151.83, 27451.05) |
| Central Latin America | 94096.82 (86278.93, 101716.66) | 20230.63 (15313.83, 25744.23) | 36523.36 (24231.14, 53299.18) | 31500.03 (20442.39, 46666.79) |
| Tropical Latin America | 35276.97 (30304.50, 43021.7) | 23571.92 (20637.01, 26442.89) | 79577.3 (52922.60, 115733.74) | 71189.24 (46545.25, 105257.64) |
| North Africa and Middle East | 17958.07 (10438.51, 24170.06) | 19728.62 (8487.92, 25296.33) | 178647.04 (116444.80, 254382.89) | 201711.20 (135463.20, 292003.64) |
| North Africa and Middle East | 17958.07 (10438.51, 24170.06) | 19728.62 (8487.92, 25296.33) | 178647.04 (116444.80, 254382.89) | 201711.20 (135463.20, 292003.64) |
| South Asia | 72469.52 (54212.88, 105059.37) | 120272.99 (92766.81, 155580.86) | 1219806.93 (832550.29, 1728715.04) | 1322012.64 (887510.66, 1913027.26) |
| South Asia | 72469.52 (54212.88, 105059.37) | 120272.99 (92766.81, 155580.86) | 1219806.93 (832550.29, 1728715.04) | 1322012.64 (887510.66, 1913027.26) |
| Southeast Asia, East Asia, and Oceania | 117574.97 (70949.55, 148806.14) | 89580.40 (50925.68, 107492.09) | 468279.06 (310619.85, 675500.82) | 307210.15 (202005.07, 447608.60) |
| East Asia | 66529.37 (48685.00, 80096.24) | 30890.37 (23077.68, 39024.27) | 273648.17 (181680.57, 395046.74) | 124675.06 (81886.68, 180451.26) |
| Oceania | 252.41 (179.49, 321.49) | 465.89 (312.10, 624.97) | 2909.60 (1762.72, 4298.70) | 5654.88 (3465.02, 9240.97) |
| Southeast Asia | 50793.19 (20020.86, 83778.08) | 58224.14 (26007.62, 72827.51) | 191721.30 (125482.64, 278570.86) | 176880.21 (116655.62, 256909.64) |
| Sub-Saharan Africa | 575290.57 (365835.25, 909518.44) | 1096759.99 (662271.40, 1479339.40) | 379626.53 (257770.77, 537258.05) | 712060.24 (467462.33, 1028074.21) |
| Central sub-Saharan Africa | 16206.89 (2921.79, 36224.45) | 26553.55 (5686.15, 39681.66) | 36012.46 (23502.63, 53080.28) | 53619.25 (34379.42, 80837.33) |
| Eastern sub-Saharan Africa | 59745.60 (12012.08, 132219.31) | 92804.07 (22912.43, 138819.82) | 151865.83 (103122.80, 212412.15) | 223711.09 (149662.21, 329390.43) |
| Southern sub-Saharan Africa | 9897.44 (6776.59, 12362.03) | 14136.87 (8510.12, 19035.05) | 35361.30 (23543.06, 50258.45) | 42261.96 (28495.67, 59204.72) |
| Western sub-Saharan Africa | 489440.64 (320229.58, 751120.18) | 963265.50 (559671.22, 1331495.54) | 156386.94 (106190.73, 226648.19) | 392467.94 (256200.71, 571053.63) |

Abbreviation: GBD: global burden of disease; SDI, socio-demographic index; rIDPs, rare infectious diseases of poverty; UI: uncertainty interval; YLLs, years of life lost; YLDs, years lived with disability.

**a** SDI is an indicator of a country’s development level and is comprised of the lag-dependent income per capita, the gross domestic product per capita smoothed over the previous 10 years, education level among the population aged ≥15 years old and the total fertility rate <25 years old.

# Table S8. Temporal trends in age-standardized prevalence rates of rIDPs by sex, SDI levels, and GBD super regions, from 1990 to 2021.

| **Groups** | **Time periods identified** | | **Temporal trend^a^** | | | | | |
| --- | --- | --- | --- | --- | --- | --- | --- | --- |
|  |  |  | **APC** | | | **AAPC** | | |
|  |  |  | **%** | **95% *CI*** | ***P* value** | **%** | **95% *CI*** | ***P* value** |
| Global | Period1 | 1990-1993 | -0.39 | (-0.54, -0.25) | < 0.001 | -0.28 | (-0.32, -0.25) | < 0.001 |
|  | Period2 | 1993-2002 | -0.18 | (-0.22, -0.15) | < 0.001 |  |  |  |
|  | Period3 | 2002-2006 | -0.37 | (-0.51, -0.22) | < 0.001 |  |  |  |
|  | Period4 | 2006-2009 | -0.69 | (-0.98, -0.41) | < 0.001 |  |  |  |
|  | Period5 | 2009-2021 | -0.20 | (-0.21, -0.18) | < 0.001 |  |  |  |
| Male | Period1 | 1990-1994 | -0.51 | (-0.60, -0.42) | < 0.001 | -0.61 | (-0.65, -0.57) | < 0.001 |
|  | Period2 | 1994-2001 | -0.28 | (-0.33, -0.23) | < 0.001 |  |  |  |
|  | Period3 | 2001-2006 | -0.70 | (-0.80, -0.60) | < 0.001 |  |  |  |
|  | Period4 | 2006-2009 | -1.20 | (-1.51, -0.90) | < 0.001 |  |  |  |
|  | Period5 | 2009-2014 | -0.57 | (-0.67, -0.47) | < 0.001 |  |  |  |
|  | Period6 | 2014-2021 | -0.70 | (-0.75, -0.64) | < 0.001 |  |  |  |
| Female | Period1 | 1990-1993 | -0.29 | (-0.35, -0.22) | < 0.001 | -0.08 | (-0.10, -0.06) | < 0.001 |
|  | Period2 | 1993-2006 | -0.11 | (-0.12, -0.10) | < 0.001 |  |  |  |
|  | Period3 | 2006-2009 | -0.45 | (-0.57, -0.32) | < 0.001 |  |  |  |
|  | Period4 | 2009-2015 | 0.09 | (0.06, 0.11) | < 0.001 |  |  |  |
|  | Period5 | 2015-2018 | 0.00 | (-0.14, 0.13) | 0.967 |  |  |  |
|  | Period6 | 2018-2021 | 0.20 | (0.12, 0.28) | < 0.001 |  |  |  |
| Low SDI | Period1 | 1990-2005 | -0.33 | (-0.34, -0.32) | < 0.001 | -0.34 | (-0.35, -0.33) | < 0.001 |
|  | Period2 | 2005-2010 | -0.96 | (-1.02, -0.90) | < 0.001 |  |  |  |
|  | Period3 | 2010-2021 | -0.08 | (-0.10, -0.07) | < 0.001 |  |  |  |
| Low-middle SDI | Period1 | 1990-2001 | -0.35 | (-0.36, -0.35) | < 0.001 | -0.49 | (-0.50, -0.48) | < 0.001 |
|  | Period2 | 2001-2005 | -0.46 | (-0.49, -0.43) | < 0.001 |  |  |  |
|  | Period3 | 2005-2010 | -0.77 | (-0.79, -0.75) | < 0.001 |  |  |  |
|  | Period4 | 2010-2015 | -0.47 | (-0.49, -0.45) | < 0.001 |  |  |  |
|  | Period5 | 2015-2021 | -0.55 | (-0.56, -0.54) | < 0.001 |  |  |  |
| Middle SDI | Period1 | 1990-2001 | -0.69 | (-0.70, -0.67) | < 0.001 | -0.75 | (-0.78, -0.71) | < 0.001 |
|  | Period2 | 2001-2006 | -0.89 | (-0.95, -0.82) | < 0.001 |  |  |  |
|  | Period3 | 2006-2009 | -1.02 | (-1.23, -0.82) | < 0.001 |  |  |  |
|  | Period4 | 2009-2015 | -0.54 | (-0.58, -0.49) | < 0.001 |  |  |  |
|  | Period5 | 2015-2018 | -1.06 | (-1.29, -0.83) | < 0.001 |  |  |  |
|  | Period6 | 2018-2021 | -0.54 | (-0.67, -0.42) | < 0.001 |  |  |  |
| High-middle SDI | Period1 | 1990-2000 | -1.02 | (-1.04, -1.00) | < 0.001 | -1.36 | (-1.40, -1.32) | < 0.001 |
|  | Period2 | 2000-2003 | -1.83 | (-2.07, -1.59) | < 0.001 |  |  |  |
|  | Period3 | 2003-2009 | -2.09 | (-2.14, -2.03) | < 0.001 |  |  |  |
|  | Period4 | 2009-2015 | -1.28 | (-1.34, -1.22) | < 0.001 |  |  |  |
|  | Period5 | 2015-2018 | -1.48 | (-1.76, -1.20) | < 0.001 |  |  |  |
|  | Period6 | 2018-2021 | -0.61 | (-0.77, -0.45) | < 0.001 |  |  |  |
| High SDI | Period1 | 1990-1993 | -2.70 | (-2.96, -2.43) | < 0.001 | -1.04 | (-1.11, -0.97) | < 0.001 |
|  | Period2 | 1993-1999 | -2.23 | (-2.34, -2.12) | < 0.001 |  |  |  |
|  | Period3 | 1999-2002 | -1.41 | (-1.89, -0.93) | < 0.001 |  |  |  |
|  | Period4 | 2002-2005 | -0.99 | (-1.46, -0.52) | < 0.001 |  |  |  |
|  | Period5 | 2005-2012 | 0.01 | (-0.07, 0.09) | 0.779 |  |  |  |
|  | Period6 | 2012-2021 | -0.39 | (-0.44, -0.35) | < 0.001 |  |  |  |
| Central Europe, Eastern Europe, and Central Asia | Period1 | 1990-1994 | -0.21 | (-0.29, -0.13) | < 0.001 | -0.56 | (-0.59, -0.526) | < 0.001 |
|  | Period2 | 1994-2000 | 0.03 | (-0.03, 0.08) | 0.362 |  |  |  |
|  | Period3 | 2000-2003 | -0.90 | (-1.17, -0.63) | < 0.001 |  |  |  |
|  | Period4 | 2003-2011 | -1.28 | (-1.32, -1.25) | < 0.001 |  |  |  |
|  | Period5 | 2011-2018 | -0.63 | (-0.68, -0.58) | < 0.001 |  |  |  |
|  | Period6 | 2018-2021 | 0.24 | (0.08, 0.40) | 0.005 |  |  |  |
| High income | Period1 | 1990-1993 | -2.93 | (-3.07, -2.78) | < 0.001 | -0.95 | (-0.984, -0.913) | < 0.001 |
|  | Period2 | 1993-1999 | -2.29 | (-2.35, -2.23) | < 0.001 |  |  |  |
|  | Period3 | 1999-2003 | -1.36 | (-1.5, -1.23) | < 0.001 |  |  |  |
|  | Period4 | 2003-2006 | -0.69 | (-0.95, -0.43) | < 0.001 |  |  |  |
|  | Period5 | 2006-2014 | 0.27 | (0.23, 0.30) | < 0.001 |  |  |  |
|  | Period6 | 2014-2021 | -0.18 | (-0.23, -0.14) | < 0.001 |  |  |  |
| Latin America and Caribbean | Period1 | 1990-1995 | -1.42 | (-1.45, -1.39) | < 0.001 | -0.87 | (-0.881, -0.855) | < 0.001 |
|  | Period2 | 1995-2000 | -1.54 | (-1.58, -1.51) | < 0.001 |  |  |  |
|  | Period3 | 2000-2006 | -0.61 | (-0.64, -0.59) | < 0.001 |  |  |  |
|  | Period4 | 2006-2010 | -0.96 | (-1.02, -0.91) | < 0.001 |  |  |  |
|  | Period5 | 2010-2018 | -0.50 | (-0.51, -0.48) | < 0.001 |  |  |  |
|  | Period6 | 2018-2021 | -0.19 | (-0.26, -0.12) | < 0.001 |  |  |  |
| South Asia | Period1 | 1990-1997 | -0.21 | (-0.23, -0.20) | < 0.001 | -0.39 | (-0.404, -0.377) | < 0.001 |
|  | Period2 | 1997-2001 | -0.28 | (-0.34, -0.22) | < 0.001 |  |  |  |
|  | Period3 | 2001-2006 | -0.48 | (-0.51, -0.44) | < 0.001 |  |  |  |
|  | Period4 | 2006-2009 | -0.59 | (-0.70, -0.48) | < 0.001 |  |  |  |
|  | Period5 | 2009-2021 | -0.45 | (-0.45, -0.44) | < 0.001 |  |  |  |
| North Africa and Middle East | Period1 | 1990-1999 | -1.15 | (-1.19, -1.10) | < 0.001 | -0.92 | (-0.950, -0.881) | < 0.001 |
|  | Period2 | 1999-2005 | -0.75 | (-0.84, -0.66) | < 0.001 |  |  |  |
|  | Period3 | 2005-2010 | -1.04 | (-1.16, -0.92) | < 0.001 |  |  |  |
|  | Period4 | 2010-2015 | -0.53 | (-0.65, -0.42) | < 0.001 |  |  |  |
|  | Period5 | 2015-2021 | -0.95 | (-1.02, -0.88) | < 0.001 |  |  |  |
| South-East Asia, East Asia and Oceania | Period1 | 1990-1997 | -1.10 | (-1.16, -1.04) | < 0.001 | -1.51 | (-1.550, -1.470) | < 0.001 |
|  | Period2 | 1997-2001 | -1.41 | (-1.60, -1.21) | < 0.001 |  |  |  |
|  | Period3 | 2001-2009 | -2.50 | (-2.56, -2.45) | < 0.001 |  |  |  |
|  | Period4 | 2009-2018 | -1.30 | (-1.35, -1.26) | < 0.001 |  |  |  |
|  | Period5 | 2018-2021 | -0.56 | (-0.83, -0.29) | < 0.001 |  |  |  |
| Sub-Saharan Africa | Period1 | 1990-1993 | -0.36 | (-0.42, -0.30) | < 0.001 | -0.17 | (-0.186, -0.162) | < 0.001 |
|  | Period2 | 1993-1999 | -0.15 | (-0.17, -0.12) | < 0.001 |  |  |  |
|  | Period3 | 1999-2005 | 0.08 | (0.05, 0.11) | < 0.001 |  |  |  |
|  | Period4 | 2005-2010 | -0.93 | (-0.96, -0.89) | < 0.001 |  |  |  |
|  | Period5 | 2010-2016 | 0.16 | (0.13, 0.18) | < 0.001 |  |  |  |
|  | Period6 | 2016-2021 | -0.04 | (-0.07, -0.01) | 0.008 |  |  |  |

Abbreviation: APC, annual percentage change; AAPC, average annual percentage change; *CI*, Confidence interval; GBD, global burden of disease; rIDPs, rare infectious diseases of poverty; SDI, social-demographic index.

**a** Temporal trend in age-standardized DALY rates for rIDPs were analyzed by the Joinpoint Regression Program (Version 5.2.0, National Cancer Institute: Rockville, MD, United States).

# Table S9. Temporal trends in age-standardized mortality rates of rIDPs by sex, SDI levels, and GBD super regions, from 1990 to 2021.

| **Groups** | **Time periods identified** | | **Temporal trend^a^** | | | | | |
| --- | --- | --- | --- | --- | --- | --- | --- | --- |
|  |  |  | **APC** | | | **AAPC** | | |
|  |  |  | **%** | **95% *CI*** | ***P* value** | **%** | **95% *CI*** | ***P* value** |
| Global | Period1 | 1990-1998 | -0.13 | (-0.24, -0.03) | 0.014 | 0.53 | (0.46, 0.60) | < 0.001 |
|  | Period2 | 1998-2004 | 1.42 | (1.23, 1.61) | < 0.001 |  |  |  |
|  | Period3 | 2004-2010 | 0.90 | (0.73, 1.06) | < 0.001 |  |  |  |
|  | Period4 | 2010-2016 | 1.18 | (0.99, 1.38) | < 0.001 |  |  |  |
|  | Period5 | 2016-2021 | -0.68 | (-0.91, -0.44) | < 0.001 |  |  |  |
| Male | Period1 | 1990-1998 | -0.02 | (-0.09, 0.05) | 0.570 | 0.50 | (0.44, 0.56) | < 0.001 |
|  | Period2 | 1998-2004 | 1.43 | (1.29, 1.57) | < 0.001 |  |  |  |
|  | Period3 | 2004-2011 | 0.70 | (0.59, 0.82) | < 0.001 |  |  |  |
|  | Period4 | 2011-2015 | 1.15 | (0.79, 1.51) | < 0.001 |  |  |  |
|  | Period5 | 2015-2021 | -0.39 | (-0.53, -0.25) | < 0.001 |  |  |  |
| Female | Period1 | 1990-1998 | -0.31 | (-0.43, -0.18) | < 0.001 | 0.53 | (0.45, 0.60) | < 0.001 |
|  | Period2 | 1998-2011 | 1.23 | (1.16, 1.29) | < 0.001 |  |  |  |
|  | Period3 | 2011-2016 | 1.48 | (1.15, 1.82) | < 0.001 |  |  |  |
|  | Period4 | 2016-2021 | -0.89 | (-1.17, -0.61) | < 0.001 |  |  |  |
| Low SDI | Period1 | 1990-1994 | 0.00 | (-0.37, 0.36) | 0.990 | -0.08 | (-0.21, 0.05) | 0.246 |
|  | Period2 | 1994-2001 | -0.46 | (-0.64, -0.29) | < 0.001 |  |  |  |
|  | Period3 | 2001-2011 | 0.19 | (0.11, 0.27) | < 0.001 |  |  |  |
|  | Period4 | 2011-2014 | 1.58 | (0.74, 2.42) | 0.001 |  |  |  |
|  | Period5 | 2014-2017 | 0.13 | (-0.78, 1.04) | 0.772 |  |  |  |
|  | Period6 | 2017-2021 | -1.52 | (-1.87, -1.18) | < 0.001 |  |  |  |
| Low-middle SDI | Period1 | 1990-2001 | -0.21 | (-0.28, -0.13) | < 0.001 | 0.27 | (0.16, 0.37) | < 0.001 |
|  | Period2 | 2001-2005 | 0.82 | (0.34, 1.30) | 0.002 |  |  |  |
|  | Period3 | 2005-2010 | 2.59 | (2.28, 2.89) | < 0.001 |  |  |  |
|  | Period4 | 2010-2017 | 0.37 | (0.17, 0.57) | 0.001 |  |  |  |
|  | Period5 | 2017-2021 | -1.99 | (-2.45, -1.54) | < 0.001 |  |  |  |
| Middle SDI | Period1 | 1990-1998 | -2.56 | (-2.64, -2.47) | < 0.001 | -1.10 | (-1.16, -1.03) | < 0.001 |
|  | Period2 | 1998-2004 | 0.63 | (0.43, 0.83) | < 0.001 |  |  |  |
|  | Period3 | 2004-2014 | -0.24 | (-0.35, -0.14) | < 0.001 |  |  |  |
|  | Period4 | 2014-2021 | -2.09 | (-2.27, -1.91) | < 0.001 |  |  |  |
| High-middle SDI | Period1 | 1990-1998 | -2.49 | (-2.69, -2.29) | < 0.001 | -1.80 | (-1.94, -1.66) | < 0.001 |
|  | Period2 | 1998-2003 | 3.77 | (3.10, 4.44) | < 0.001 |  |  |  |
|  | Period3 | 2003-2014 | -3.91 | (-4.10, -3.72) | < 0.001 |  |  |  |
|  | Period4 | 2014-2021 | -1.54 | (-1.83, -1.25) | < 0.001 |  |  |  |
| High SDI | Period1 | 1990-1993 | -2.16 | (-3.59, -0.71) | 0.006 | 0.30 | (-0.22, 0.82) | 0.258 |
|  | Period2 | 1993-2000 | 0.05 | (-0.64, 0.75) | 0.870 |  |  |  |
|  | Period3 | 2000-2003 | 7.54 | (2.63, 12.69) | 0.005 |  |  |  |
|  | Period4 | 2003-2010 | -1.98 | (-2.66, -1.30) | < 0.001 |  |  |  |
|  | Period5 | 2010-2017 | 1.85 | (1.22, 2.48) | < 0.001 |  |  |  |
|  | Period6 | 2017-2021 | -1.28 | (-2.52, -0.02) | 0.047 |  |  |  |
| Central Europe, Eastern Europe, and Central Asia | Period1 | 1990-1992 | -0.65 | (-4.51, 3.37) | 0.731 | -0.48 | (-0.98, 0.02) | 0.061 |
|  | Period2 | 1992-1996 | 1.76 | (0.00, 3.54) | 0.050 |  |  |  |
|  | Period3 | 1996-2000 | -0.66 | (-2.13, 0.84) | 0.361 |  |  |  |
|  | Period4 | 2000-2011 | -2.49 | (-2.74, -2.24) | < 0.001 |  |  |  |
|  | Period5 | 2011-2014 | 2.94 | (-0.66, 6.68) | 0.103 |  |  |  |
|  | Period6 | 2014-2021 | 0.16 | (-0.46, 0.78) | 0.597 |  |  |  |
| High income | Period1 | 1990-1994 | -2.84 | (-3.63, -2.05) | < 0.001 | 0.26 | (-0.23, 0.75) | 0.302 |
|  | Period2 | 1994-2000 | 0.88 | (0.16, 1.61) | 0.020 |  |  |  |
|  | Period3 | 2000-2003 | 5.66 | (1.39, 10.10) | 0.012 |  |  |  |
|  | Period4 | 2003-2009 | -2.23 | (-3.13, -1.33) | < 0.001 |  |  |  |
|  | Period5 | 2009-2018 | 2.06 | (1.62, 2.51) | < 0.001 |  |  |  |
|  | Period6 | 2018-2021 | -2.32 | (-4.53, -0.05) | 0.046 |  |  |  |
| Latin America and Caribbean | Period1 | 1990-1992 | -6.68 | (-7.55, -5.8) | < 0.001 | -3.12 | (-3.31, -2.92) | < 0.001 |
|  | Period2 | 1992-2005 | -4.96 | (-5.03, -4.88) | < 0.001 |  |  |  |
|  | Period3 | 2005-2013 | -2.05 | (-2.28, -1.82) | < 0.001 |  |  |  |
|  | Period4 | 2013-2016 | 5.97 | (4.02, 7.95) | < 0.001 |  |  |  |
|  | Period5 | 2016-2021 | -3.73 | (-4.25, -3.21) | < 0.001 |  |  |  |
| South Asia | Period1 | 1990-1996 | 0.33 | (-0.25, 0.92) | 0.250 | 0.24 | (-0.06, 0.54) | 0.113 |
|  | Period2 | 1996-2001 | -1.95 | (-3.00, -0.90) | 0.001 |  |  |  |
|  | Period3 | 2001-2005 | 2.29 | (0.76, 3.83) | 0.005 |  |  |  |
|  | Period4 | 2005-2010 | 6.72 | (5.89, 7.57) | < 0.001 |  |  |  |
|  | Period5 | 2010-2021 | -2.36 | (-2.56, -2.15) | < 0.001 |  |  |  |
| North Africa and Middle East | Period1 | 1990-2000 | -0.21 | (-0.3, -0.12) | < 0.001 | -1.10 | (-1.26, -0.94) | < 0.001 |
|  | Period2 | 2000-2003 | -2.91 | (-4.17, -1.63) | < 0.001 |  |  |  |
|  | Period3 | 2003-2009 | 1.06 | (0.75, 1.37) | < 0.001 |  |  |  |
|  | Period4 | 2009-2014 | -1.11 | (-1.60, -0.62) | < 0.001 |  |  |  |
|  | Period5 | 2014-2021 | -3.39 | (-3.58, -3.19) | < 0.001 |  |  |  |
| South-East Asia, East Asia, and Oceania | Period1 | 1990-1998 | -2.41 | (-2.65, -2.16) | < 0.001 | -1.35 | (-1.54, -1.12) | < 0.001 |
|  | Period2 | 1998-2004 | 4.38 | (3.74, 5.03) | < 0.001 |  |  |  |
|  | Period3 | 2004-2016 | -3.53 | (-3.75, -3.32) | < 0.001 |  |  |  |
|  | Period4 | 2016-2021 | -1.05 | (-1.82, -0.28) | 0.010 |  |  |  |
| Sub-Saharan Africa | Period1 | 1990-2000 | -0.27 | (-0.34, -0.20) | < 0.001 | -0.22 | (-0.33, -0.11) | < 0.001 |
|  | Period2 | 2000-2006 | 0.06 | (-0.12, 0.25) | 0.474 |  |  |  |
|  | Period3 | 2006-2010 | -0.43 | (-0.76, -0.10) | 0.013 |  |  |  |
|  | Period4 | 2010-2013 | 1.15 | (0.45, 1.86) | 0.003 |  |  |  |
|  | Period5 | 2013-2016 | 0.56 | (-0.17, 1.30) | 0.122 |  |  |  |
|  | Period6 | 2016-2021 | -1.59 | (-1.78, -1.39) | < 0.001 |  |  |  |

Abbreviation: APC, annual percentage change; AAPC, average annual percentage change; *CI*, Confidence interval; GBD, global burden of disease; rIDPs, rare infectious diseases of poverty; SDI, social-demographic index.

**a** Temporal trend in age-standardized DALY rates for rIDPs were analyzed by the Joinpoint Regression Program (Version 5.2.0, National Cancer Institute: Rockville, MD, United States).

# Table S10. Temporal trends in age-standardized DALY rates of rIDPs by GBD super regions, from 1990 to 2021.

| **Groups** | **Time periods identified** | | **Temporal trend^a^** | | | | | |
| --- | --- | --- | --- | --- | --- | --- | --- | --- |
|  |  |  | **APC** | | | **AAPC** | | |
|  |  |  | **%** | **95% *CI*** | ***P* value** | **%** | **95% *CI*** | ***P* value** |
| Central Europe, Eastern Europe, and Central Asia | Period1 | 1990-1999 | 0.19 | (0.15, 0.23) | < 0.001 | -0.80 | (-0.85, -0.74) | < 0.001 |
|  | Period2 | 1999-2002 | -1.05 | (-1.52, -0.57) | < 0.001 |  |  |  |
|  | Period3 | 2002-2010 | -2.04 | (-2.10, -1.97) | < 0.001 |  |  |  |
|  | Period4 | 2010-2017 | -0.96 | (-1.04, -0.88) | < 0.001 |  |  |  |
|  | Period5 | 2017-2021 | -0.02 | (-0.18, 0.13) | 0.756 |  |  |  |
| High Income | Period1 | 1990-1994 | -2.80 | (-3.10, -2.49) | < 0.001 | -0.67 | (-0.78, -0.56) | < 0.001 |
|  | Period2 | 1994-2000 | -1.28 | (-1.50, -1.06) | < 0.001 |  |  |  |
|  | Period3 | 2000-2003 | 0.63 | (-0.30, 1.57) | 0.167 |  |  |  |
|  | Period4 | 2003-2009 | -0.76 | (-0.95, -0.56) | < 0.001 |  |  |  |
|  | Period5 | 2009-2016 | 0.44 | (0.29, 0.60) | < 0.001 |  |  |  |
|  | Period6 | 2016-2021 | -0.43 | (-0.63, -0.23) | < 0.001 |  |  |  |
| Latin America and Caribbean | Period1 | 1990-1992 | -4.13 | (-4.72, -3.53) | < 0.001 | -2.12 | (-2.20, -2.03) | < 0.001 |
|  | Period2 | 1992-1999 | -3.22 | (-3.34, -3.10) | < 0.001 |  |  |  |
|  | Period3 | 1999-2005 | -2.46 | (-2.63, -2.28) | < 0.001 |  |  |  |
|  | Period4 | 2005-2012 | -1.71 | (-1.85, -1.56) | < 0.001 |  |  |  |
|  | Period5 | 2012-2016 | 0.37 | (-0.07, 0.81) | 0.096 |  |  |  |
|  | Period6 | 2016-2021 | -1.87 | (-2.07, -1.68) | < 0.001 |  |  |  |
| North Africa and Middle East | Period1 | 1990-2002 | -1.03 | (-1.05, -1.00) | < 0.001 | -1.02 | (-1.07, -0.97) | < 0.001 |
|  | Period2 | 2002-2006 | -0.57 | (-0.77, -0.38) | < 0.001 |  |  |  |
|  | Period3 | 2006-2009 | -1.25 | (-1.64, -0.85) | < 0.001 |  |  |  |
|  | Period4 | 2009-2015 | -0.82 | (-0.90, -0.73) | < 0.001 |  |  |  |
|  | Period5 | 2015-2021 | -1.40 | (-1.47, -1.33) | < 0.001 |  |  |  |
| South Asia | Period1 | 1990-1993 | -0.80 | (-0.91, -0.69) | < 0.001 | -0.90 | (-0.92, -0.87) | < 0.001 |
|  | Period2 | 1993-2000 | -0.64 | (-0.67, -0.60) | < 0.001 |  |  |  |
|  | Period3 | 2000-2003 | -0.83 | (-1.04, -0.61) | < 0.001 |  |  |  |
|  | Period4 | 2003-2010 | -0.71 | (-0.75, -0.67) | < 0.001 |  |  |  |
|  | Period5 | 2010-2021 | -1.23 | (-1.25, -1.22) | < 0.001 |  |  |  |
| South-East Asia, East Asia, and Oceania | Period1 | 1990-1998 | -1.59 | (-1.67, -1.51) | < 0.001 | -1.69 | (-1.80, -1.59) | < 0.001 |
|  | Period2 | 1998-2002 | -0.60 | (-0.97, -0.23) | 0.004 |  |  |  |
|  | Period3 | 2002-2005 | -2.03 | (-2.76, -1.30) | < 0.001 |  |  |  |
|  | Period4 | 2005-2009 | -2.71 | (-3.07, -2.34) | < 0.001 |  |  |  |
|  | Period5 | 2009-2018 | -1.99 | (-2.07, -1.90) | < 0.001 |  |  |  |
|  | Period6 | 2018-2021 | -0.85 | (-1.24, -0.47) | < 0.001 |  |  |  |
| Sub-Saharan Africa | Period1 | 1990-1999 | -0.40 | (-0.47, -0.34) | < 0.001 | -0.30 | (-0.35, -0.24) | < 0.001 |
|  | Period2 | 1999-2006 | 0.00 | (-0.10, 0.11) | 0.949 |  |  |  |
|  | Period3 | 2006-2010 | -0.92 | (-1.22, -0.63) | < 0.001 |  |  |  |
|  | Period4 | 2010-2016 | 0.79 | (0.66, 0.93) | < 0.001 |  |  |  |
|  | Period5 | 2016-2021 | -1.32 | (-1.48, -1.17) | < 0.001 |  |  |  |

Abbreviation: APC, annual percentage change; AAPC, average annual percentage change; CI, Confidence interval; DALYs, disability-adjusted life-years; GBD, global burden of disease; SDI, social-demographic index; rIDPs, rare infectious diseases of poverty.

**a** Temporal trend in age-standardized DALY rates for rIDPs were analyzed by the Joinpoint Regression Program (Version 5.2.0, National Cancer Institute: Rockville, MD, United States).

# Table S11. Temporal trends in age-standardized YLD rates of rIDPs by sex, SDI levels, and GBD super regions, from 1990 to 2021.

| **Groups** | **Time periods identified** | | **Temporal trend^a^** | | | | | |
| --- | --- | --- | --- | --- | --- | --- | --- | --- |
|  |  |  | **APC** | | | **AAPC** | | |
|  |  |  | **%** | **95% *CI*** | ***P* value** | **%** | **95% *CI*** | ***P* value** |
| Global | Period1 | 1990-1993 | -0.54 | (-0.67, -0.41) | < 0.001 | -0.58 | (-0.61, -0.54) | < 0.001 |
|  | Period2 | 1993-2002 | -0.21 | (-0.24, -0.18) | < 0.001 |  |  |  |
|  | Period3 | 2002-2006 | -0.58 | (-0.71, -0.45) | < 0.001 |  |  |  |
|  | Period4 | 2006-2009 | -1.25 | (-1.52, -0.98) | < 0.001 |  |  |  |
|  | Period5 | 2009-2019 | -0.73 | (-0.75, -0.70) | < 0.001 |  |  |  |
|  | Period6 | 2019-2021 | -0.47 | (-0.75, -0.20) | 0.002 |  |  |  |
| Male | Period1 | 1990-1993 | -0.78 | (-0.96, -0.60) | < 0.001 | -1.06 | (-1.10, -1.01) | < 0.001 |
|  | Period2 | 1993-2001 | -0.45 | (-0.50, -0.40) | < 0.001 |  |  |  |
|  | Period3 | 2001-2006 | -1.11 | (-1.23, -1.00) | < 0.001 |  |  |  |
|  | Period4 | 2006-2009 | -2.09 | (-2.45, -1.73) | < 0.001 |  |  |  |
|  | Period5 | 2009-2019 | -1.33 | (-1.36, -1.30) | < 0.001 |  |  |  |
|  | Period6 | 2019-2021 | -0.82 | (-1.19, -0.45) | < 0.001 |  |  |  |
| Female | Period1 | 1990-1994 | -0.33 | (-0.41, -0.24) | < 0.001 | -0.32 | (-0.35, -0.28) | < 0.001 |
|  | Period2 | 1994-2003 | -0.05 | (-0.08, -0.02) | 0.002 |  |  |  |
|  | Period3 | 2003-2006 | -0.28 | (-0.54, -0.01) | 0.044 |  |  |  |
|  | Period4 | 2006-2009 | -0.81 | (-1.08, -0.55) | < 0.001 |  |  |  |
|  | Period5 | 2009-2021 | -0.40 | (-0.42, -0.39) | < 0.001 |  |  |  |
| Low SDI | Period1 | 1990-1993 | -0.98 | (-1.13, -0.84) | < 0.001 | -0.75 | (-0.77, -0.72) | < 0.001 |
|  | Period2 | 1993-1998 | -0.76 | (-0.85, -0.66) | < 0.001 |  |  |  |
|  | Period3 | 1998-2005 | -0.49 | (-0.54, -0.44) | < 0.001 |  |  |  |
|  | Period4 | 2005-2010 | -1.56 | (-1.66, -1.46) | < 0.001 |  |  |  |
|  | Period5 | 2010-2021 | -0.47 | (-0.49, -0.45) | < 0.001 |  |  |  |
| Low-middle SDI | Period1 | 1990-1994 | -0.79 | (-0.82, -0.77) | < 0.001 | -0.99 | (-1.00, -0.98) | < 0.001 |
|  | Period2 | 1994-2002 | -0.58 | (-0.59, -0.57) | < 0.001 |  |  |  |
|  | Period3 | 2002-2006 | -0.98 | (-1.02, -0.94) | < 0.001 |  |  |  |
|  | Period4 | 2006-2009 | -1.58 | (-1.66, -1.50) | < 0.001 |  |  |  |
|  | Period5 | 2009-2016 | -1.14 | (-1.15, -1.12) | < 0.001 |  |  |  |
|  | Period6 | 2016-2021 | -1.26 | (-1.28, -1.25) | < 0.001 |  |  |  |
| Middle SDI | Period1 | 1990-2001 | -0.78 | (-0.80, -0.76) | < 0.001 | -1.05 | (-1.10, -1.01) | < 0.001 |
|  | Period2 | 2001-2006 | -1.03 | (-1.12, -0.93) | < 0.001 |  |  |  |
|  | Period3 | 2006-2009 | -1.34 | (-1.63, -1.04) | < 0.001 |  |  |  |
|  | Period4 | 2009-2015 | -1.05 | (-1.12, -0.99) | < 0.001 |  |  |  |
|  | Period5 | 2015-2018 | -1.88 | (-2.18, -1.58) | < 0.001 |  |  |  |
|  | Period6 | 2018-2021 | -1.01 | (-1.16, -0.86) | < 0.001 |  |  |  |
| High-middle SDI | Period1 | 1990-1998 | -1.18 | (-1.22, -1.14) | < 0.001 | -1.89 | (-1.93, -1.85) | < 0.001 |
|  | Period2 | 1998-2001 | -1.88 | (-2.25, -1.50) | < 0.001 |  |  |  |
|  | Period3 | 2001-2009 | -2.95 | (-3.00, -2.90) | < 0.001 |  |  |  |
|  | Period4 | 2009-2018 | -1.94 | (-1.98, -1.90) | < 0.001 |  |  |  |
|  | Period5 | 2018-2021 | -0.80 | (-1.00, -0.61) | < 0.001 |  |  |  |
| High SDI | Period1 | 1990-1994 | -2.44 | (-2.62, -2.26) | < 0.001 | -1.18 | (-1.23, -1.12) | < 0.001 |
|  | Period2 | 1994-2000 | -2.03 | (-2.16, -1.90) | < 0.001 |  |  |  |
|  | Period3 | 2000-2005 | -1.31 | (-1.49, -1.13) | < 0.001 |  |  |  |
|  | Period4 | 2005-2012 | -0.41 | (-0.51, -0.31) | < 0.001 |  |  |  |
|  | Period5 | 2012-2018 | -0.77 | (-0.90, -0.64) | < 0.001 |  |  |  |
|  | Period6 | 2018-2021 | -0.12 | (-0.41, 0.18) | 0.411 |  |  |  |
| Central Europe, Eastern Europe, and Central Asia | Period1 | 1990-1998 | 0.14 | (0.10, 0.17) | < 0.001 | -0.80 | (-0.84, -0.76) | < 0.001 |
|  | Period2 | 1998-2001 | -0.36 | (-0.67, -0.05) | 0.027 |  |  |  |
|  | Period3 | 2001-2010 | -1.91 | (-1.95, -1.88) | < 0.001 |  |  |  |
|  | Period4 | 2010-2014 | -1.29 | (-1.44, -1.14) | < 0.001 |  |  |  |
|  | Period5 | 2014-2018 | -0.85 | (-1.00, -0.70) | < 0.001 |  |  |  |
|  | Period6 | 2018-2021 | 0.33 | (0.17, 0.49) | < 0.001 |  |  |  |
| High income | Period1 | 1990-1994 | -2.62 | (-2.7, -2.55) | < 0.001 | -0.88 | (-0.91, -0.85) | < 0.001 |
|  | Period2 | 1994-2000 | -1.91 | (-1.96, -1.85) | < 0.001 |  |  |  |
|  | Period3 | 2000-2005 | -0.76 | (-0.83, -0.68) | < 0.001 |  |  |  |
|  | Period4 | 2005-2014 | 0.00 | (-0.03, 0.03) | 0.907 |  |  |  |
|  | Period5 | 2014-2019 | -0.43 | (-0.51, -0.35) | < 0.001 |  |  |  |
|  | Period6 | 2019-2021 | 0.36 | (0.11, 0.61) | 0.008 |  |  |  |
| Latin America & Caribbean | Period1 | 1990-2000 | -1.70 | (-1.72, -1.67) | < 0.001 | -1.09 | (-1.13, -1.05) | < 0.001 |
|  | Period2 | 2000-2005 | -0.64 | (-0.74, -0.54) | < 0.001 |  |  |  |
|  | Period3 | 2005-2011 | -1.08 | (-1.15, -1.01) | < 0.001 |  |  |  |
|  | Period4 | 2011-2015 | -0.60 | (-0.77, -0.44) | < 0.001 |  |  |  |
|  | Period5 | 2015-2019 | -1.12 | (-1.28, -0.95) | < 0.001 |  |  |  |
|  | Period6 | 2019-2021 | -0.05 | (-0.38, 0.28) | 0.751 |  |  |  |
| North Africa, and Middle East | Period1 | 1990-2000 | -1.09 | (-1.12, -1.06) | < 0.001 | -1.02 | (-1.05, -0.99) | < 0.001 |
|  | Period2 | 2000-2005 | -0.78 | (-0.90, -0.66) | < 0.001 |  |  |  |
|  | Period3 | 2005-2010 | -1.36 | (-1.48, -1.24) | < 0.001 |  |  |  |
|  | Period4 | 2010-2015 | -0.72 | (-0.84, -0.60) | < 0.001 |  |  |  |
|  | Period5 | 2015-2021 | -1.07 | (-1.14, -1.01) | < 0.001 |  |  |  |
| South Asia | Period1 | 1990-1994 | -0.83 | (-0.87, -0.79) | < 0.001 | -0.98 | (-1.00, -0.96) | < 0.001 |
|  | Period2 | 1994-2001 | -0.62 | (-0.64, -0.60) | < 0.001 |  |  |  |
|  | Period3 | 2001-2006 | -1.01 | (-1.05, -0.96) | < 0.001 |  |  |  |
|  | Period4 | 2006-2009 | -1.45 | (-1.59, -1.32) | < 0.001 |  |  |  |
|  | Period5 | 2009-2016 | -1.09 | (-1.12, -1.07) | < 0.001 |  |  |  |
|  | Period6 | 2016-2021 | -1.14 | (-1.17, -1.11) | < 0.001 |  |  |  |
| South-East Asia, East Asia, and Oceania | Period1 | 1990-1997 | -1.30 | (-1.37, -1.24) | < 0.001 | -1.75 | (-1.82, -1.68) | < 0.001 |
|  | Period2 | 1997-2000 | -1.61 | (-2.12, -1.10) | < 0.001 |  |  |  |
|  | Period3 | 2000-2009 | -2.54 | (-2.60, -2.49) | < 0.001 |  |  |  |
|  | Period4 | 2009-2014 | -1.32 | (-1.48, -1.16) | < 0.001 |  |  |  |
|  | Period5 | 2014-2018 | -1.99 | (-2.25, -1.73) | < 0.001 |  |  |  |
|  | Period6 | 2018-2021 | -0.93 | (-1.20, -0.66) | < 0.001 |  |  |  |
| Sub-Saharan Africa | Period1 | 1990-1998 | -0.40 | (-0.44, -0.37) | < 0.001 | -0.52 | (-0.54, -0.50) | < 0.001 |
|  | Period2 | 1998-2005 | -0.06 | (-0.12, -0.01) | 0.023 |  |  |  |
|  | Period3 | 2005-2010 | -1.65 | (-1.76, -1.55) | < 0.001 |  |  |  |
|  | Period4 | 2010-2021 | -0.37 | (-0.39, -0.35) | < 0.001 |  |  |  |

Abbreviation: APC, annual percentage change; AAPC, average annual percentage change; *CI*, Confidence interval; GBD, global burden of disease; rIDPs, rare infectious diseases of poverty; SDI, social-demographic index; YLD, years lived with disability.

**a** Temporal trend in age-standardized DALY rates for rIDPs were analyzed by the Joinpoint Regression Program (Version 5.2.0, National Cancer Institute: Rockville, MD, United States).

# Table S12. Temporal trends in age-standardized YLL rates of rIDPs by sex, SDI levels, and GBD super regions, from 1990 to 2021.

| **Groups** | **Time periods identified** | | **Temporal trend^a^** | | | | | |
| --- | --- | --- | --- | --- | --- | --- | --- | --- |
|  |  |  | **APC** | | | **AAPC** | | |
|  |  |  | **%** | **95% *CI*** | ***P* value** | **%** | **95% *CI*** | ***P* value** |
| Global | Period1 | 1990-1998 | 0.15 | (0.03, 0.27) | 0.016 | 0.77 | (0.67, 0.86) | < 0.001 |
|  | Period2 | 1998-2006 | 1.47 | (1.35, 1.58) | < 0.001 |  |  |  |
|  | Period3 | 2006-2009 | 0.68 | (-0.06, 1.43) | 0.071 |  |  |  |
|  | Period4 | 2009-2016 | 1.72 | (1.58, 1.86) | < 0.001 |  |  |  |
|  | Period5 | 2016-2021 | -0.63 | (-0.88, -0.39) | < 0.001 |  |  |  |
| Male | Period1 | 1990-1997 | 0.21 | (0.10, 0.33) | 0.001 | 0.83 | (0.76, 0.90) | < 0.001 |
|  | Period2 | 1997-2006 | 1.46 | (1.38, 1.54) | < 0.001 |  |  |  |
|  | Period3 | 2006-2010 | 0.75 | (0.40, 1.11) | < 0.001 |  |  |  |
|  | Period4 | 2010-2015 | 1.85 | (1.62, 2.09) | < 0.001 |  |  |  |
|  | Period5 | 2015-2021 | -0.17 | (-0.32, -0.01) | 0.035 |  |  |  |
| Female | Period1 | 1990-1998 | -0.11 | (-0.26, 0.04) | 0.137 | 0.66 | (0.58, 0.73) | < 0.001 |
|  | Period2 | 1998-2011 | 1.25 | (1.18, 1.31) | < 0.001 |  |  |  |
|  | Period3 | 2011-2016 | 2.02 | (1.69, 2.35) | < 0.001 |  |  |  |
|  | Period4 | 2016-2021 | -0.99 | (-1.27, -0.71) | < 0.001 |  |  |  |
| Low SDI | Period1 | 1990-2000 | -0.42 | (-0.60, -0.25) | < 0.001 | 0.05 | (-0.06, 0.15) | 0.391 |
|  | Period2 | 2000-2011 | 0.32 | (0.21, 0.44) | < 0.001 |  |  |  |
|  | Period3 | 2011-2016 | 1.81 | (1.40, 2.22) | < 0.001 |  |  |  |
|  | Period4 | 2016-2021 | -1.36 | (-1.70, -1.01) | < 0.001 |  |  |  |
| Low-middle SDI | Period1 | 1990-2000 | -0.15 | (-0.22, -0.09) | < 0.001 | 0.39 | (0.29, 0.48) | < 0.001 |
|  | Period2 | 2000-2005 | 0.74 | (0.48, 1.01) | < 0.001 |  |  |  |
|  | Period3 | 2005-2010 | 2.27 | (1.96, 2.58) | < 0.001 |  |  |  |
|  | Period4 | 2010-2017 | 1.13 | (0.93, 1.33) | < 0.001 |  |  |  |
|  | Period5 | 2017-2021 | -2.28 | (-2.74, -1.81) | < 0.001 |  |  |  |
| Middle SDI | Period1 | 1990-1998 | -2.71 | (-2.78, -2.64) | < 0.001 | -1.08 | (-1.16, -0.99) | < 0.001 |
|  | Period2 | 1998-2002 | 0.94 | (0.55, 1.34) | < 0.001 |  |  |  |
|  | Period3 | 2002-2008 | -0.28 | (-0.49, -0.06) | 0.014 |  |  |  |
|  | Period4 | 2008-2014 | 0.60 | (0.36, 0.84) | < 0.001 |  |  |  |
|  | Period5 | 2014-2021 | -2.44 | (-2.62, -2.27) | < 0.001 |  |  |  |
| High-middle SDI | Period1 | 1990-1998 | -3.08 | (-3.38, -2.79) | < 0.001 | -2.48 | (-2.91, -2.05) | < 0.001 |
|  | Period2 | 1998-2001 | 3.95 | (1.32, 6.66) | 0.006 |  |  |  |
|  | Period3 | 2001-2004 | 0.76 | (-2.67, 4.31) | 0.647 |  |  |  |
|  | Period4 | 2004-2012 | -5.11 | (-5.54, -4.69) | < 0.001 |  |  |  |
|  | Period5 | 2012-2016 | -3.65 | (-4.83, -2.46) | < 0.001 |  |  |  |
|  | Period6 | 2016-2021 | -1.97 | (-2.58, -1.35) | < 0.001 |  |  |  |
| High SDI | Period1 | 1990-1998 | -1.44 | (-1.92, -0.95) | < 0.001 | -0.02 | (-0.39, 0.36) | 0.936 |
|  | Period2 | 1998-2004 | 3.90 | (2.64, 5.16) | < 0.001 |  |  |  |
|  | Period3 | 2004-2010 | -2.60 | (-3.43, -1.77) | < 0.001 |  |  |  |
|  | Period4 | 2010-2018 | 1.59 | (1.12, 2.07) | < 0.001 |  |  |  |
|  | Period5 | 2018-2021 | -2.85 | (-4.99, -0.65) | 0.014 |  |  |  |
| Central Europe, Eastern Europe, and Central Asia | Period1 | 1990-1993 | -0.51 | (-1.67, 0.65) | 0.367 | -0.70 | (-1.00, -0.39) | < 0.001 |
|  | Period2 | 1993-1999 | 1.23 | (0.58, 1.89) | 0.001 |  |  |  |
|  | Period3 | 1999-2011 | -2.54 | (-2.71, -2.37) | < 0.001 |  |  |  |
|  | Period4 | 2011-2014 | 2.57 | (-0.05, 5.26) | 0.054 |  |  |  |
|  | Period5 | 2014-2021 | -0.60 | (-1.09, -0.11) | 0.019 |  |  |  |
| High income | Period1 | 1990-1994 | -3.45 | (-4.26, -2.64) | < 0.001 | 0.09 | (-0.41, 0.58) | 0.73 |
|  | Period2 | 1994-2000 | 1.18 | (0.42, 1.95) | 0.005 |  |  |  |
|  | Period3 | 2000-2003 | 5.81 | (1.6, 10.19) | 0.01 |  |  |  |
|  | Period4 | 2003-2010 | -2.04 | (-2.69, -1.4) | < 0.001 |  |  |  |
|  | Period5 | 2010-2018 | 2.17 | (1.67, 2.68) | < 0.001 |  |  |  |
|  | Period6 | 2018-2021 | -3.27 | (-5.73, -0.75) | 0.015 |  |  |  |
| Latin America and Caribbean | Period1 | 1990-1992 | -6.84 | (-8.03, -5.63) | < 0.001 | -3.80 | (-4.09, -3.52) | < 0.001 |
|  | Period2 | 1992-2006 | -5.29 | (-5.38, -5.21) | < 0.001 |  |  |  |
|  | Period3 | 2006-2013 | -2.84 | (-3.23, -2.44) | < 0.001 |  |  |  |
|  | Period4 | 2013-2016 | 4.69 | (1.93, 7.53) | 0.002 |  |  |  |
|  | Period5 | 2016-2021 | -4.60 | (-5.36, -3.84) | < 0.001 |  |  |  |
| South Asia | Period1 | 1990-1995 | 0.24 | (-0.4, 0.89) | 0.441 | 0.15 | (-0.17, 0.47) | 0.368 |
|  | Period2 | 1995-2002 | -1.05 | (-1.54, -0.57) | < 0.001 |  |  |  |
|  | Period3 | 2002-2005 | 2.64 | (0.02, 5.33) | 0.049 |  |  |  |
|  | Period4 | 2005-2010 | 6.28 | (5.6, 6.97) | < 0.001 |  |  |  |
|  | Period5 | 2010-2015 | -3.04 | (-3.8, -2.27) | < 0.001 |  |  |  |
|  | Period6 | 2015-2021 | -2.00 | (-2.41, -1.59) | < 0.001 |  |  |  |
| North Africa and Middle East | Period1 | 1990-1999 | -0.53 | (-0.69, -0.38) | < 0.001 | -1.02 | (-1.20, -0.84) | < 0.001 |
|  | Period2 | 1999-2003 | -1.41 | (-2.28, -0.54) | 0.003 |  |  |  |
|  | Period3 | 2003-2008 | 2.14 | (1.52, 2.76) | < 0.001 |  |  |  |
|  | Period4 | 2008-2014 | -0.58 | (-1.06, -0.09) | 0.023 |  |  |  |
|  | Period5 | 2014-2021 | -3.97 | (-4.27, -3.68) | < 0.001 |  |  |  |
| South-East Asia, East Asia, and Oceania | Period1 | 1990-1992 | -0.68 | (-1.98, 0.64) | 0.286 | -1.44 | (-1.63, -1.24) | < 0.001 |
|  | Period2 | 1992-1998 | -3.08 | (-3.34, -2.82) | < 0.001 |  |  |  |
|  | Period3 | 1998-2001 | 5.19 | (3.91, 6.49) | < 0.001 |  |  |  |
|  | Period4 | 2001-2004 | 2.69 | (1.33, 4.08) | 0.001 |  |  |  |
|  | Period5 | 2004-2016 | -3.53 | (-3.62, -3.45) | < 0.001 |  |  |  |
|  | Period6 | 2016-2021 | -0.92 | (-1.22, -0.62) | < 0.001 |  |  |  |
| Sub-Saharan Africa | Period1 | 1990-1999 | -0.41 | (-0.59, -0.23) | < 0.001 | -0.14 | (-0.24, -0.03) | 0.01 |
|  | Period2 | 1999-2010 | 0.03 | (-0.07, 0.14) | 0.53 |  |  |  |
|  | Period3 | 2010-2016 | 1.47 | (1.16, 1.78) | < 0.001 |  |  |  |
|  | Period4 | 2016-2021 | -1.91 | (-2.31, -1.51) | < 0.001 |  |  |  |

Abbreviation: APC, annual percentage change; AAPC, average annual percentage change; *CI*, Confidence interval; GBD, global burden of disease; rIDPs, rare infectious diseases of poverty; SDI, social-demographic index; YLL, years of life lost.

**a** Temporal trend in age-standardized DALY rates for rIDPs were analyzed by the Joinpoint Regression Program (Version 5.2.0, National Cancer Institute: Rockville, MD, United States).

# Table S13. Global distribution of age-standardized prevalence, mortality, DALY, YLD, and YLL rates (per 100,000 population) of rIDPs by countries and territories in 2021, and the percentage changes in the age-standardized rates from 1990 to 2021

| **Countries** | **Prevalence** | | **Mortality** | | **DALY** | | **YLD** | | **YLL** | |
| --- | --- | --- | --- | --- | --- | --- | --- | --- | --- | --- |
|  | **2021**  **(95% UI)** | **change, %^a^**  **(95% UI)** | **2021**  **(95% UI)** | **change, %^a^**  **(95% UI)** | **2021**  **(95% UI)** | **change, %^a^**  **(95% UI)** | **2021**  **(95% UI)** | **change, %^a^**  **(95% UI)** | **2021**  **(95% UI)** | **change, %^a^**  **(95% UI)** |
| **Central Europe, Eastern Europe, and Central Asia** | | | | | | | | | | |
| **Central Europe** |  |  |  |  |  |  |  |  |  |  |
| Albania | 1021.12  (906.76, 1242.91) | -0.42  (-0.54, -0.25) | 0.08  (0.04, 0.20) | -0.78  (-0.90, -0.46) | 18.71  (12.08, 27.58) | -0.58  (-0.69, -0.46) | 16.35  (10.23, 25.14) | -0.49  (-0.63, -0.36) | 2.37  (1.13, 5.43) | -0.82  (-0.92, -0.48) |
| Bosnia and Herzegovina | 956.20  (846.57, 1120.14) | -0.27  (-0.37, -0.12) | 0.01  (0.00, 0.02) | -0.58  (-0.78, -0.21) | 18.31  (11.36, 28.08) | -0.41  (-0.52, -0.29) | 17.99  (11.05, 27.67) | -0.41  (-0.52, -0.28) | 0.33  (0.07, 0.58) | -0.61  (-0.80, -0.25) |
| Bulgaria | 1037.33  (922.54, 1201.35) | -0.17  (-0.31, -0.02) | 0.09  (0.06, 0.14) | 0.49  (-0.03, 1.22) | 23.16  (15.81, 33.12) | -0.25  (-0.38, -0.10) | 19.63  (11.92, 29.11) | -0.30  (-0.43, -0.15) | 3.53  (2.30, 5.18) | 0.30  (-0.18, 0.90) |
| Croatia | 701.97  (627.45, 788.04) | -0.27  (-0.37, -0.16) | 0.00  (0.00, 0.00) | -0.84  (-0.88, -0.79) | 13.37  (8.60, 20.49) | -0.39  (-0.50, -0.26) | 13.31  (8.54, 20.43) | -0.38  (-0.50, -0.24) | 0.06  (0.05, 0.08) | -0.86  (-0.89, -0.82) |
| Czechia | 716.70  (639.50, 838.11) | -0.33  (-0.45, -0.20) | 0.01  (0.01, 0.01) | -0.25  (-0.46, 0.05) | 13.03  (8.24, 20.13) | -0.44  (-0.54, -0.32) | 12.73  (7.91, 19.81) | -0.44  (-0.54, -0.31) | 0.31  (0.23, 0.41) | -0.44  (-0.59, -0.19) |
| Hungary | 782.93  (694.15, 900.87) | -0.31  (-0.42, -0.18) | 0.03  (0.02, 0.04) | 0.48  (0.04, 1.00) | 15.88  (10.16, 23.34) | -0.41  (-0.52, -0.29) | 14.85  (9.18, 22.23) | -0.43  (-0.54, -0.31) | 1.04  (0.75, 1.42) | 0.15  (-0.20, 0.58) |
| Montenegro | 853.50  (752.48, 981.21) | -0.14  (-0.27, 0.02) | 0.00  (0.00, 0.01) | -0.11  (-0.44, 0.41) | 15.79  (9.92, 23.92) | -0.24  (-0.38, -0.07) | 15.74  (9.86, 23.85) | -0.24  (-0.38, -0.07) | 0.05  (0.00, 0.08) | -0.16  (-0.46, 0.32) |
| North Macedonia | 895.67  (814.10, 1009.45) | -0.27  (-0.37, -0.16) | 0.01  (0.00, 0.01) | -0.65  (-0.75, -0.32) | 19.64  (12.51, 29.70) | -0.41  (-0.53, -0.27) | 19.43  (12.31, 29.48) | -0.40  (-0.51, -0.25) | 0.21  (0.12, 0.30) | -0.80  (-0.87, -0.39) |
| Poland | 902.62  (774.59, 1085.89) | -0.34  (-0.45, -0.19) | 0.01  (0.01, 0.01) | -0.84  (-0.86, -0.82) | 15.92  (10.30, 23.83) | -0.50  (-0.59, -0.39) | 15.64  (10.01, 23.54) | -0.48  (-0.57, -0.35) | 0.28  (0.24, 0.32) | -0.87  (-0.89, -0.86) |
| Romania | 923.36  (813.99, 1066.27) | -0.32  (-0.42, -0.20) | 0.02  (0.01, 0.03) | -0.46  (-0.62, -0.24) | 17.96  (11.81, 27.43) | -0.48  (-0.57, -0.36) | 17.02  (10.70, 26.29) | -0.47  (-0.57, -0.35) | 0.94  (0.68, 1.27) | -0.59  (-0.72, -0.40) |
| Serbia | 869.92  (767.91, 1013.93) | -0.29  (-0.40, -0.16) | 0.04  (0.01, 0.05) | -0.12  (-0.47, 0.23) | 16.93  (11.23, 24.52) | -0.40  (-0.51, -0.27) | 16.06  (10.31, 23.77) | -0.41  (-0.52, -0.27) | 0.87  (0.27, 1.20) | -0.15  (-0.46, 0.24) |
| Slovakia | 821.75  (731.52, 961.93) | -0.28  (-0.40, -0.14) | 0.00  (0.00, 0.00) | -0.59  (-0.72, -0.29) | 14.90  (9.15, 22.19) | -0.41  (-0.52, -0.26) | 14.82  (9.07, 22.10) | -0.40  (-0.52, -0.25) | 0.08  (0.02, 0.11) | -0.62  (-0.74, -0.29) |
| Slovenia | 624.74  (558.27, 720.93) | -0.35  (-0.45, -0.23) | 0.00  (0.00, 0.00) | -0.91  (-0.93, -0.88) | 11.19  (7.09, 17.45) | -0.46  (-0.55, -0.34) | 11.15  (7.05, 17.41) | -0.44  (-0.54, -0.32) | 0.04  (0.03, 0.05) | -0.93  (-0.95, -0.91) |
| **Eastern Europe** |  |  |  |  |  |  |  |  |  |  |
| Belarus | 740.06  (665.89, 848.89) | -0.28  (-0.37, -0.16) | 0.02  (0.01, 0.02) | -0.30  (-0.66, 0.24) | 16.99  (11.35, 25.45) | -0.35  (-0.47, -0.22) | 15.90  (10.25, 24.31) | -0.35  (-0.47, -0.21) | 1.10  (0.73, 1.59) | -0.32  (-0.74, 0.50) |
| Estonia | 671.90  (610.40, 743.35) | -0.33  (-0.41, -0.22) | 0.01  (0.01, 0.01) | 0.51  (0.09, 1.05) | 14.17  (9.04, 20.92) | -0.41  (-0.52, -0.29) | 13.93  (8.83, 20.66) | -0.41  (-0.52, -0.30) | 0.24  (0.17, 0.30) | -0.13  (-0.38, 0.22) |
| Latvia | 774.28  (695.61, 872.13) | -0.24  (-0.34, -0.13) | 0.05  (0.04, 0.06) | -0.22  (-0.45, 0.13) | 18.98  (13.09, 27.17) | -0.33  (-0.45, -0.21) | 16.22  (10.42, 24.36) | -0.32  (-0.44, -0.18) | 2.76  (2.01, 3.63) | -0.39  (-0.58, -0.09) |
| Lithuania | 776.10  (701.61, 877.38) | -0.22  (-0.33, -0.10) | 0.03  (0.03, 0.04) | 1.26  (0.65, 2.05) | 17.39  (11.33, 25.54) | -0.28  (-0.40, -0.14) | 16.13  (10.02, 24.33) | -0.31  (-0.44, -0.17) | 1.25  (0.95, 1.58) | 0.55  (0.12, 1.14) |
| Moldova | 914.94  (830.27, 1034.16) | -0.30  (-0.42, -0.17) | 0.01  (0.00, 0.01) | -0.11  (-0.35, 0.23) | 18.70  (12.01, 27.86) | -0.34  (-0.46, -0.21) | 18.41  (11.69, 27.51) | -0.35  (-0.46, -0.22) | 0.30  (0.20, 0.41) | -0.03  (-0.33, 0.42) |
| Russia | 785.30  (707.81, 880.43) | -0.25  (-0.34, -0.15) | 0.07  (0.06, 0.07) | -0.26  (-0.35, -0.16) | 20.80  (15.00, 29.59) | -0.34  (-0.44, -0.24) | 17.14  (11.39, 25.97) | -0.34  (-0.44, -0.21) | 3.65  (3.20, 4.06) | -0.37  (-0.45, -0.29) |
| Ukraine | 806.55  (737.25, 896.43) | -0.12  (-0.21, -0.01) | 0.03  (0.02, 0.04) | -0.25  (-0.55, 0.15) | 22.95  (15.77, 32.93) | -0.18  (-0.32, -0.01) | 20.81  (13.80, 30.87) | -0.17  (-0.31, 0.00) | 2.14  (1.62, 2.80) | -0.29  (-0.60, 0.14) |
| **Central Asia** |  |  |  |  |  |  |  |  |  |  |
| Armenia | 1067.04  (973.71, 1186.57) | -0.18  (-0.27, -0.08) | 0.01  (0.01, 0.01) | 0.23  (-0.22, 0.93) | 29.70  (18.85, 44.66) | -0.32  (-0.45, -0.16) | 29.48  (18.65, 44.47) | -0.33  (-0.45, -0.16) | 0.22  (0.15, 0.30) | 0.11  (-0.32, 0.84) |
| Azerbaijan | 1388.98  (1278.44, 1536.06) | -0.23  (-0.31, -0.13) | 0.03  (0.01, 0.06) | -0.32  (-0.71, 0.80) | 40.38  (25.69, 58.38) | -0.34  (-0.46, -0.21) | 39.21  (24.51, 57.13) | -0.34  (-0.47, -0.21) | 1.16  (0.48, 2.46) | -0.31  (-0.70, 0.82) |
| Georgia | 1185.79  (1070.77, 1348.25) | -0.16  (-0.29, -0.02) | 0.49  (0.33, 0.69) | 4.73  (2.22, 8.95) | 53.44  (39.63, 71.24) | 0.21  (-0.04, 0.60) | 32.43  (20.97, 48.18) | -0.18  (-0.34, 0.01) | 21.01  (14.15, 30.81) | 3.56  (1.54, 7.11) |
| Kazakhstan | 1591.69  (1381.69, 1904.32) | -0.24  (-0.39, -0.06) | 0.05  (0.04, 0.08) | 0.38  (-0.17, 1.29) | 38.36  (25.56, 59.67) | -0.40  (-0.53, -0.23) | 35.67  (22.87, 56.51) | -0.42  (-0.56, -0.25) | 2.68  (1.80, 3.93) | 0.06  (-0.37, 0.80) |
| Kyrgyzstan | 1516.05  (1371.28, 1756.18) | -0.18  (-0.30, -0.05) | 0.20  (0.14, 0.27) | 0.01  (-0.41, 0.66) | 52.86  (38.37, 73.19) | -0.24  (-0.37, -0.09) | 40.31  (25.52, 60.89) | -0.28  (-0.42, -0.12) | 12.55  (8.75, 17.41) | -0.09  (-0.48, 0.51) |
| Mongolia | 1351.40  (1257.83, 1461.73) | -0.28  (-0.34, -0.20) | 0.02  (0.01, 0.03) | -0.69  (-0.85, -0.25) | 50.75  (33.44, 74.72) | -0.38  (-0.49, -0.27) | 49.85  (32.52, 73.85) | -0.37  (-0.46, -0.26) | 0.90  (0.29, 1.69) | -0.75  (-0.89, -0.19) |
| Tajikistan | 1630.95  (1515.31, 1753.00) | -0.07  (-0.17, 0.02) | 0.10  (0.05, 0.18) | -0.09  (-0.67, 1.25) | 59.66  (40.68, 84.66) | -0.15  (-0.30, 0.03) | 52.87  (34.38, 76.86) | -0.16  (-0.29, 0.02) | 6.78  (3.04, 13.27) | -0.05  (-0.68, 1.62) |
| Turkmenistan | 1433.48  (1304.98, 1602.62) | -0.20  (-0.29, -0.09) | 0.01  (0.00, 0.01) | -0.25  (-0.58, 0.32) | 43.26  (28.23, 65.08) | -0.32  (-0.45, -0.16) | 42.84  (27.80, 64.59) | -0.32  (-0.45, -0.16) | 0.42  (0.19, 0.65) | -0.19  (-0.60, 0.50) |
| Uzbekistan | 2148.21  (1908.24, 2508.81) | -0.13  (-0.26, 0.03) | 0.06  (0.04, 0.08) | 0.23  (-0.26, 1.06) | 66.28  (44.85, 94.97) | -0.16  (-0.30, 0.02) | 62.69  (41.16, 90.69) | -0.18  (-0.32, 0.00) | 3.59  (2.33, 5.10) | 0.26  (-0.25, 1.26) |
| **High income** | | | | | | | | | | |
| **Australasia** |  |  |  |  |  |  |  |  |  |  |
| Australia | 287.45  (219.70, 485.05) | -0.28  (-0.60, 0.39) | 0.01  (0.01, 0.02) | -0.42  (-0.52, -0.31) | 3.99  (2.45, 6.49) | -0.27  (-0.50, 0.08) | 3.59  (2.09, 6.07) | -0.23  (-0.49, 0.19) | 0.40  (0.32, 0.48) | -0.50  (-0.61, -0.38) |
| New Zealand | 368.46  (252.48, 614.57) | -0.25  (-0.57, 0.39) | 0.01  (0.00, 0.01) | -0.68  (-0.74, -0.62) | 5.08  (2.77, 10.00) | -0.32  (-0.64, 0.32) | 4.83  (2.53, 9.76) | -0.26  (-0.63, 0.46) | 0.25  (0.19, 0.31) | -0.72  (-0.78, -0.64) |
| **High-income Asia Pacific** |  |  |  |  |  |  |  |  |  |  |
| Brunei | 461.90  (390.23, 593.04) | -0.24  (-0.43, 0.00) | 0.01  (0.00, 0.01) | -0.42  (-0.57, -0.15) | 6.32  (3.73, 10.03) | -0.23  (-0.41, 0.01) | 6.16  (3.58, 9.85) | -0.22  (-0.41, 0.03) | 0.16  (0.02, 0.22) | -0.47  (-0.62, -0.21) |
| Japan | 377.10  (294.18, 476.76) | -0.24  (-0.46, 0.12) | 0.01  (0.01, 0.01) | -0.55  (-0.59, -0.52) | 4.28  (2.57, 7.01) | -0.29  (-0.46, -0.09) | 3.82  (2.12, 6.54) | -0.22  (-0.44, 0.05) | 0.46  (0.40, 0.51) | -0.59  (-0.63, -0.55) |
| South Korea | 251.22  (218.30, 295.79) | -0.55  (-0.64, -0.40) | 0.09  (0.02, 0.11) | 0.92  (-0.60, 1.59) | 4.98  (3.41, 6.97) | -0.35  (-0.54, -0.10) | 3.22  (1.97, 5.16) | -0.50  (-0.64, -0.31) | 1.76  (0.59, 2.21) | 0.58  (-0.57, 1.15) |
| Singapore | 375.74  (218.84, 644.14) | -0.53  (-0.76, -0.13) | 0.00  (0.00, 0.00) | -1.00  (-1.00, -1.00) | 5.69  (2.34, 10.56) | -0.56  (-0.78, -0.25) | 5.68  (2.34, 10.55) | -0.26  (-0.61, 0.35) | 0.01  (0.00, 0.01) | -1.00  (-1.00, -1.00) |
| **High-income North American** | | | | | | | | | | |
| Canada | 223.11  (154.19, 403.89) | -0.18  (-0.58, 0.72) | 0.03  (0.03, 0.03) | 0.40  (0.19, 0.64) | 3.35  (2.13, 5.24) | -0.01  (-0.37, 0.57) | 2.46  (1.25, 4.33) | -0.09  (-0.50, 0.69) | 0.89  (0.75, 1.07) | 0.29  (0.08, 0.56) |
| Greenland | 312.56  (272.32, 367.57) | -0.35  (-0.52, -0.19) | 0.13  (0.04, 0.18) | 0.04  (-0.37, 0.52) | 8.78  (5.65, 12.32) | -0.24  (-0.44, 0.03) | 4.40  (2.65, 7.09) | -0.32  (-0.49, -0.09) | 4.38  (1.87, 6.71) | -0.14  (-0.56, 0.45) |
| USA | 274.65  (244.09, 309.24) | -0.04  (-0.16, 0.11) | 0.07  (0.06, 0.08) | 1.21  (1.07, 1.35) | 7.00  (5.41, 9.25) | 0.33  (0.14, 0.54) | 4.25  (2.62, 6.49) | 0.10  (-0.10, 0.32) | 2.75  (2.45, 2.99) | 0.99  (0.83, 1.16) |
| **Western Europe** |  |  |  |  |  |  |  |  |  |  |
| Argentina | 666.26  (389.02, 1083.47) | -0.25  (-0.61, 0.36) | 0.06  (0.05, 0.06) | 0.33  (0.15, 0.56) | 8.96  (5.56, 14.51) | -0.25  (-0.57, 0.28) | 6.63  (3.24, 12.2) | -0.34  (-0.68, 0.32) | 2.32  (1.91, 2.75) | 0.17  (-0.01, 0.42) |
| Chile | 286.10  (251.14, 323.72) | -0.44  (-0.54, -0.33) | 0.04  (0.03, 0.04) | -0.46  (-0.53, -0.36) | 5.80  (3.98, 8.48) | -0.46  (-0.57, -0.34) | 4.58  (2.76, 7.31) | -0.45  (-0.58, -0.26) | 1.22  (1.04, 1.42) | -0.52  (-0.60, -0.43) |
| Uruguay | 527.58  (348.65, 837.92) | -0.34  (-0.65, 0.31) | 0.08  (0.07, 0.09) | 15.52  (12.72, 18.92) | 8.85  (6.06, 13.72) | 0.03  (-0.38, 0.76) | 5.41  (2.63, 10.19) | -0.35  (-0.64, 0.13) | 3.44  (2.77, 4.26) | 12.12  (9.41, 15.73) |
| **Southern Latin American** |  |  |  |  |  |  |  |  |  |  |
| Andorra | 257.11  (221.70, 303.83) | -0.24  (-0.41, -0.07) | 0.01  (0.01, 0.02) | -0.57  (-0.74, -0.30) | 4.38  (2.69, 6.70) | -0.34  (-0.51, -0.09) | 4.08  (2.44, 6.39) | -0.28  (-0.49, 0.00) | 0.30  (0.16, 0.47) | -0.67  (-0.81, -0.38) |
| Austria | 261.37  (229.30, 311.40) | -0.32  (-0.49, -0.15) | 0.02  (0.02, 0.02) | 0.99  (0.69, 1.35) | 4.98  (3.21, 7.55) | -0.32  (-0.50, -0.10) | 4.26  (2.43, 6.85) | -0.38  (-0.55, -0.14) | 0.72  (0.60, 0.85) | 0.55  (0.29, 0.90) |
| Belgium | 281.50  (244.15, 328.36) | -0.28  (-0.43, -0.13) | 0.02  (0.01, 0.02) | 0.22  (0.05, 0.43) | 5.50  (3.41, 8.13) | -0.30  (-0.48, -0.08) | 5.03  (2.89, 7.64) | -0.33  (-0.51, -0.10) | 0.47  (0.40, 0.57) | 0.12  (-0.05, 0.34) |
| Cyprus | 249.88  (212.51, 295.78) | -0.39  (-0.56, -0.23) | 0.02  (0.00, 0.03) | -0.77  (-0.86, -0.55) | 4.47  (2.75, 7.01) | -0.47  (-0.62, -0.25) | 4.22  (2.50, 6.69) | -0.43  (-0.60, -0.19) | 0.25  (0.03, 0.35) | -0.76  (-0.85, -0.55) |
| Denmark | 286.49  (244.23, 374.89) | -0.32  (-0.49, -0.07) | 0.03  (0.02, 0.03) | 0.25  (0.00, 0.56) | 5.23  (3.30, 7.69) | -0.33  (-0.50, -0.11) | 4.47  (2.49, 6.89) | -0.36  (-0.55, -0.10) | 0.77  (0.59, 0.94) | -0.05  (-0.36, 0.29) |
| Finland | 263.72  (229.59, 307.56) | -0.34  (-0.46, -0.21) | 0.00  (0.00, 0.01) | -0.04  (-0.20, 0.19) | 4.63  (2.84, 6.95) | -0.38  (-0.54, -0.17) | 4.47  (2.67, 6.79) | -0.38  (-0.55, -0.17) | 0.15  (0.12, 0.18) | -0.20  (-0.34, 0.01) |
| France | 266.91  (234.87, 306.38) | -0.33  (-0.47, -0.19) | 0.02  (0.01, 0.02) | -0.68  (-0.73, -0.62) | 5.67  (3.80, 8.37) | -0.45  (-0.58, -0.30) | 5.11  (3.25, 7.85) | -0.38  (-0.55, -0.19) | 0.56  (0.46, 0.68) | -0.72  (-0.77, -0.66) |
| Germany | 291.66  (214.44, 458.19) | -0.38  (-0.63, 0.10) | 0.02  (0.02, 0.03) | 0.88  (0.63, 1.17) | 4.67  (2.91, 7.08) | -0.35  (-0.55, -0.02) | 3.95  (2.20, 6.41) | -0.41  (-0.62, -0.08) | 0.72  (0.61, 0.85) | 0.68  (0.43, 0.98) |
| Greece | 313.05  (267.93, 376.54) | -0.16  (-0.35, 0.06) | 0.02  (0.02, 0.02) | -0.44  (-0.53, -0.33) | 5.79  (3.50, 9.07) | -0.24  (-0.43, 0.02) | 5.39  (3.10, 8.65) | -0.22  (-0.42, 0.10) | 0.40  (0.33, 0.47) | -0.46  (-0.55, -0.36) |
| Iceland | 237.10  (205.72, 276.04) | -0.29  (-0.43, -0.14) | 0.02  (0.01, 0.02) | 0.28  (0.00, 0.63) | 4.27  (2.61, 6.73) | -0.31  (-0.48, -0.08) | 4.03  (2.38, 6.47) | -0.33  (-0.50, -0.09) | 0.24  (0.20, 0.29) | 0.23  (-0.03, 0.55) |
| Ireland | 241.92  (209.02, 290.00) | -0.40  (-0.53, -0.25) | 0.01  (0.01, 0.01) | -0.55  (-0.64, -0.46) | 4.29  (2.76, 6.65) | -0.48  (-0.60, -0.32) | 3.93  (2.39, 6.30) | -0.47  (-0.61, -0.30) | 0.36  (0.27, 0.44) | -0.57  (-0.67, -0.44) |
| Israel | 336.26  (289.65, 407.64) | -0.31  (-0.47, -0.12) | 0.08  (0.06, 0.10) | 7.76  (6.32, 9.71) | 7.40  (5.01, 10.64) | -0.18  (-0.37, 0.09) | 5.55  (3.19, 8.66) | -0.37  (-0.54, -0.14) | 1.84  (1.50, 2.21) | 6.71  (5.41, 8.46) |
| Italy | 235.81  (190.31, 319.45) | -0.30  (-0.50, -0.04) | 0.03  (0.03, 0.03) | -0.44  (-0.50, -0.38) | 4.75  (3.10, 6.86) | -0.38  (-0.52, -0.21) | 3.88  (2.20, 5.96) | -0.33  (-0.51, -0.07) | 0.87  (0.74, 0.99) | -0.54  (-0.59, -0.49) |
| Luxembourg | 248.16  (212.89, 293.09) | -0.38  (-0.55, -0.23) | 0.04  (0.03, 0.05) | 1.14  (0.75, 1.59) | 4.60  (3.12, 7.03) | -0.36  (-0.51, -0.16) | 3.80  (2.30, 6.22) | -0.44  (-0.59, -0.24) | 0.81  (0.63, 1.00) | 0.88  (0.54, 1.30) |
| Malta | 308.49  (260.79, 366.07) | -0.35  (-0.48, -0.17) | 0.00  (0.00, 0) | -0.93  (-0.94, -0.92) | 5.22  (3.20, 8.02) | -0.50  (-0.63, -0.33) | 5.10  (3.07, 7.91) | -0.41  (-0.57, -0.18) | 0.12  (0.09, 0.15) | -0.93  (-0.94, -0.91) |
| Monaco | 198.48  (171.60, 238.23) | -0.22  (-0.39, -0.02) | 0.02  (0.00, 0.03) | 0.38  (-0.16, 1.15) | 3.56  (2.25, 5.34) | -0.20  (-0.39, 0.05) | 2.64  (1.58, 4.14) | -0.25  (-0.45, 0.01) | 0.92  (0.15, 1.42) | -0.06  (-0.48, 0.71) |
| Netherlands | 271.05  (234.27, 313.64) | -0.27  (-0.40, -0.12) | 0.04  (0.03, 0.05) | 0.40  (0.18, 0.65) | 5.61  (3.80, 7.87) | -0.28  (-0.45, -0.08) | 4.45  (2.63, 6.81) | -0.34  (-0.52, -0.11) | 1.16  (0.95, 1.40) | 0.10  (-0.11, 0.33) |
| Norway | 228.80  (192.11, 288.84) | -0.37  (-0.53, -0.18) | 0.04  (0.04, 0.05) | 1.37  (1.13, 1.65) | 4.41  (2.87, 6.65) | -0.34  (-0.51, -0.13) | 3.67  (2.10, 5.95) | -0.42  (-0.58, -0.21) | 0.75  (0.66, 0.84) | 0.91  (0.73, 1.15) |
| Portugal | 330.73  (284.86, 389.29) | -0.39  (-0.53, -0.24) | 0.04  (0.04, 0.05) | -0.77  (-0.81, -0.74) | 6.61  (4.29, 10.00) | -0.57  (-0.67, -0.47) | 5.50  (3.18, 8.86) | -0.44  (-0.59, -0.24) | 1.11  (0.95, 1.29) | -0.81  (-0.83, -0.77) |
| San Marino | 261.57  (226.15, 314.45) | -0.18  (-0.33, 0.03) | 0.06  (0.00, 0.1) | -0.58  (-0.75, -0.33) | 4.83  (3.10, 7.41) | -0.31  (-0.46, -0.06) | 4.17  (2.52, 6.62) | -0.23  (-0.42, 0.06) | 0.65  (0.02, 1.05) | -0.57  (-0.74, -0.33) |
| Spain | 264.16  (225.54, 330.64) | -0.35  (-0.52, -0.15) | 0.02  (0.01, 0.02) | -0.80  (-0.83, -0.76) | 5.04  (3.07, 7.59) | -0.52  (-0.64, -0.39) | 4.51  (2.54, 7.05) | -0.38  (-0.55, -0.15) | 0.53  (0.44, 0.61) | -0.84  (-0.86, -0.81) |
| Sweden | 268.39  (224.72, 337.55) | -0.27  (-0.45, -0.02) | 0.03  (0.03, 0.04) | 0.83  (0.55, 1.15) | 5.09  (3.33, 7.62) | -0.26  (-0.44, 0.00) | 4.24  (2.44, 6.72) | -0.32  (-0.51, -0.06) | 0.85  (0.70, 1.00) | 0.42  (0.20, 0.69) |
| Switzerland | 304.77  (216.00, 487.66) | -0.23  (-0.55, 0.39) | 0.02  (0.02, 0.03) | 0.13  (-0.07, 0.35) | 4.22  (2.55, 7.24) | -0.28  (-0.51, 0.09) | 3.67  (1.99, 6.63) | -0.30  (-0.56, 0.11) | 0.55  (0.45, 0.64) | -0.04  (-0.25, 0.20) |
| UK | 358.44  (300.61, 442.41) | -0.18  (-0.36, 0.04) | 0.01  (0.01, 0.01) | -0.79  (-0.80, -0.78) | 7.44  (4.80, 11.58) | -0.30  (-0.47, -0.06) | 7.20  (4.54, 11.32) | -0.22  (-0.42, 0.07) | 0.25  (0.22, 0.27) | -0.82  (-0.83, -0.81) |
| **Latin America and Caribbean** | | | | | | | | | | |
| **Andean Latin America** |  |  |  |  |  |  |  |  |  |  |
| Bolivia | 1476.91  (1202.27, 1780.35) | -0.29  (-0.44, -0.10) | 0.17  (0.10, 0.29) | -0.49  (-0.67, -0.16) | 48.13  (32.73, 68.37) | -0.41  (-0.52, -0.26) | 40.07  (25.86, 59.86) | -0.37  (-0.48, -0.22) | 8.06  (4.04, 14.45) | -0.55  (-0.76, -0.18) |
| Ecuador | 411.82  (373.20, 456.98) | -0.45  (-0.63, -0.29) | 0.13  (0.10, 0.15) | -0.70  (-0.76, -0.64) | 12.71  (9.81, 16.63) | -0.64  (-0.71, -0.57) | 7.33  (4.65, 11.19) | -0.44  (-0.58, -0.25) | 5.37  (4.28, 6.55) | -0.76  (-0.81, -0.71) |
| Peru | 971.85  (855.65, 1272.87) | -0.42  (-0.53, -0.24) | 0.13  (0.09, 0.18) | -0.54  (-0.69, -0.33) | 24.60  (17.36, 35.16) | -0.55  (-0.64, -0.44) | 18.75  (11.56, 28.95) | -0.52  (-0.64, -0.40) | 5.86  (3.87, 8.32) | -0.62  (-0.76, -0.35) |
| **Central Latin America** |  |  |  |  |  |  |  |  |  |  |
| Colombia | 501.23  (451.33, 551.52) | -0.43  (-0.52, -0.33) | 0.09  (0.07, 0.12) | -0.80  (-0.85, -0.75) | 17.02  (12.62, 23.30) | -0.65  (-0.72, -0.57) | 11.43  (7.31, 17.58) | -0.38  (-0.52, -0.23) | 5.59  (4.12, 7.48) | -0.81  (-0.86, -0.75) |
| Costa Rica | 604.23  (508.40, 736.64) | -0.22  (-0.39, 0.00) | 0.02  (0.02, 0.03) | -0.78  (-0.83, -0.73) | 11.97  (7.88, 18.08) | -0.41  (-0.53, -0.25) | 10.56  (6.55, 16.68) | -0.19  (-0.37, 0.04) | 1.40  (1.05, 1.85) | -0.80  (-0.85, -0.74) |
| El Salvador | 604.33  (538.74, 706.05) | -0.22  (-0.33, -0.08) | 0.19  (0.14, 0.25) | -0.2  (-0.46, 0.09) | 21.08  (15.23, 28.54) | -0.36  (-0.51, -0.19) | 11.44  (7.34, 17.91) | -0.36  (-0.49, -0.21) | 9.64  (6.17, 13.99) | -0.36  (-0.60, -0.05) |
| Guatemala | 1227.66  (1117.90, 1415.88) | -0.17  (-0.27, -0.02) | 0.28  (0.22, 0.36) | -0.88  (-0.91, -0.85) | 47.26  (35.93, 64.19) | -0.77  (-0.82, -0.71) | 30.56  (19.51, 45.94) | -0.27  (-0.39, -0.11) | 16.70  (12.29, 22.43) | -0.90  (-0.93, -0.86) |
| Honduras | 920.43  (829.15, 1068.22) | -0.17  (-0.27, 0.00) | 0.27  (0.05, 0.54) | -0.45  (-0.79, 0.06) | 39.94  (19.55, 65.75) | -0.38  (-0.64, -0.02) | 19.76  (12.42, 29.05) | -0.25  (-0.39, -0.07) | 20.18  (2.83, 44.14) | -0.47  (-0.86, 0.13) |
| Mexico | 493.24  (481.91, 504.39) | -0.23  (-0.26, -0.21) | 0.18  (0.16, 0.21) | -0.78  (-0.81, -0.75) | 19.49  (15.37, 24.59) | -0.69  (-0.73, -0.63) | 11.04  (7.21, 16.10) | -0.31  (-0.35, -0.26) | 8.46  (7.19, 10.05) | -0.82  (-0.85, -0.78) |
| Nicaragua | 696.37  (623.69, 779.44) | -0.24  (-0.34, -0.13) | 0.06  (0.04, 0.14) | -0.82  (-0.90, -0.14) | 20.29  (13.92, 29.18) | -0.56  (-0.68, -0.18) | 16.53  (10.71, 24.67) | -0.24  (-0.40, -0.03) | 3.76  (2.08, 9.55) | -0.85  (-0.92, -0.06) |
| Panama | 764.94  (639.21, 942.57) | -0.26  (-0.43, -0.06) | 0.12  (0.09, 0.16) | -0.64  (-0.72, -0.52) | 20.11  (14.87, 27.37) | -0.52  (-0.61, -0.41) | 12.08  (7.20, 19.24) | -0.33  (-0.48, -0.16) | 8.02  (6.05, 10.66) | -0.66  (-0.76, -0.54) |
| Venezuela | 712.51  (605.02, 879.72) | -0.26  (-0.41, -0.04) | 0.15  (0.11, 0.20) | -0.57  (-0.69, -0.42) | 23.10  (16.85, 31.78) | -0.45  (-0.56, -0.33) | 14.18  (8.64, 22.15) | -0.29  (-0.45, -0.10) | 8.92  (6.02, 12.39) | -0.59  (-0.72, -0.43) |
| **Tropical Latin America** |  |  |  |  |  |  |  |  |  |  |
| Brazil | 1209.26  (1087.99, 1374.91) | -0.23  (-0.33, -0.10) | 0.23  (0.21, 0.25) | -0.35  (-0.47, -0.25) | 46.27  (33.71, 62.50) | -0.35  (-0.45, -0.23) | 34.84  (22.48, 51.06) | -0.27  (-0.40, -0.12) | 11.43  (9.83, 13.11) | -0.50  (-0.64, -0.38) |
| Paraguay | 983.13  (873.92, 1128.93) | -0.20  (-0.32, -0.07) | 0.11  (0.07, 0.15) | -0.10  (-0.34, 0.24) | 29.04  (20.76, 41.78) | -0.24  (-0.38, -0.08) | 23.67  (15.27, 35.55) | -0.25  (-0.41, -0.06) | 5.37  (3.62, 7.80) | -0.19  (-0.44, 0.20) |
| **Caribbean** |  |  |  |  |  |  |  |  |  |  |
| Antigua and Barbuda | 1394.90  (1165.23, 1758.97) | -0.20  (-0.37, 0.04) | 0.18  (0.15, 0.21) | 9.56  (7.79, 11.70) | 31.16  (21.34, 46.78) | -0.14  (-0.36, 0.17) | 25.95  (16.00, 41.72) | -0.27  (-0.48, 0.03) | 5.21  (4.37, 6.14) | 9.32  (7.53, 11.41) |
| Bahamas | 1362.72  (1174.50, 1631.36) | -0.13  (-0.33, 0.08) | 0.05  (0.04, 0.06) | 15.26  (11.19, 20.14) | 31.06  (20.21, 44.94) | -0.14  (-0.30, 0.09) | 29.15  (18.55, 43.18) | -0.19  (-0.34, 0.01) | 1.91  (1.42, 2.56) | 15.08  (10.57, 20.80) |
| Barbados | 1201.73  (1051.47, 1495.23) | -0.17  (-0.33, 0.06) | 0.26  (0.19, 0.33) | 33.23  (23.00, 45.34) | 37.68  (26.85, 51.94) | 0.05  (-0.17, 0.34) | 27.90  (17.09, 42.26) | -0.21  (-0.38, -0.01) | 9.78  (7.09, 13.47) | 29.29  (18.71, 42.39) |
| Belize | 1583.71  (1397.35, 1898.53) | -0.08  (-0.24, 0.13) | 0.14  (0.11, 0.17) | 10.72  (8.52, 13.72) | 43.18  (29.85, 63.03) | -0.06  (-0.24, 0.17) | 37.69  (24.16, 57.90) | -0.17  (-0.34, 0.05) | 5.49  (4.36, 6.88) | 7.04  (5.25, 9.50) |
| Bermuda | 751.58  (656.95, 889.65) | -0.35  (-0.47, -0.19) | 0.01  (0.00, 0.01) | -0.58  (-0.69, -0.45) | 13.73  (8.51, 20.84) | -0.42  (-0.54, -0.30) | 13.55  (8.35, 20.63) | -0.42  (-0.54, -0.29) | 0.18  (0.13, 0.25) | -0.59  (-0.71, -0.43) |
| Cuba | 1055.97  (924.44, 1276.74) | -0.14  (-0.29, 0.08) | 0.02  (0.02, 0.03) | -0.56  (-0.64, -0.49) | 23.72  (15.51, 34.30) | -0.24  (-0.38, -0.10) | 22.65  (14.32, 33.17) | -0.21  (-0.36, -0.03) | 1.07  (0.86, 1.34) | -0.63  (-0.71, -0.55) |
| Dominica | 1328.34  (1203.64, 1456.55) | -0.14  (-0.24, -0.02) | 0.08  (0.00, 0.11) | 3.92  (-0.39, 5.62) | 36.75  (23.51, 52.19) | -0.14  (-0.30, 0.04) | 35.38  (22.28, 50.82) | -0.17  (-0.31, 0.01) | 1.36  (0.07, 1.93) | 3.48  (-0.21, 5.10) |
| Dominican Republic | 1237.59  (1132.57, 1358.04) | -0.22  (-0.31, -0.11) | 0.09  (0.05, 0.19) | -0.50  (-0.68, -0.30) | 37.27  (24.37, 54.04) | -0.34  (-0.46, -0.21) | 32.79  (21.17, 49.38) | -0.30  (-0.43, -0.13) | 4.48  (1.99, 7.27) | -0.55  (-0.78, -0.25) |
| Grenada | 1364.46  (1193.19, 1604.47) | -0.16  (-0.27, 0.01) | 0.20  (0.17, 0.23) | 0.10  (-0.09, 0.31) | 40.23  (27.46, 57.04) | -0.26  (-0.37, -0.11) | 33.37  (20.89, 50.75) | -0.29  (-0.42, -0.12) | 6.86  (5.63, 8.31) | -0.07  (-0.25, 0.14) |
| Guyana | 1645  (1467.59, 1982.74) | -0.24  (-0.36, -0.08) | 0.98  (0.75, 1.28) | 0.88  (0.42, 1.46) | 74.27  (58.51, 97.18) | 0.09  (-0.11, 0.39) | 36.05  (22.88, 54.70) | -0.23  (-0.38, -0.02) | 38.22  (28.28, 51.8) | 0.81  (0.29, 1.56) |
| Haiti | 2717.21  (2456.05, 3020.26) | 0.05  (-0.09, 0.20) | 0.12  (0.05, 0.19) | 0.17  (-0.28, 0.74) | 75.62  (49.49, 112.75) | -0.20  (-0.38, 0.02) | 72.03  (45.55, 108.76) | -0.22  (-0.39, 0.00) | 3.59  (1.53, 5.26) | 0.09  (-0.38, 0.69) |
| Jamaica | 1452.78  (1265.91, 1746.30) | -0.10  (-0.25, 0.13) | 0.34  (0.26, 0.45) | 11.01  (7.88, 14.20) | 42.98  (31.21, 59) | 0.13  (-0.08, 0.44) | 29.80  (18.64, 45.71) | -0.18  (-0.34, 0.02) | 13.18  (9.64, 17.63) | 7.76  (5.23, 10.76) |
| Puerto Rico | 891.18  (773.75, 1075.37) | -0.27  (-0.43, -0.11) | 0.07  (0.06, 0.08) | 1.93  (1.40, 2.62) | 20.02  (12.95, 29.89) | -0.26  (-0.39, -0.08) | 17.45  (10.47, 27.20) | -0.33  (-0.47, -0.17) | 2.57  (2.07, 3.16) | 1.85  (1.30, 2.5) |
| Saint Kitts and Nevis | 1384.05  (1201.78, 1658.83) | -0.27  (-0.41, -0.10) | 0.08  (0.07, 0.11) | 7.18  (5.28, 9.52) | 30.11  (18.25, 44.35) | -0.38  (-0.50, -0.24) | 29.17  (17.21, 43.40) | -0.40  (-0.52, -0.26) | 0.94  (0.74, 1.14) | 4.27  (3.12, 5.57) |
| Saint Lucia | 1434.47  (1262.84, 1732.92) | -0.20  (-0.33, -0.02) | 0.40  (0.32, 0.49) | 4.75  (3.55, 6.03) | 45.70  (33.01, 62.64) | -0.06  (-0.24, 0.19) | 32.53  (20.40, 48.55) | -0.30  (-0.44, -0.13) | 13.17  (9.90, 17.55) | 4.68  (3.15, 6.75) |
| Saint Vincent and the Grenadines | 1512.57  (1329.24, 1847.91) | -0.11  (-0.28, 0.10) | 0.29  (0.25, 0.35) | -0.48  (-0.58, -0.36) | 42.43  (29.67, 58.26) | -0.34  (-0.45, -0.20) | 34.49  (21.53, 50.51) | -0.21  (-0.36, 0.00) | 7.94  (6.40, 9.83) | -0.61  (-0.70, -0.49) |
| Suriname | 1627.07  (1429.59, 1924.07) | -0.10  (-0.26, 0.10) | 0.17  (0.09, 0.24) | 0.79  (-0.26, 1.59) | 43.65  (29.21, 61.39) | -0.13  (-0.31, 0.10) | 37.26  (23.72, 54.77) | -0.20  (-0.37, 0.02) | 6.39  (2.87, 9.72) | 0.72  (-0.16, 1.52) |
| Trinidad and Tobago | 1360.08  (1183.10, 1636.33) | -0.21  (-0.36, -0.02) | 0.20  (0.15, 0.25) | 1.72  (1.05, 2.47) | 36.37  (25.35, 51.36) | -0.20  (-0.34, -0.02) | 28.64  (17.81, 43.41) | -0.33  (-0.46, -0.18) | 7.72  (5.65, 10.56) | 1.94  (1.15, 2.87) |
| Virgin Islands | 1162.46  (997.70, 1432.98) | -0.17  (-0.34, 0.04) | 0.01  (0.00, 0.02) | -0.53  (-0.67, -0.29) | 22.30  (14.14, 33.76) | -0.25  (-0.40, -0.07) | 22.14  (13.97, 33.64) | -0.25  (-0.40, -0.06) | 0.16  (0.02, 0.23) | -0.60  (-0.72, -0.26) |
| **North Africa and Middle East** | | | | | | | | | | |
| Afghanistan | 1072.93  (981.24, 1166.90) | -0.25  (-0.34, -0.16) | 0.10  (0.03, 0.13) | 0.01  (-0.34, 0.46) | 32.71  (21.52, 45.50) | -0.32  (-0.46, -0.15) | 28.49  (17.96, 41.29) | -0.35  (-0.48, -0.18) | 4.23  (0.95, 6.10) | -0.02  (-0.45, 0.54) |
| Algeria | 1233.47  (1096.98, 1448.55) | -0.28  (-0.38, -0.13) | 0.04  (0.01, 0.06) | -0.19  (-0.46, 0.13) | 29.35  (18.56, 44.44) | -0.34  (-0.46, -0.18) | 27.68  (17.02, 42.73) | -0.34  (-0.47, -0.17) | 1.67  (0.36, 2.38) | -0.33  (-0.64, 0.01) |
| Bahrain | 886.03  (764.28, 1040.43) | -0.37  (-0.47, -0.23) | 0.02  (0.02, 0.03) | -0.04  (-0.50, 0.29) | 16.88  (10.96, 25.88) | -0.43  (-0.56, -0.29) | 16.35  (10.41, 25.34) | -0.44  (-0.57, -0.29) | 0.53  (0.41, 0.67) | -0.23  (-0.54, 0.05) |
| Egypt | 1092.94  (1008.62, 1202.65) | -0.29  (-0.38, -0.18) | 0.11  (0.06, 0.15) | 3.63  (-0.29, 8.07) | 27.16  (19.14, 38.65) | -0.26  (-0.42, -0.08) | 21.31  (13.44, 32.23) | -0.40  (-0.52, -0.27) | 5.85  (2.18, 8.33) | 3.36  (-0.32, 6.81) |
| Iran | 928.59  (830.76, 1030.20) | -0.33  (-0.40, -0.24) | 0.07  (0.05, 0.09) | -0.35  (-0.53, -0.13) | 27.01  (18.47, 38.95) | -0.38  (-0.48, -0.26) | 23.66  (15.42, 35.43) | -0.36  (-0.47, -0.21) | 3.35  (1.92, 4.33) | -0.48  (-0.70, -0.24) |
| Iraq | 1069.50  (945.87, 1213.64) | -0.28  (-0.37, -0.15) | 0.04  (0.02, 0.05) | -0.37  (-0.65, 0.00) | 27.60  (17.80, 40.85) | -0.36  (-0.48, -0.21) | 25.32  (15.79, 38.37) | -0.36  (-0.48, -0.20) | 2.28  (1.25, 3.36) | -0.39  (-0.70, 0.06) |
| Jordan | 1202.46  (1114.22, 1311.45) | -0.22  (-0.32, -0.12) | 0.01  (0.01, 0.01) | -0.61  (-0.72, -0.45) | 30.06  (19.31, 45.21) | -0.32  (-0.43, -0.16) | 29.55  (18.91, 44.70) | -0.31  (-0.43, -0.15) | 0.51  (0.27, 0.71) | -0.63  (-0.77, -0.43) |
| Kuwait | 854.43  (759.66, 1001.17) | -0.25  (-0.36, -0.11) | 0.00  (0.00, 0.00) | -0.63  (-0.72, -0.54) | 18.09  (11.66, 27.92) | -0.22  (-0.36, -0.04) | 18.05  (11.62, 27.86) | -0.22  (-0.36, -0.04) | 0.04  (0.03, 0.06) | -0.65  (-0.74, -0.55) |
| Lebanon | 932.73  (844.77, 1067.71) | -0.33  (-0.43, -0.22) | 0.01  (0.00, 0.01) | -0.21  (-0.50, 0.12) | 24.67  (15.69, 36.33) | -0.36  (-0.49, -0.23) | 24.39  (15.44, 36.03) | -0.37  (-0.49, -0.23) | 0.27  (0.05, 0.39) | -0.17  (-0.58, 0.27) |
| Libya | 1054.99  (940.47, 1190.51) | -0.18  (-0.32, -0.05) | 0.06  (0.02, 0.09) | 0.52  (-0.08, 1.28) | 27.09  (17.65, 40.38) | -0.14  (-0.30, 0.06) | 24.67  (15.93, 37.91) | -0.17  (-0.33, 0.04) | 2.42  (0.53, 3.74) | 0.28  (-0.23, 0.96) |
| Morocco | 1251.66  (1117.67, 1437.12) | -0.25  (-0.36, -0.13) | 0.05  (0.01, 0.07) | -0.10  (-0.41, 0.22) | 32.88  (21.96, 48.29) | -0.30  (-0.42, -0.15) | 30.57  (20.13, 45.80) | -0.31  (-0.43, -0.14) | 2.30  (0.50, 3.61) | -0.22  (-0.63, 0.23) |
| Oman | 1077.07  (894.26, 1367.06) | -0.41  (-0.53, -0.21) | 0.08  (0.05, 0.11) | -0.40  (-0.59, -0.07) | 19.70  (12.96, 29.62) | -0.45  (-0.58, -0.30) | 16.36  (9.59, 25.98) | -0.45  (-0.59, -0.28) | 3.34  (1.95, 4.69) | -0.44  (-0.66, -0.07) |
| Palestine | 922.32  (830.57, 1050.29) | -0.30  (-0.40, -0.16) | 0.00  (0.00, 0.01) | -0.61  (-0.73, -0.34) | 18.18  (11.15, 27.97) | -0.38  (-0.51, -0.20) | 18.02  (10.97, 27.82) | -0.38  (-0.51, -0.20) | 0.16  (0.08, 0.22) | -0.65  (-0.79, -0.31) |
| Qatar | 718.59  (641.99, 827.92) | -0.39  (-0.48, -0.27) | 0.01  (0.00, 0.01) | -0.63  (-0.75, -0.40) | 13.72  (8.47, 20.48) | -0.48  (-0.58, -0.37) | 13.63  (8.38, 20.37) | -0.48  (-0.58, -0.37) | 0.09  (0.01, 0.14) | -0.58  (-0.71, -0.35) |
| Saudi Arabia | 840.76  (756.34, 953.85) | -0.33  (-0.41, -0.23) | 0.09  (0.06, 0.12) | -0.70  (-0.80, -0.42) | 21.58  (14.84, 30.72) | -0.52  (-0.61, -0.41) | 18.73  (12.08, 28.04) | -0.46  (-0.57, -0.33) | 2.85  (2.03, 3.80) | -0.73  (-0.81, -0.50) |
| Sudan | 1594.24  (1424.96, 1831.08) | -0.20  (-0.30, -0.09) | 0.07  (0.02, 0.11) | -0.11  (-0.50, 0.45) | 46.20  (30.82, 70.36) | -0.30  (-0.43, -0.15) | 42.72  (26.94, 66.70) | -0.31  (-0.44, -0.15) | 3.48  (0.68, 5.50) | -0.16  (-0.62, 0.59) |
| Syria | 1270.30  (1129.29, 1477.05) | -0.25  (-0.36, -0.11) | 0.02  (0.01, 0.04) | -0.35  (-0.58, 0.11) | 31.04  (20.11, 44.01) | -0.32  (-0.45, -0.16) | 29.75  (18.82, 42.91) | -0.31  (-0.45, -0.15) | 1.28  (0.44, 2.08) | -0.37  (-0.66, 0.14) |
| Tunisia | 897.47  (819.44, 994.77) | -0.24  (-0.32, -0.14) | 0.03  (0.01, 0.05) | -0.23  (-0.50, 0.10) | 26.32  (17.78, 37.88) | -0.32  (-0.45, -0.18) | 24.85  (16.47, 36.23) | -0.32  (-0.45, -0.16) | 1.47  (0.32, 2.40) | -0.36  (-0.68, 0.07) |
| Türkiye | 974.88  (866.16, 1113.67) | -0.39  (-0.49, -0.27) | 0.07  (0.05, 0.10) | -0.66  (-0.77, -0.46) | 24.23  (16.53, 33.65) | -0.52  (-0.61, -0.41) | 21.41  (13.85, 30.98) | -0.48  (-0.58, -0.36) | 2.83  (1.99, 3.86) | -0.70  (-0.82, -0.47) |
| United Arab Emirates | 1085.42  (922.99, 1273.45) | -0.25  (-0.39, -0.10) | 0.00  (0.00, 0.01) | -0.03  (-0.55, 0.42) | 19.11  (11.57, 29.12) | -0.21  (-0.37, -0.01) | 19.03  (11.57, 29.03) | -0.21  (-0.37, -0.01) | 0.08  (0.00, 0.12) | -0.06  (-0.59, 0.38) |
| Yemen | 2553.20  (2429.00, 2678.26) | 0.07  (0.00, 0.16) | 0.07  (0.02, 0.11) | 0.06  (-0.39, 0.64) | 130.07  (91.64, 177.39) | -0.01  (-0.11, 0.11) | 126.81  (87.86, 174.23) | -0.01  (-0.12, 0.11) | 3.26  (0.67, 4.98) | -0.01  (-0.52, 0.71) |
| **South Asia** |  |  |  |  |  |  |  |  |  |  |
| Bangladesh | 1745.42  (1577.17, 1996.92) | -0.33  (-0.43, -0.22) | 0.17  (0.10, 0.25) | -0.14  (-0.50, 0.26) | 46.82  (32.53, 66.65) | -0.43  (-0.53, -0.32) | 40.22  (26.08, 60.18) | -0.46  (-0.55, -0.35) | 6.60  (4.03, 10.10) | -0.18  (-0.60, 0.34) |
| Bhutan | 2945.27  (2640.47, 3241.58) | 0.15  (-0.08, 0.57) | 0.17  (0.11, 0.28) | 0.05  (-0.28, 0.55) | 101.16  (62.78, 146.51) | 0.01  (-0.33, 0.54) | 94.47  (56.26, 140.72) | 0.01  (-0.33, 0.61) | 6.69  (4.17, 11.52) | 0.05  (-0.35, 0.58) |
| India | 2593.59  (2526.77, 2669.60) | -0.10  (-0.13, -0.07) | 0.18  (0.13, 0.24) | 0.09  (-0.30, 0.48) | 80.86  (57.19, 114.84) | -0.26  (-0.31, -0.20) | 73.98  (49.16, 108.28) | -0.28  (-0.33, -0.23) | 6.87  (5.04, 9.09) | 0.03  (-0.31, 0.45) |
| Nepal | 2342.77  (2050.44, 2714.79) | -0.16  (-0.27, -0.02) | 0.17  (0.10, 0.26) | 0.01  (-0.37, 0.53) | 57.07  (38.36, 84.58) | -0.33  (-0.46, -0.16) | 50.75  (31.95, 77.44) | -0.35  (-0.48, -0.17) | 6.33  (3.85, 10.03) | -0.05  (-0.52, 0.53) |
| Pakistan | 2745.43  (2538.08, 2980.36) | -0.05  (-0.14, 0.07) | 0.21  (0.16, 0.28) | 0.18  (-0.17, 0.67) | 101.89  (70.27, 147.92) | -0.13  (-0.25, 0.02) | 93.44  (61.98, 138.86) | -0.15  (-0.27, 0.01) | 8.45  (6.25, 11.28) | 0.18  (-0.14, 0.67) |
| **South-East Asia, East Asia, and Oceania** | | | | | | | | | | |
| **East Asia** |  |  |  |  |  |  |  |  |  |  |
| China | 392.97  (377.31, 407.34) | -0.56  (-0.57, -0.55) | 0.05  (0.04, 0.06) | -0.53  (-0.64, -0.42) | 11.14  (7.96, 15.30) | -0.62  (-0.65, -0.59) | 8.93  (5.80, 12.99) | -0.61  (-0.63, -0.59) | 2.21  (1.61, 2.79) | -0.63  (-0.73, -0.51) |
| North Korea | 880.85  (794.39, 977.27) | -0.35  (-0.43, -0.26) | 0.10  (0.05, 0.15) | 0.10  (-0.30, 0.70) | 22.87  (15.68, 32.31) | -0.29  (-0.42, -0.12) | 18.35  (11.30, 27.45) | -0.34  (-0.47, -0.16) | 4.52  (2.18, 7.37) | 0.05  (-0.38, 0.64) |
| Taiwan (province of China) | 441.71  (397.19, 490.76) | -0.38  (-0.46, -0.28) | 0.02  (0.02, 0.03) | -0.60  (-0.65, -0.53) | 9.40  (6.06, 14.22) | -0.44  (-0.54, -0.34) | 8.66  (5.37, 13.51) | -0.42  (-0.53, -0.29) | 0.74  (0.64, 0.85) | -0.62  (-0.68, -0.56) |
| **Oceania** |  |  |  |  |  |  |  |  |  |  |
| American Samoa | 1305.20  (1166.04, 1495.83) | -0.08  (-0.20, 0.08) | 0.53  (0.36, 0.71) | 0.08  (-0.21, 0.54) | 38.53  (27.56, 54.08) | -0.06  (-0.21, 0.12) | 30.87  (19.90, 45.47) | -0.07  (-0.24, 0.11) | 7.66  (5.13, 10.62) | 0.00  (-0.28, 0.43) |
| Cook Islands | 1080.39  (937.34, 1275.05) | -0.25  (-0.39, -0.06) | 0.14  (0.04, 0.21) | -0.31  (-0.53, 0.03) | 21.75  (14.35, 32.70) | -0.31  (-0.44, -0.17) | 19.80  (12.58, 30.63) | -0.31  (-0.45, -0.16) | 1.95  (0.52, 2.98) | -0.33  (-0.53, -0.01) |
| Fiji | 1697.36  (1490.72, 1990.53) | 0.06  (-0.09, 0.24) | 0.81  (0.43, 3.49) | -0.16  (-0.41, 0.16) | 53.00  (35.66, 86.72) | -0.13  (-0.27, 0.03) | 35.95  (22.76, 54.71) | -0.08  (-0.24, 0.11) | 17.05  (10.36, 50.45) | -0.21  (-0.45, 0.10) |
| Guam | 1145.59  (1001.05, 1357.77) | -0.08  (-0.28, 0.14) | 0.01  (0.00, 0.01) | -0.59  (-0.74, -0.43) | 20.78  (13.27, 31.08) | -0.08  (-0.26, 0.15) | 20.62  (13.08, 30.92) | -0.08  (-0.26, 0.15) | 0.16  (0.06, 0.22) | -0.51  (-0.66, -0.30) |
| Kiribati | 1931.00  (1712.87, 2181.70) | -0.08  (-0.19, 0.06) | 2.16  (1.27, 2.96) | 0.42  (-0.16, 1.11) | 96.71  (64.67, 131.74) | 0.10  (-0.17, 0.43) | 51.39  (32.73, 77.21) | -0.09  (-0.27, 0.13) | 45.32  (18.47, 65.51) | 0.46  (-0.18, 1.28) |
| Marshall Islands | 1697.39  (1508.34, 1926.13) | -0.03  (-0.17, 0.14) | 0.45  (0.13, 0.69) | 0.05  (-0.30, 0.50) | 51.09  (34.01, 73.65) | -0.02  (-0.20, 0.25) | 44.49  (28.04, 67.34) | -0.02  (-0.24, 0.27) | 6.60  (1.98, 10.02) | 0.02  (-0.32, 0.47) |
| Federated States of Micronesia | 1628.10  (1439.46, 1840.49) | -0.15  (-0.27, -0.01) | 0.47  (0.13, 0.73) | 0.02  (-0.33, 0.49) | 43.88  (31.10, 61.36) | -0.14  (-0.30, 0.03) | 37.19  (23.89, 54.78) | -0.16  (-0.33, 0.04) | 6.69  (1.91, 10.59) | -0.04  (-0.35, 0.40) |
| Nauru | 1698.11  (1482.68, 1979.05) | -0.10  (-0.24, 0.09) | 0.42  (0.11, 0.65) | 0.06  (-0.30, 0.50) | 43.57  (29.26, 61.84) | -0.09  (-0.24, 0.08) | 37.33  (23.91, 55.68) | -0.11  (-0.27, 0.07) | 6.24  (1.72, 9.52) | 0.03  (-0.31, 0.51) |
| Niue | 1381.30  (1194.34, 1592.86) | -0.16  (-0.29, 0.02) | 0.23  (0.06, 0.37) | -0.02  (-0.33, 0.43) | 31.66  (20.57, 46.50) | -0.19  (-0.34, -0.04) | 28.14  (17.47, 42.45) | -0.21  (-0.38, -0.05) | 3.52  (0.96, 5.37) | 0.01  (-0.28, 0.45) |
| Northern Mariana Islands | 1182.60  (1028.47, 1400.46) | -0.10  (-0.28, 0.10) | 0.21  (0.01, 0.31) | 0.07  (-0.25, 0.48) | 24.63  (16.29, 36.04) | -0.06  (-0.22, 0.13) | 22.11  (14.04, 33.16) | -0.06  (-0.23, 0.15) | 2.51  (0.10, 3.53) | -0.03  (-0.30, 0.29) |
| Palau | 1327.72  (1166.65, 1550.45) | -0.18  (-0.31, -0.01) | 0.22  (0.00, 0.35) | -0.20  (-0.52, 0.28) | 30.84  (19.38, 46.28) | -0.21  (-0.35, -0.03) | 27.45  (16.56, 42.51) | -0.20  (-0.36, -0.02) | 3.38  (0.03, 5.49) | -0.24  (-0.56, 0.25) |
| Papua New Guinea | 1768.24  (1483.25, 2129.35) | -0.12  (-0.29, 0.10) | 0.41  (0.11, 0.65) | 0.11  (-0.28, 0.63) | 41.65  (27.26, 66.11) | -0.07  (-0.32, 0.25) | 35.41  (21.08, 58.56) | -0.09  (-0.37, 0.28) | 6.25  (1.80, 9.84) | 0.07  (-0.31, 0.56) |
| Samoa | 1283.57  (1157.63, 1501.40) | -0.12  (-0.26, 0.07) | 0.40  (0.12, 0.61) | 0.02  (-0.31, 0.50) | 38.63  (26.23, 56.23) | -0.16  (-0.31, 0.02) | 32.89  (21.54, 49.69) | -0.18  (-0.33, 0.00) | 5.74  (1.66, 8.6) | -0.02  (-0.31, 0.45) |
| Solomon Islands | 1708.04  (1478.55, 1994.79) | -0.02  (-0.18, 0.20) | 0.46  (0.12, 0.72) | 0.08  (-0.25, 0.64) | 46.86  (32.46, 68.36) | -0.02  (-0.22, 0.23) | 40.03  (26.05, 61.61) | -0.03  (-0.24, 0.27) | 6.83  (1.87, 10.81) | 0.04  (-0.28, 0.55) |
| Tokelau | 1430.08  (1244.18, 1635.81) | -0.20  (-0.34, -0.05) | 0.36  (0.11, 0.53) | -0.19  (-0.42, 0.14) | 36.24  (24.88, 49.48) | -0.24  (-0.37, -0.09) | 30.07  (19.22, 43.43) | -0.27  (-0.40, -0.12) | 6.17  (1.96, 9.22) | -0.08  (-0.34, 0.29) |
| Tonga | 1351.79  (1210.29, 1555.14) | -0.10  (-0.22, 0.07) | 0.24  (0.06, 0.39) | 0.13  (-0.26, 0.66) | 38.65  (23.27, 60.41) | -0.01  (-0.26, 0.32) | 35.4  (21.11, 56.59) | -0.02  (-0.27, 0.35) | 3.25  (0.80, 5.34) | 0.06  (-0.30, 0.54) |
| Tuvalu | 1615.20  (1426.87, 1877.05) | -0.20  (-0.33, -0.05) | 0.47  (0.13, 0.74) | -0.07  (-0.36, 0.38) | 42.61  (29.26, 59.39) | -0.25  (-0.38, -0.09) | 35.91  (23.36, 52.79) | -0.28  (-0.41, -0.11) | 6.70  (1.91, 10.64) | -0.11  (-0.38, 0.29) |
| Vanuatu | 1766.53  (1563.97, 2024.42) | 0.20  (0.01, 0.43) | 0.31  (0.08, 0.49) | 0.12  (-0.23, 0.62) | 47.23  (31.51, 67.39) | 0.26  (0.02, 0.56) | 42.72  (27.25, 63.18) | 0.28  (0.02, 0.61) | 4.51  (1.26, 7.29) | 0.09  (-0.25, 0.55) |
| **Southeast Asia** |  |  |  |  |  |  |  |  |  |  |
| Cambodia | 1761.43  (1544.01, 2067.00) | -0.22  (-0.34, -0.07) | 0.33  (0.12, 0.48) | -0.12  (-0.45, 0.33) | 55.17  (37.82, 77.17) | -0.33  (-0.48, -0.13) | 42.04  (26.73, 62.79) | -0.37  (-0.51, -0.16) | 13.13  (3.77, 19.39) | -0.16  (-0.60, 0.42) |
| Indonesia | 1031.21  (953.52, 1117.17) | -0.37  (-0.44, -0.30) | 0.25  (0.09, 0.34) | -0.02  (-0.30, 0.30) | 30.82  (21.34, 41.52) | -0.28  (-0.42, -0.09) | 20.72  (13.03, 30.54) | -0.34  (-0.46, -0.18) | 10.10  (3.00, 14.3) | -0.10  (-0.51, 0.32) |
| Laos | 1508.94  (1356.34, 1726.05) | -0.19  (-0.31, -0.05) | 0.19  (0.06, 0.30) | -0.21  (-0.54, 0.19) | 39.91  (28.20, 57.99) | -0.33  (-0.49, -0.17) | 31.89  (20.45, 49.74) | -0.36  (-0.49, -0.19) | 8.02  (2.43, 13.88) | -0.21  (-0.64, 0.39) |
| Malaysia | 1490.61  (1275.19, 1733.49) | 0.07  (-0.09, 0.26) | 0.05  (0.04, 0.06) | -0.31  (-0.48, -0.08) | 26.97  (16.80, 38.83) | -0.32  (-0.45, -0.15) | 25.57  (15.54, 37.51) | -0.31  (-0.45, -0.13) | 1.40  (1.07, 1.73) | -0.36  (-0.51, -0.22) |
| Maldives | 1413.65  (1230.74, 1656.45) | -0.37  (-0.47, -0.24) | 0.06  (0.01, 0.09) | -0.48  (-0.66, -0.18) | 37.94  (24.08, 57.25) | -0.52  (-0.62, -0.39) | 36.27  (22.56, 55.65) | -0.52  (-0.63, -0.39) | 1.67  (0.24, 2.45) | -0.48  (-0.76, -0.07) |
| Mauritius | 1299.29  (1085.01, 1694.07) | -0.22  (-0.40, 0.03) | 0.12  (0.10, 0.13) | -0.13  (-0.24, -0.01) | 27.83  (18.66, 41.30) | -0.27  (-0.47, -0.08) | 22.99  (13.77, 36.38) | -0.29  (-0.51, -0.05) | 4.85  (4.11, 5.64) | -0.18  (-0.33, -0.01) |
| Myanmar | 2103.33  (1870.21, 2355.45) | -0.02  (-0.16, 0.17) | 0.18  (0.06, 0.28) | -0.21  (-0.53, 0.17) | 55.20  (37.58, 80.33) | -0.25  (-0.41, -0.03) | 47.04  (28.82, 72.18) | -0.26  (-0.42, -0.02) | 8.16  (2.42, 12.73) | -0.22  (-0.63, 0.35) |
| Philippines | 1038.68  (956.67, 1119.79) | -0.25  (-0.32, -0.16) | 0.31  (0.25, 0.45) | -0.21  (-0.40, -0.01) | 47.27  (34.90, 62.18) | -0.17  (-0.34, -0.02) | 32.74  (21.02, 46.70) | -0.13  (-0.28, 0.04) | 14.53  (10.98, 17.60) | -0.23  (-0.52, 0.03) |
| Seychelles | 1099.30  (967.36, 1284.64) | -0.23  (-0.35, -0.07) | 0.02  (0.01, 0.05) | -0.58  (-0.70, -0.37) | 27.45  (17.16, 42.75) | -0.23  (-0.41, 0.08) | 26.67  (16.49, 41.92) | -0.21  (-0.39, 0.14) | 0.78  (0.50, 1.46) | -0.61  (-0.73, -0.38) |
| Sri Lanka | 1129.06  (1019.74, 1291.82) | -0.40  (-0.49, -0.28) | 0.17  (0.12, 0.24) | -0.50  (-0.65, -0.30) | 32.63  (23.43, 46.52) | -0.37  (-0.47, -0.24) | 27.61  (17.86, 40.94) | -0.33  (-0.45, -0.17) | 5.02  (3.22, 7.33) | -0.52  (-0.68, -0.33) |
| Thailand | 812.81  (730.30, 917.07) | -0.25  (-0.35, -0.14) | 0.09  (0.07, 0.12) | -0.33  (-0.51, -0.03) | 22.74  (15.60, 31.88) | -0.32  (-0.45, -0.19) | 19.59  (12.65, 28.62) | -0.33  (-0.47, -0.17) | 3.15  (2.34, 4.06) | -0.31  (-0.48, -0.03) |
| Timor-Leste | 1387.15  (1212.28, 1630.82) | -0.03  (-0.26, 0.58) | 0.29  (0.10, 0.42) | -0.01  (-0.38, 0.48) | 34.60  (23.03, 48.57) | -0.17  (-0.44, 0.23) | 22.30  (13.67, 34.14) | -0.22  (-0.45, 0.38) | 12.30  (3.51, 17.79) | -0.06  (-0.56, 0.67) |
| Viet Nam | 893.33  (818.84, 979.04) | -0.32  (-0.39, -0.25) | 0.18  (0.07, 0.24) | -0.18  (-0.41, 0.13) | 31.54  (22.34, 44.46) | -0.34  (-0.44, -0.23) | 24.71  (16.22, 36.45) | -0.37  (-0.48, -0.24) | 6.83  (2.10, 10.02) | -0.22  (-0.47, 0.13) |
| **Sub-Saharan Africa** | | | | | | | | | | |
| **Central Sub-Saharan Africa** | |  |  |  |  |  |  |  |  |  |
| Angola | 1370.59  (1192.30, 1577.55) | -0.12  (-0.27, 0.05) | 0.55  (0.14, 0.93) | -0.17  (-0.51, 0.25) | 48.76  (30.84, 68.04) | -0.28  (-0.51, -0.03) | 28.76  (18.49, 44.02) | -0.31  (-0.50, -0.09) | 20.00  (4.64, 32.14) | -0.22  (-0.65, 0.35) |
| Central African Republic | 1702.31  (1427.93, 2005.91) | 0.09  (-0.11, 0.37) | 0.74  (0.21, 1.21) | -0.06  (-0.35, 0.36) | 72.21  (44.62, 100.59) | 0.03  (-0.24, 0.44) | 44.49  (26.57, 70.49) | 0.12  (-0.24, 0.72) | 27.72  (6.89, 43.78) | -0.09  (-0.44, 0.42) |
| Congo (Brazzaville) | 1717.33  (1444.94, 2005.62) | -0.05  (-0.23, 0.17) | 0.55  (0.14, 0.87) | -0.20  (-0.49, 0.15) | 62.82  (42.88, 88.56) | -0.18  (-0.39, 0.09) | 43.68  (26.80, 67.31) | -0.14  (-0.38, 0.18) | 19.14  (4.54, 30.81) | -0.24  (-0.59, 0.15) |
| DR Congo | 1456.96  (1276.75, 1666.22) | -0.33  (-0.43, -0.2) | 0.62  (0.16, 1.07) | -0.05  (-0.41, 0.36) | 54.59  (33.48, 78.59) | -0.33  (-0.52, -0.09) | 33.21  (20.36, 51.42) | -0.41  (-0.60, -0.18) | 21.39  (5.13, 34.37) | -0.14  (-0.56, 0.35) |
| Equatorial Guinea | 1499.49  (1185.89, 1829.47) | -0.29  (-0.45, -0.09) | 0.41  (0.10, 0.74) | -0.42  (-0.64, -0.08) | 47.43  (30.54, 71.29) | -0.44  (-0.61, -0.23) | 33.10  (19.72, 52.90) | -0.44  (-0.64, -0.18) | 14.33  (3.01, 27.15) | -0.45  (-0.72, 0.02) |
| Gabon | 2139.58  (1787.62, 2523.37) | -0.10  (-0.28, 0.12) | 0.47  (0.12, 0.79) | -0.21  (-0.45, 0.10) | 74.24  (48.94, 104.79) | -0.24  (-0.43, -0.01) | 57.81  (36.02, 87.39) | -0.25  (-0.46, 0.02) | 16.43  (3.72, 28.31) | -0.22  (-0.56, 0.22) |
| **Eastern Sub-Saharan Africa** | | | | | | | | | | |
| Burundi | 1597.86  (1337.05, 1862.72) | 0.12  (-0.14, 0.44) | 0.67  (0.16, 1.12) | -0.07  (-0.51, 0.39) | 63.77  (39.12, 92.44) | -0.05  (-0.42, 0.36) | 39.97  (23.59, 65.10) | 0.02  (-0.36, 0.60) | 23.81  (5.51, 39.09) | -0.14  (-0.62, 0.39) |
| Comoros | 1840.20  (1238.86, 2258.56) | -0.16  (-0.47, 0.53) | 0.64  (0.18, 1.07) | -0.06  (-0.41, 0.38) | 69.22  (37.59, 103.76) | -0.09  (-0.45, 0.55) | 44.14  (22.44, 75.56) | -0.10  (-0.51, 1.27) | 25.09  (6.88, 41.73) | -0.08  (-0.51, 0.49) |
| Djibouti | 1751.29  (1427.08, 2103.96) | -0.20  (-0.36, -0.02) | 0.60  (0.17, 1.04) | -0.02  (-0.39, 0.48) | 58.52  (34.94, 85.20) | -0.17  (-0.40, 0.09) | 37.10  (21.39, 59.43) | -0.21  (-0.43, 0.07) | 21.42  (6.01, 37.45) | -0.10  (-0.52, 0.49) |
| Eritrea | 2092.53  (1829.84, 2449.61) | -0.15  (-0.30, 0.01) | 0.49  (0.12, 0.75) | 0.06  (-0.34, 0.50) | 67.23  (46.09, 95.87) | -0.10  (-0.31, 0.17) | 48.20  (30.62, 72.80) | -0.16  (-0.36, 0.13) | 19.03  (5.28, 30.44) | 0.10  (-0.46, 0.74) |
| Ethiopia | 1439.51  (1332.59, 1565.05) | -0.18  (-0.25, -0.10) | 0.62  (0.16, 1.07) | -0.32  (-0.61, 0.01) | 63.22  (42.40, 86.96) | -0.30  (-0.51, -0.13) | 40.13  (26.53, 58.55) | -0.28  (-0.38, -0.17) | 23.09  (6.35, 38.19) | -0.34  (-0.69, 0.05) |
| Kenya | 982.77  (902.84, 1041.52) | -0.05  (-0.14, 0.03) | 0.51  (0.15, 0.84) | 0.04  (-0.21, 0.30) | 41.62  (26.37, 56.32) | -0.07  (-0.26, 0.08) | 23.83  (15.81, 34.35) | -0.11  (-0.19, -0.02) | 17.80  (5.02, 28.12) | -0.02  (-0.40, 0.29) |
| Madagascar | 1765.87  (1598.80, 1999.17) | -0.13  (-0.26, 0.03) | 0.35  (0.09, 0.57) | -0.11  (-0.46, 0.25) | 57.05  (37.95, 82.28) | -0.22  (-0.41, -0.02) | 43.03  (27.26, 64.61) | -0.25  (-0.41, -0.05) | 14.02  (3.69, 22.71) | -0.11  (-0.57, 0.40) |
| Malawi | 2273.21  (1948.08, 2637.01) | -0.17  (-0.31, 0.01) | 0.58  (0.15, 0.95) | -0.05  (-0.49, 0.36) | 71.68  (45.80, 101.68) | -0.41  (-0.58, -0.19) | 49.52  (29.24, 79.00) | -0.49  (-0.64, -0.27) | 22.16  (5.61, 35.15) | -0.13  (-0.64, 0.44) |
| Mozambique | 2197.45  (1988.24, 2460.02) | 0.04  (-0.11, 0.18) | 0.58  (0.15, 0.94) | 0.00  (-0.42, 0.47) | 115.73  (83.81, 156.46) | 0.09  (-0.16, 0.37) | 92.92  (64.98, 132.38) | 0.13  (-0.07, 0.39) | 22.81  (5.65, 36.40) | -0.05  (-0.61, 0.64) |
| Rwanda | 1138.65  (1004.08, 1318.20) | -0.25  (-0.37, -0.10) | 0.60  (0.17, 1.04) | -0.23  (-0.56, 0.19) | 48.92  (29.03, 69.83) | -0.33  (-0.57, -0.09) | 25.80  (16.32, 38.12) | -0.38  (-0.53, -0.20) | 23.12  (5.95, 37.58) | -0.24  (-0.65, 0.33) |
| Somalia | 2055.32  (1788.26, 2340.82) | -0.12  (-0.26, 0.04) | 0.80  (0.21, 1.50) | 0.03  (-0.33, 0.48) | 73.60  (45.45, 104.70) | -0.17  (-0.40, 0.12) | 45.18  (27.04, 70.21) | -0.25  (-0.48, 0.07) | 28.42  (8.37, 51.04) | 0.00  (-0.44, 0.57) |
| South Sudan | 1683.02  (1394.98, 2008.46) | -0.12  (-0.34, 0.12) | 0.66  (0.19, 1.05) | 0.07  (-0.28, 0.57) | 62.12  (37.74, 89.53) | -0.06  (-0.32, 0.29) | 36.40  (21.26, 56.62) | -0.12  (-0.41, 0.36) | 25.72  (6.00, 46.28) | 0.05  (-0.34, 0.63) |
| Uganda | 1267.29  (1081.03, 1505.72) | -0.17  (-0.33, 0.07) | 0.55  (0.14, 0.91) | -0.01  (-0.35, 0.51) | 50.10  (29.97, 74.02) | -0.22  (-0.48, 0.12) | 29.07  (16.86, 47.06) | -0.34  (-0.56, 0.06) | 21.02  (4.92, 33.58) | 0.03  (-0.46, 0.70) |
| Tanzania | 2205.53  (1882.88, 2578.36) | -0.25  (-0.38, -0.11) | 0.59  (0.16, 0.95) | -0.14  (-0.46, 0.22) | 73.88  (47.45, 108.72) | -0.42  (-0.56, -0.24) | 49.51  (30.74, 78.67) | -0.51  (-0.64, -0.32) | 24.37  (6.01, 39.43) | -0.12  (-0.57, 0.37) |
| Zambia | 2680.21  (2368.76, 3019.02) | 0.18  (0.00, 0.40) | 0.54  (0.14, 0.86) | -0.16  (-0.51, 0.21) | 84.43  (59.18, 118.86) | -0.20  (-0.44, 0.07) | 63.23  (38.98, 94.50) | -0.21  (-0.44, 0.09) | 21.21  (5.37, 33.40) | -0.19  (-0.64, 0.37) |
| **Southern Sub-Saharan Africa** | | | | | | | | | | |
| Botswana | 1479.87  (1255.05, 1798.85) | -0.14  (-0.30, 0.08) | 0.44  (0.24, 0.61) | -0.22  (-0.42, 0.11) | 55.20  (36.47, 76.20) | -0.15  (-0.34, 0.13) | 32.58  (19.37, 51.04) | -0.17  (-0.39, 0.19) | 22.63  (11.17, 33.25) | -0.11  (-0.38, 0.33) |
| Eswatini | 1354.20  (1166.59, 1666.97) | -0.09  (-0.27, 0.16) | 0.58  (0.30, 0.87) | 0.02  (-0.33, 0.51) | 61.70  (42.71, 86.64) | -0.02  (-0.27, 0.30) | 32.61  (20.07, 49.94) | -0.11  (-0.34, 0.21) | 29.09  (13.61, 46.02) | 0.10  (-0.34, 0.76) |
| Lesotho | 1382.62  (1244.86, 1629.13) | -0.05  (-0.20, 0.14) | 0.63  (0.33, 0.88) | 0.18  (-0.23, 0.81) | 66.67  (47.32, 88.92) | 0.06  (-0.15, 0.35) | 36.73  (23.73, 53.84) | -0.06  (-0.25, 0.21) | 29.95  (14.04, 46.60) | 0.25  (-0.19, 0.99) |
| Namibia | 1347.90  (1170.03, 1642.10) | -0.40  (-0.52, -0.11) | 0.50  (0.27, 0.74) | -0.03  (-0.34, 0.38) | 53.52  (35.15, 76.59) | -0.21  (-0.40, 0.18) | 29.68  (18.21, 45.91) | -0.34  (-0.54, 0.17) | 23.84  (11.26, 38.83) | 0.04  (-0.36, 0.61) |
| South Africa | 1488.58  (1386.19, 1603.81) | -0.12  (-0.21, -0.02) | 0.27  (0.22, 0.32) | -0.05  (-0.22, 0.12) | 68.38  (50.27, 90.66) | -0.12  (-0.24, 0.01) | 55.65  (38.08, 77.30) | -0.13  (-0.25, 0.02) | 12.73  (9.96, 15.36) | -0.11  (-0.40, 0.14) |
| Zimbabwe | 1695.57  (1522.88, 1928.52) | 0.01  (-0.13, 0.22) | 0.67  (0.33, 0.99) | 0.30  (-0.08, 0.94) | 81.17  (55.06, 110.67) | 0.23  (0.00, 0.56) | 48.61  (32.01, 71.02) | 0.11  (-0.12, 0.44) | 32.56  (14.2, 51.35) | 0.45  (-0.03, 1.24) |
| **Western Sub-Saharan Africa** | | | | | | | | | | |
| Benin | 1993.55  (1766.13, 2264.45) | 0.10  (-0.07, 0.29) | 2.61  (1.49, 4.02) | -0.17  (-0.55, 0.31) | 215.04  (129.99, 322.60) | -0.18  (-0.57, 0.29) | 49.54  (30.45, 74.72) | -0.23  (-0.44, 0.02) | 165.50  (84.73, 261.39) | -0.16  (-0.64, 0.50) |
| Burkina Faso | 2245.94  (1974.99, 2528.34) | 0.31  (0.12, 0.58) | 2.75  (1.87, 4.18) | -0.13  (-0.49, 0.39) | 243.16  (165.42, 337.39) | 0.02  (-0.45, 0.62) | 72.64  (46.57, 108.09) | 0.51  (0.13, 1.11) | 170.52  (104.96, 261.89) | -0.11  (-0.59, 0.63) |
| Cabo Verde | 1757.55  (1522.19, 2093.26) | -0.18  (-0.32, 0.00) | 0.82  (0.31, 1.67) | -0.29  (-0.54, 0.05) | 89.80  (42.41, 141.62) | -0.30  (-0.49, -0.02) | 42.74  (26.27, 68.16) | -0.26  (-0.44, 0.02) | 47.06  (11.85, 93.74) | -0.33  (-0.66, 0.09) |
| Cameroon | 1483.72  (1305.96, 1683.74) | 0.15  (-0.03, 0.36) | 2.37  (1.49, 3.52) | -0.15  (-0.49, 0.34) | 181.87  (117.46, 264.42) | -0.11  (-0.48, 0.40) | 39.46  (25.03, 60.59) | 0.01  (-0.23, 0.34) | 142.41  (77.24, 220.39) | -0.14  (-0.55, 0.48) |
| Chad | 2375.76  (2107.37, 2646.27) | 0.05  (-0.11, 0.23) | 2.74  (1.82, 4.44) | 0.04  (-0.34, 0.63) | 229.10  (161.25, 336.69) | 0.01  (-0.36, 0.56) | 67.65  (43.89, 101.53) | -0.03  (-0.27, 0.30) | 161.45  (101.26, 268.09) | 0.02  (-0.45, 0.83) |
| Côte d'Ivoire | 1967.13  (1652.92, 2257.08) | 0.13  (-0.08, 0.35) | 2.28  (1.32, 3.63) | -0.11  (-0.48, 0.39) | 191.46  (117.28, 291.97) | -0.01  (-0.41, 0.57) | 54.98  (33.92, 82.77) | 0.28  (-0.07, 0.82) | 136.48  (68.51, 221.91) | -0.09  (-0.57, 0.64) |
| The Gambia | 2554.56  (2278.37, 2841.95) | 0.00  (-0.13, 0.13) | 2.47  (1.41, 4.04) | -0.05  (-0.42, 0.45) | 227.39  (139.52, 327.70) | -0.07  (-0.42, 0.31) | 82.48  (53.53, 117.80) | -0.08  (-0.28, 0.15) | 144.91  (66.19, 230.31) | -0.07  (-0.55, 0.63) |
| Ghana | 2206.41  (1927.41, 2503.52) | 0.11  (-0.07, 0.34) | 1.95  (1.19, 3.01) | -0.13  (-0.45, 0.38) | 168.33  (100.30, 245.72) | -0.09  (-0.44, 0.38) | 54.91  (34.06, 82.93) | 0.05  (-0.25, 0.55) | 113.42  (54.26, 182.94) | -0.14  (-0.58, 0.50) |
| Guinea | 1973.57  (1707.30, 2235.35) | 0.17  (-0.02, 0.41) | 2.68  (1.66, 4.20) | -0.16  (-0.58, 0.44) | 217.24  (144.31, 309.45) | -0.13  (-0.58, 0.45) | 55.98  (34.43, 81.84) | 0.21  (-0.11, 0.68) | 161.26  (94.54, 247.57) | -0.21  (-0.67, 0.49) |
| Guinea-Bissau | 2416.86  (2163.57, 2720.10) | 0.02  (-0.13, 0.22) | 2.71  (1.85, 4.36) | -0.24  (-0.57, 0.16) | 219.61  (144.43, 304.31) | -0.23  (-0.58, 0.15) | 69.63  (41.90, 104.15) | -0.06  (-0.29, 0.21) | 149.98  (85.33, 225.69) | -0.29  (-0.69, 0.29) |
| Liberia | 2114.86  (1844.96, 2414.51) | 0.01  (-0.17, 0.23) | 2.38  (1.25, 3.78) | -0.25  (-0.69, 0.39) | 216.56  (130.02, 314.49) | -0.21  (-0.65, 0.34) | 75.36  (50.45, 110.79) | 0.02  (-0.24, 0.35) | 141.20  (63.79, 231.67) | -0.30  (-0.79, 0.49) |
| Mali | 2904.34  (2685.27, 3137.97) | 0.23  (0.09, 0.40) | 2.25  (1.44, 3.54) | -0.23  (-0.57, 0.22) | 226.21  (162.07, 299.80) | -0.10  (-0.48, 0.34) | 100.75  (66.18, 144.78) | 0.30  (0.05, 0.60) | 125.46  (77.47, 189.42) | -0.28  (-0.68, 0.33) |
| Mauritania | 2156.60  (1904.56, 2488.99) | -0.17  (-0.29, -0.02) | 1.20  (0.46, 2.37) | -0.24  (-0.57, 0.15) | 121.81  (64.24, 186.37) | -0.29  (-0.52, 0.00) | 53.61  (33.26, 82.45) | -0.33  (-0.48, -0.15) | 68.20  (20.53, 130.11) | -0.25  (-0.67, 0.28) |
| Niger | 1992.38  (1728.01, 2260.63) | -0.11  (-0.26, 0.08) | 2.33  (1.25, 3.85) | -0.25  (-0.70, 0.31) | 187.05  (112.40, 272.24) | -0.28  (-0.68, 0.20) | 53.26  (31.59, 83.62) | -0.18  (-0.40, 0.14) | 133.79  (67.96, 222.09) | -0.31  (-0.78, 0.37) |
| Nigeria | 2209.86  (2035.50, 2401.82) | 0.05  (-0.08, 0.18) | 2.23  (1.55, 3.07) | -0.09  (-0.49, 0.36) | 204.66  (146.90, 272.28) | -0.06  (-0.45, 0.31) | 64.56  (41.45, 95.71) | -0.06  (-0.22, 0.15) | 140.10  (87.81, 201.86) | -0.06  (-0.56, 0.49) |
| São Tomé and Príncipe | 2012.50  (1741.95, 2371.74) | -0.03  (-0.23, 0.44) | 2.60  (1.44, 3.96) | -0.45  (-0.67, -0.13) | 196.04  (97.62, 311.96) | -0.48  (-0.70, -0.15) | 48.11  (29.37, 77.25) | -0.12  (-0.40, 0.57) | 147.94  (56.46, 254.43) | -0.54  (-0.80, -0.18) |
| Senegal | 2906.19  (2658.10, 3164.37) | 0.00  (-0.11, 0.13) | 1.80  (0.78, 3.16) | -0.20  (-0.59, 0.21) | 188.56  (107.13, 276.84) | -0.25  (-0.53, 0.05) | 84.48  (52.46, 123.59) | -0.24  (-0.42, -0.07) | 104.07  (36.77, 186.12) | -0.26  (-0.71, 0.25) |
| Sierra Leone | 1906.23  (1648.25, 2199.63) | 0.10  (-0.10, 0.37) | 2.56  (1.66, 3.96) | -0.15  (-0.60, 0.45) | 214.15  (140.20, 321.55) | -0.11  (-0.58, 0.56) | 53.50  (31.6, 82.06) | 0.17  (-0.18, 0.73) | 160.66  (95.19, 260.72) | -0.18  (-0.67, 0.65) |
| Togo | 2683.65  (2439.25, 2935.44) | 0.27  (0.08, 0.55) | 2.28  (1.32, 3.43) | -0.13  (-0.49, 0.38) | 210.14  (131.44, 294.22) | -0.02  (-0.38, 0.54) | 79.18  (49.24, 121.71) | 0.36  (-0.01, 0.95) | 130.96  (63.96, 204.43) | -0.16  (-0.61, 0.54) |

Abbreviation: DALYs, disability-adjusted life-years; rIDPs, rare infectious diseases of poverty; UI: uncertainty interval; YLL, years of life lost; YLD, years lived with disability.

**a** percentage change in age-standardized rates between 1990 and 2021;

# Table S14. Temporal trends in age-standardized prevalence, mortality, DALY, YLD, and YLL rates (per 100,000 population) of rIDPs by countries or territories, from 1990 to 2021.

| **Countries** | **Prevalence** | | **Mortality** | | **DALY** | | **YLD** | | **YLL** | |
| --- | --- | --- | --- | --- | --- | --- | --- | --- | --- | --- |
|  | **AAPC, %**  **(95% *CI*)** | ***P* value** | **AAPC, %**  **(95% *CI*)** | ***P* value** | **AAPC, %**  **(95% *CI*)** | ***P* value** | **AAPC, %**  **(95% *CI*)** | ***P* value** | **AAPC, %**  **(95% *CI*)** | ***P* value** |
| **Central Europe, Eastern Europe, and Central Asia** | | | | | | | | | | |
| **Central Europe** |  |  |  |  |  |  |  |  |  |  |
| Albania | -1.77 (-1.86, -1.68) | < 0.001 | -4.91 (-5.34, -4.48) | < 0.001 | -2.84 (-2.97, -2.70) | < 0.001 | -2.16 (-2.24, -2.08) | < 0.001 | -5.42 (-6.00, -4.83) | < 0.001 |
| Bosnia and Herzegovina | -1.01 (-1.08, -0.95) | < 0.001 | -2.61 (-3.12, -2.10) | < 0.001 | -1.70 (-1.88, -1.52) | < 0.001 | -1.69 (-1.82, -1.55) | < 0.001 | -2.98 (-3.39, -2.56) | < 0.001 |
| Bulgaria | -0.61 (-0.64, -0.58) | < 0.001 | 1.09 (-0.19, 2.39) | 0.094 | -0.92 (-1.13, -0.70) | < 0.001 | -1.15 (-1.17, -1.12) | < 0.001 | 0.68 (-0.51, 1.88) | 0.266 |
| Croatia | -1.03 (-1.06, -1.00) | < 0.001 | -5.83 (-9.69, -1.81) | 0.005 | -1.58 (-1.63, -1.52) | < 0.001 | -1.52 (-1.65, -1.39) | < 0.001 | -6.34 (-10.67, -1.79) | 0.007 |
| Czechia | -1.28 (-1.33, -1.23) | < 0.001 | -0.84 (-2.17, 0.51) | 0.221 | -1.84 (-1.93, -1.75) | < 0.001 | -1.83 (-1.89, -1.78) | < 0.001 | -1.89 (-3.28, -0.47) | 0.009 |
| Hungary | -1.19 (-1.22, -1.16) | < 0.001 | 1.13 (-0.62, 2.91) | 0.208 | -1.72 (-1.85, -1.60) | < 0.001 | -1.82 (-1.89, -1.75) | < 0.001 | 0.26 (-1.44, 2.00) | 0.763 |
| Montenegro | -0.48 (-0.53, -0.44) | < 0.001 | -0.36 (-0.90, 0.19) | 0.199 | -0.88 (-0.92, -0.85) | < 0.001 | -0.88 (-0.92, -0.85) | < 0.001 | -0.51 (-1.23, 0.23) | 0.176 |
| North Macedonia | -1.01 (-1.03, -0.99) | < 0.001 | -3.42 (-4.13, -2.70) | < 0.001 | -1.70 (-1.77, -1.63) | < 0.001 | -1.63 (-1.68, -1.58) | < 0.001 | -5.13 (-5.75, -4.50) | < 0.001 |
| Poland | -1.32 (-1.34, -1.30) | < 0.001 | -5.55 (-6.66, -4.43) | < 0.001 | -2.23 (-2.27, -2.19) | < 0.001 | -2.07 (-2.10, -2.03) | < 0.001 | -6.16 (-7.38, -4.93) | < 0.001 |
| Romania | -1.25 (-1.26, -1.24) | < 0.001 | -1.94 (-3.47, -0.38) | 0.015 | -2.10 (-2.16, -2.04) | < 0.001 | -2.06 (-2.08, -2.03) | < 0.001 | -2.77 (-4.25, -1.28) | < 0.001 |
| Serbia | -1.12 (-1.14, -1.09) | < 0.001 | -0.32 (-0.87, 0.23) | 0.250 | -1.64 (-1.69, -1.60) | < 0.001 | -1.69 (-1.74, -1.64) | < 0.001 | -0.45 (-0.78, -0.12) | 0.007 |
| Slovakia | -1.06 (-1.09, -1.04) | < 0.001 | -2.96 (-3.71, -2.21) | < 0.001 | -1.66 (-1.69, -1.64) | < 0.001 | -1.65 (-1.67, -1.63) | < 0.001 | -3.19 (-3.94, -2.43) | < 0.001 |
| Slovenia | -1.41 (-1.45, -1.36) | < 0.001 | -7.13 (-9.28, -4.93) | < 0.001 | -1.96 (-2.02, -1.90) | < 0.001 | -1.88 (-1.92, -1.84) | < 0.001 | -8.14 (-10.87, -5.33) | < 0.001 |
| **Eastern Europe** |  |  |  |  |  |  |  |  |  |  |
| Belarus | -1.04 (-1.08, -1.00) | < 0.001 | -1.23 (-1.49, -0.96) | < 0.001 | -1.39 (-1.46, -1.33) | < 0.001 | -1.41 (-1.46, -1.36) | < 0.001 | -1.32 (-1.63, -1.00) | < 0.001 |
| Estonia | -1.26 (-1.30, -1.23) | < 0.001 | 1.67 (-2.54, 6.07) | 0.442 | -1.69 (-1.78, -1.59) | < 0.001 | -1.68 (-1.76, -1.61) | < 0.001 | -0.10 (-6.74, 7.01) | 0.977 |
| Latvia | -0.86 (-0.90, -0.82) | < 0.001 | -0.56 (-2.13, 1.04) | 0.492 | -1.32 (-1.54, -1.11) | < 0.001 | -1.26 (-1.29, -1.23) | < 0.001 | -1.49 (-3.06, 0.11) | 0.067 |
| Lithuania | -0.78 (-0.79, -0.77) | < 0.001 | 2.64 (0.61, 4.71) | 0.011 | -1.07 (-1.24, -0.90) | < 0.001 | -1.18 (-1.21, -1.15) | < 0.001 | 1.21 (-0.83, 3.30) | 0.248 |
| Moldova | -1.16 (-1.18, -1.14) | < 0.001 | -0.11 (-5.31, 5.38) | 0.969 | -1.34 (-1.41, -1.27) | < 0.001 | -1.36 (-1.40, -1.33) | < 0.001 | 0.19 (-7.46, 8.47) | 0.963 |
| Russia | -0.92 (-0.97, -0.87) | < 0.001 | -1.00 (-1.38, -0.62) | < 0.001 | -1.36 (-1.45, -1.27) | < 0.001 | -1.32 (-1.38, -1.25) | < 0.001 | -1.60 (-1.94, -1.25) | < 0.001 |
| Ukraine | -0.41 (-0.48, -0.35) | < 0.001 | -1.02 (-1.47, -0.56) | < 0.001 | -0.65 (-0.74, -0.56) | < 0.001 | -0.60 (-0.70, -0.50) | < 0.001 | -1.12 (-1.59, -0.64) | < 0.001 |
| **Central Asia** |  |  |  |  |  |  |  |  |  |  |
| Armenia | -0.64 (-0.66, -0.63) | < 0.001 | 0.70 (-0.27, 1.68) | 0.158 | -1.26 (-1.31, -1.22) | < 0.001 | -1.27 (-1.31, -1.24) | < 0.001 | 0.27 (-0.79, 1.34) | 0.616 |
| Azerbaijan | -0.82 (-0.89, -0.75) | < 0.001 | -1.23 (-1.54, -0.91) | < 0.001 | -1.37 (-1.45, -1.28) | < 0.001 | -1.37 (-1.44, -1.29) | < 0.001 | -1.21 (-1.69, -0.72) | < 0.001 |
| Georgia | -0.57 (-0.61, -0.53) | < 0.001 | 5.98 (3.69, 8.33) | < 0.001 | 0.62 (0.48, 0.76) | < 0.001 | -0.64 (-0.73, -0.56) | < 0.001 | 5.26 (2.92, 7.65) | < 0.001 |
| Kazakhstan | -0.90 (-0.94, -0.87) | < 0.001 | 1.08 (0.52, 1.64) | < 0.001 | -1.65 (-1.78, -1.52) | < 0.001 | -1.77 (-1.87, -1.67) | < 0.001 | 0.18 (-0.44, 0.81) | 0.566 |
| Kyrgyzstan | -0.64 (-0.66, -0.62) | < 0.001 | 0.14 (-0.45, 0.74) | 0.639 | -0.86 (-1.05, -0.67) | < 0.001 | -1.04 (-1.06, -1.01) | < 0.001 | -0.24 (-1.07, 0.60) | 0.577 |
| Mongolia | -1.04 (-1.11, -0.97) | < 0.001 | -3.54 (-4.12, -2.97) | < 0.001 | -1.56 (-1.66, -1.46) | < 0.001 | -1.48 (-1.57, -1.39) | < 0.001 | -4.24 (-4.90, -3.57) | < 0.001 |
| Tajikistan | -0.26 (-0.29, -0.23) | < 0.001 | -0.29 (-0.46, -0.11) | 0.001 | -0.55 (-0.59, -0.50) | < 0.001 | -0.58 (-0.61, -0.55) | < 0.001 | -0.14 (-0.36, 0.08) | 0.212 |
| Turkmenistan | -0.71 (-0.72, -0.70) | < 0.001 | -1.23 (-1.44, -1.01) | < 0.001 | -1.23 (-1.28, -1.19) | < 0.001 | -1.24 (-1.28, -1.21) | < 0.001 | -1.10 (-1.35, -0.85) | < 0.001 |
| Uzbekistan | -0.46 (-0.47, -0.45) | < 0.001 | 0.56 (-0.17, 1.30) | 0.131 | -0.57 (-0.60, -0.54) | < 0.001 | -0.63 (-0.68, -0.58) | < 0.001 | 0.66 (-0.09, 1.42) | 0.085 |
| **High income** | | | | | | | | | | |
| **Australasia** |  |  |  |  |  |  |  |  |  |  |
| Australia | -1.07 (-1.10, -1.04) | < 0.001 | -1.50 (-2.31, -0.68) | < 0.001 | -0.97 (-1.17, -0.77) | < 0.001 | -0.84 (-0.93, -0.76) | < 0.001 | -1.72 (-3.24, -0.17) | 0.030 |
| New Zealand | -0.93 (-0.96, -0.91) | < 0.001 | -3.70 (-4.68, -2.71) | < 0.001 | -1.28 (-1.50, -1.06) | < 0.001 | -1.00 (-1.05, -0.94) | < 0.001 | -4.03 (-5.10, -2.94) | < 0.001 |
| **High-income Asia Pacific** | | | | | | | | | | |
| Brunei | -0.90 (-0.93, -0.86) | < 0.001 | -1.76 (-2.06, -1.46) | < 0.001 | -0.83 (-0.90, -0.76) | < 0.001 | -0.79 (-0.86, -0.73) | < 0.001 | -2.13 (-2.39, -1.88) | < 0.001 |
| Japan | -0.87 (-0.92, -0.83) | < 0.001 | -2.57 (-2.82, -2.32) | < 0.001 | -1.10 (-1.20, -1.00) | < 0.001 | -0.81 (-0.88, -0.75) | < 0.001 | -2.82 (-3.08, -2.56) | < 0.001 |
| South Korea | -2.52 (-2.56, -2.48) | < 0.001 | 2.1 (1.71, 2.49) | < 0.001 | -1.35 (-1.44, -1.26) | < 0.001 | -2.25 (-2.36, -2.14) | < 0.001 | 1.48 (1.05, 1.91) | < 0.001 |
| Singapore | -2.42 (-2.51, -2.33) | < 0.001 | -19.81 (-22.48, -17.06) | < 0.001 | -2.76 (-4.23, -1.27) | < 0.001 | -0.88 (-1.33, -0.44) | < 0.001 | -20.09 (-22.85, -17.23) | < 0.001 |
| **High-income North American** | | | | | | | | | | |
| Canada | -0.63 (-0.66, -0.59) | < 0.001 | 0.80 (-1.10, 2.73) | 0.412 | -0.10 (-0.45, 0.25) | 0.579 | -0.30 (-0.39, -0.22) | < 0.001 | 0.57 (-1.30, 2.48) | 0.553 |
| Greenland | -1.38 (-1.44, -1.31) | < 0.001 | 0.19 (-0.38, 0.77) | 0.509 | -0.90 (-1.19, -0.61) | < 0.001 | -1.22 (-1.30, -1.14) | < 0.001 | -0.35 (-1.00, 0.31) | 0.297 |
| USA | -0.13 (-0.19, -0.06) | < 0.001 | 2.84 (1.45, 4.26) | < 0.001 | 0.97 (0.57, 1.38) | < 0.001 | 0.31 (0.22, 0.40) | < 0.001 | 2.42 (1.20, 3.66) | < 0.001 |
| **Western Europe** |  |  |  |  |  |  |  |  |  |  |
| Argentina | -0.94 (-0.99, -0.89) | < 0.001 | 0.98 (0.10, 1.88) | 0.030 | -0.81 (-1.23, -0.39) | < 0.001 | -1.30 (-1.42, -1.17) | < 0.001 | 0.81 (-0.05, 1.68) | 0.066 |
| Chile | -1.83 (-1.84, -1.81) | < 0.001 | -2.03 (-2.47, -1.59) | < 0.001 | -2.06 (-2.32, -1.79) | < 0.001 | -1.89 (-1.94, -1.84) | < 0.001 | -2.36 (-2.81, -1.91) | < 0.001 |
| Uruguay | -1.32 (-1.36, -1.29) | < 0.001 | 9.71 (7.80, 11.66) | < 0.001 | 0.12 (-0.67, 0.90) | 0.773 | -1.41 (-1.48, -1.34) | < 0.001 | 9.07 (6.88, 11.30) | < 0.001 |
| **Southern Latin American** | | | | | | | | | | |
| Andorra | -0.88 (-0.94, -0.81) | < 0.001 | -2.89 (-3.15, -2.63) | < 0.001 | -1.33 (-1.46, -1.19) | < 0.001 | -1.08 (-1.21, -0.95) | < 0.001 | -3.47 (-3.61, -3.34) | < 0.001 |
| Austria | -1.26 (-1.29, -1.23) | < 0.001 | 2.48 (0.26, 4.76) | 0.029 | -1.24 (-1.49, -1.00) | < 0.001 | -1.53 (-1.60, -1.45) | < 0.001 | 1.72 (-0.60, 4.09) | 0.147 |
| Belgium | -1.04 (-1.06, -1.03) | < 0.001 | 0.76 (-0.64, 2.18) | 0.292 | -1.18 (-1.31, -1.05) | < 0.001 | -1.27 (-1.32, -1.23) | < 0.001 | 0.39 (-1.15, 1.94) | 0.624 |
| Cyprus | -1.60 (-1.66, -1.54) | < 0.001 | -4.61 (-6.43, -2.76) | < 0.001 | -2.03 (-2.20, -1.85) | < 0.001 | -1.80 (-1.88, -1.72) | < 0.001 | -4.52 (-5.76, -3.27) | < 0.001 |
| Denmark | -1.23 (-1.26, -1.20) | < 0.001 | 0.80 (-0.64, 2.25) | 0.280 | -1.32 (-1.62, -1.02) | < 0.001 | -1.44 (-1.50, -1.39) | < 0.001 | -0.08 (-1.46, 1.32) | 0.910 |
| Finland | -1.34 (-1.37, -1.32) | < 0.001 | -0.36 (-3.08, 2.44) | 0.800 | -1.52 (-1.80, -1.23) | < 0.001 | -1.56 (-1.66, -1.47) | < 0.001 | -0.99 (-2.71, 0.75) | 0.262 |
| France | -1.27 (-1.31, -1.23) | < 0.001 | -3.47 (-3.98, -2.96) | < 0.001 | -1.90 (-1.98, -1.82) | < 0.001 | -1.55 (-1.63, -1.47) | < 0.001 | -3.92 (-4.43, -3.41) | < 0.001 |
| Germany | -1.51 (-1.54, -1.48) | < 0.001 | 2.31 (1.35, 3.27) | < 0.001 | -1.37 (-1.46, -1.28) | < 0.001 | -1.71 (-1.74, -1.68) | < 0.001 | 1.92 (0.99, 2.85) | < 0.001 |
| Greece | -0.57 (-0.63, -0.52) | < 0.001 | -0.83 (-4.39, 2.86) | 0.653 | -0.86 (-1.08, -0.65) | < 0.001 | -0.79 (-0.85, -0.73) | < 0.001 | -0.94 (-4.55, 2.80) | 0.617 |
| Iceland | -1.08 (-1.14, -1.02) | < 0.001 | 0.88 (-4.46, 6.52) | 0.752 | -1.19 (-1.30, -1.08) | < 0.001 | -1.27 (-1.34, -1.20) | < 0.001 | 0.82 (-4.05, 5.94) | 0.745 |
| Ireland | -1.65 (-1.70, -1.61) | < 0.001 | -2.71 (-4.20, -1.19) | < 0.001 | -2.10 (-2.33, -1.87) | < 0.001 | -2.03 (-2.10, -1.95) | < 0.001 | -2.85 (-4.72, -0.94) | 0.004 |
| Israel | -1.20 (-1.23, -1.18) | < 0.001 | 7.65 (5.91, 9.41) | < 0.001 | -0.60 (-0.94, -0.25) | < 0.001 | -1.47 (-1.52, -1.41) | < 0.001 | 7.24 (5.43, 9.09) | < 0.001 |
| Italy | -1.11 (-1.18, -1.05) | < 0.001 | -1.84 (-2.52, -1.15) | < 0.001 | -1.52 (-1.67, -1.38) | < 0.001 | -1.23 (-1.33, -1.12) | < 0.001 | -2.50 (-3.26, -1.73) | < 0.001 |
| Luxembourg | -1.52 (-1.54, -1.51) | < 0.001 | 3.24 (1.63, 4.87) | < 0.001 | -1.41 (-1.67, -1.14) | < 0.001 | -1.85 (-1.92, -1.77) | < 0.001 | 2.54 (0.29, 4.84) | 0.027 |
| Malta | -1.36 (-1.40, -1.33) | < 0.001 | -8.25 (-10.51, -5.93) | < 0.001 | -2.19 (-2.30, -2.07) | < 0.001 | -1.71 (-1.77, -1.64) | < 0.001 | -8.71 (-10.11, -7.30) | < 0.001 |
| Monaco | -0.78 (-0.85, -0.72) | < 0.001 | 1.18 (0.91, 1.45) | < 0.001 | -0.71 (-0.86, -0.55) | < 0.001 | -0.91 (-0.97, -0.85) | < 0.001 | 0.10 (-0.31, 0.51) | 0.626 |
| Netherlands | -1.03 (-1.06, -1.00) | < 0.001 | 1.08 (-0.12, 2.30) | 0.078 | -1.11 (-1.36, -0.86) | < 0.001 | -1.36 (-1.44, -1.28) | < 0.001 | 0.32 (-0.92, 1.57) | 0.617 |
| Norway | -1.46 (-1.49, -1.43) | < 0.001 | 2.66 (1.33, 3.99) | < 0.001 | -1.33 (-1.56, -1.10) | < 0.001 | -1.72 (-1.77, -1.67) | < 0.001 | 1.98 (0.77, 3.21) | 0.001 |
| Portugal | -1.59 (-1.63, -1.55) | < 0.001 | -4.77 (-5.82, -3.71) | < 0.001 | -2.73 (-3.10, -2.36) | < 0.001 | -1.83 (-1.88, -1.77) | < 0.001 | -5.08 (-6.75, -3.38) | < 0.001 |
| San Marino | -0.64 (-0.73, -0.54) | < 0.001 | -3.03 (-3.53, -2.53) | < 0.001 | -1.21 (-1.40, -1.02) | < 0.001 | -0.87 (-0.98, -0.76) | < 0.001 | -2.85 (-3.84, -1.85) | < 0.001 |
| Spain | -1.38 (-1.39, -1.36) | < 0.001 | -4.94 (-5.62, -4.25) | < 0.001 | -2.38 (-2.49, -2.26) | < 0.001 | -1.54 (-1.61, -1.47) | < 0.001 | -5.72 (-6.57, -4.86) | < 0.001 |
| Sweden | -1.02 (-1.04, -1.00) | < 0.001 | 2.10 (1.01, 3.21) | < 0.001 | -0.93 (-1.03, -0.83) | < 0.001 | -1.26 (-1.31, -1.20) | < 0.001 | 1.34 (0.33, 2.35) | 0.009 |
| Switzerland | -0.84 (-0.89, -0.79) | < 0.001 | 0.32 (-1.86, 2.55) | 0.775 | -1.04 (-1.30, -0.78) | < 0.001 | -1.16 (-1.23, -1.08) | < 0.001 | -0.29 (-2.06, 1.50) | 0.748 |
| UK | -0.64 (-0.66, -0.62) | < 0.001 | -4.97 (-5.51, -4.42) | < 0.001 | -1.13 (-1.18, -1.08) | < 0.001 | -0.78 (-0.82, -0.75) | < 0.001 | -5.51 (-6.08, -4.92) | < 0.001 |
| **Latin America and Caribbean** | | | | | | | | | | |
| **Andean Latin America** |  |  |  |  |  |  |  |  |  |  |
| Bolivia | -1.08 (-1.11, -1.05) | < 0.001 | -2.15 (-2.17, -2.13) | < 0.001 | -1.68 (-1.70, -1.66) | < 0.001 | -1.47 (-1.50, -1.44) | < 0.001 | -2.55 (-2.59, -2.51) | < 0.001 |
| Ecuador | -1.95 (-2.04, -1.87) | < 0.001 | -4.14 (-5.01, -3.26) | < 0.001 | -3.37 (-3.88, -2.86) | < 0.001 | -1.85 (-1.93, -1.76) | < 0.001 | -4.70 (-5.57, -3.82) | < 0.001 |
| Peru | -1.73 (-1.81, -1.64) | < 0.001 | -2.44 (-3.02, -1.86) | < 0.001 | -2.53 (-2.70, -2.37) | < 0.001 | -2.36 (-2.40, -2.32) | < 0.001 | -2.99 (-3.60, -2.38) | < 0.001 |
| **Central Latin America** |  |  |  |  |  |  |  |  |  |  |
| Colombia | -1.81 (-1.86, -1.76) | < 0.001 | -5.06 (-5.74, -4.37) | < 0.001 | -3.28 (-3.71, -2.86) | < 0.001 | -1.53 (-1.63, -1.44) | < 0.001 | -5.22 (-5.95, -4.48) | < 0.001 |
| Costa Rica | -0.80 (-0.81, -0.79) | < 0.001 | -4.76 (-9.00, -0.31) | 0.036 | -1.63 (-2.08, -1.17) | < 0.001 | -0.69 (-0.79, -0.60) | < 0.001 | -5.02 (-9.56, -0.24) | 0.040 |
| El Salvador | -0.81 (-0.86, -0.77) | < 0.001 | -0.69 (-1.35, -0.03) | 0.041 | -1.44 (-1.56, -1.31) | < 0.001 | -1.45 (-1.51, -1.39) | < 0.001 | -1.41 (-1.67, -1.14) | < 0.001 |
| Guatemala | -0.59 (-0.60, -0.57) | < 0.001 | -6.70 (-7.79, -5.59) | < 0.001 | -4.68 (-5.05, -4.32) | < 0.001 | -1.02 (-1.05, -0.99) | < 0.001 | -7.12 (-8.41, -5.80) | < 0.001 |
| Honduras | -0.59 (-0.60, -0.58) | < 0.001 | -1.98 (-2.22, -1.75) | < 0.001 | -1.53 (-1.68, -1.39) | < 0.001 | -0.91 (-0.96, -0.86) | < 0.001 | -2.04 (-2.31, -1.78) | < 0.001 |
| Mexico | -0.87 (-0.89, -0.84) | < 0.001 | -4.80 (-5.23, -4.37) | < 0.001 | -3.68 (-3.96, -3.40) | < 0.001 | -1.17 (-1.27, -1.07) | < 0.001 | -5.35 (-5.81, -4.88) | < 0.001 |
| Nicaragua | -0.89 (-0.90, -0.88) | < 0.001 | -5.38 (-5.67, -5.09) | < 0.001 | -2.63 (-2.75, -2.52) | < 0.001 | -0.89 (-0.91, -0.87) | < 0.001 | -5.84 (-6.15, -5.54) | < 0.001 |
| Panama | -0.98 (-1.02, -0.95) | < 0.001 | -3.02 (-4.64, -1.38) | < 0.001 | -2.28 (-2.97, -1.59) | < 0.001 | -1.30 (-1.38, -1.22) | < 0.001 | -3.25 (-4.91, -1.56) | < 0.001 |
| Venezuela | -0.97 (-1.02, -0.92) | < 0.001 | -2.66 (-3.88, -1.42) | < 0.001 | -1.87 (-2.16, -1.57) | < 0.001 | -1.10 (-1.17, -1.02) | < 0.001 | -2.76 (-4.01, -1.50) | < 0.001 |
| **Tropical Latin America** | | | | | | | | | | |
| Brazil | -0.85 (-0.87, -0.82) | < 0.001 | -1.47 (-1.79, -1.16) | < 0.001 | -1.39 (-1.54, -1.23) | < 0.001 | -1.01 (-1.03, -0.98) | < 0.001 | -2.35 (-2.69, -2.00) | < 0.001 |
| Paraguay | -0.72 (-0.74, -0.69) | < 0.001 | -0.24 (-0.64, 0.16) | < 0.001 | -0.85 (-0.96, -0.73) | < 0.001 | -0.91 (-0.96, -0.86) | < 0.001 | -0.43 (-0.91, 0.06) | 0.083 |
| **Caribbean** |  |  |  |  |  |  |  |  |  |  |
| Antigua and Barbuda | -0.74 (-0.78, -0.70) | < 0.001 | 8.45 (2.80, 14.42) | 0.003 | -0.42 (-0.70, -0.14) | 0.003 | -1.03 (-1.09, -0.98) | < 0.001 | 8.56 (2.39, 15.1) | 0.006 |
| Bahamas | -0.46 (-0.51, -0.41) | < 0.001 | 10.44 (3.79, 17.51) | 0.002 | -0.46 (-0.68, -0.25) | < 0.001 | -0.68 (-0.72, -0.64) | < 0.001 | 10.45 (5.92, 15.18) | < 0.001 |
| Barbados | -0.60 (-0.62, -0.58) | < 0.001 | 12.20 (9.51, 14.96) | < 0.001 | 0.20 (-0.09, 0.48) | 0.176 | -0.77 (-0.81, -0.73) | < 0.001 | 11.75 (8.98, 14.58) | < 0.001 |
| Belize | -0.26 (-0.29, -0.24) | < 0.001 | 8.82 (6.48, 11.20) | < 0.001 | -0.20 (-0.48, 0.07) | 0.152 | -0.58 (-0.61, -0.55) | < 0.001 | 7.33 (5.04, 9.66) | < 0.001 |
| Bermuda | -1.38 (-1.42, -1.34) | < 0.001 | -2.81 (-3.50, -2.12) | < 0.001 | -1.75 (-1.83, -1.67) | < 0.001 | -1.74 (-1.81, -1.66) | < 0.001 | -2.94 (-3.96, -1.91) | < 0.001 |
| Cuba | -0.50 (-0.54, -0.45) | < 0.001 | -2.72 (-3.71, -1.73) | < 0.001 | -0.92 (-1.02, -0.82) | < 0.001 | -0.74 (-0.77, -0.72) | < 0.001 | -2.95 (-3.96, -1.93) | < 0.001 |
| Dominica | -0.47(-0.48, -0.45) | < 0.001 | 5.39 (4.17, 6.61) | < 0.001 | -0.49 (-0.54, -0.44) | < 0.001 | -0.59 (-0.61, -0.57) | < 0.001 | 5.06 (3.52, 6.62) | < 0.001 |
| Dominican Republic | -0.83 (-0.92, -0.74) | < 0.001 | -2.17 (-2.54, -1.81) | < 0.001 | -1.34 (-1.40, -1.29) | < 0.001 | -1.14 (-1.19, -1.08) | < 0.001 | -2.45 (-3.00, -1.89) | < 0.001 |
| Grenada | -0.55 (-0.57, -0.53) | < 0.001 | -0.08 (-4.77, 4.84) | 0.973 | -0.92 (-1.41, -0.43) | < 0.001 | -1.09 (-1.12, -1.05) | < 0.001 | -0.50 (-4.25, 3.40) | 0.799 |
| Guyana | -0.86 (-0.89, -0.83) | < 0.001 | 1.87 (0.75, 2.99) | 0.001 | 0.24 (-0.20, 0.68) | 0.290 | -0.85 (-0.93, -0.78) | < 0.001 | 1.76 (0.68, 2.85) | 0.001 |
| Haiti | 0.15 (0.11, 0.18) | < 0.001 | 0.53 (0.41, 0.65) | < 0.001 | -0.75 (-0.77, -0.72) | < 0.001 | -0.79 (-0.82, -0.77) | < 0.001 | 0.35 (0.20, 0.50) | < 0.001 |
| Jamaica | -0.33 (-0.35, -0.31) | < 0.001 | 8.22 (6.38, 10.1) | < 0.001 | 0.53 (0.13, 0.93) | 0.010 | -0.65 (-0.69, -0.62) | < 0.001 | 7.31 (5.63, 9.01) | < 0.001 |
| Puerto Rico | -1.03 (-1.06, -1.00) | < 0.001 | 3.72 (1.56, 5.92) | < 0.001 | -0.99 (-1.24, -0.73) | < 0.001 | -1.29 (-1.34, -1.25) | < 0.001 | 3.43 (1.93, 4.96) | < 0.001 |
| Saint Kitts and Nevis | -1.02 (-1.05, -1.00) | < 0.001 | 7.02 (4.50, 9.61) | < 0.001 | -1.51 (-1.76, -1.26) | < 0.001 | -1.61 (-1.65, -1.57) | < 0.001 | 5.69 (3.30, 8.14) | < 0.001 |
| Saint Lucia | -0.71 (-0.72, -0.69) | < 0.001 | 5.98 (3.05, 8.99) | < 0.001 | -0.17 (-0.54, 0.20) | 0.376 | -1.13 (-1.17, -1.09) | < 0.001 | 6.12 (2.67, 9.68) | < 0.001 |
| Saint Vincent and the Grenadines | -0.37 (-0.38, -0.35) | < 0.001 | -2.50 (-4.86, -0.09) | 0.042 | -1.29 (-2.17, -0.41) | 0.004 | -0.76 (-0.79, -0.72) | < 0.001 | -3.43 (-6.01, -0.78) | 0.012 |
| Suriname | -0.35 (-0.37, -0.32) | < 0.001 | 1.73 (0.88, 2.60) | < 0.001 | -0.45 (-0.61, -0.30) | < 0.001 | -0.72 (-0.75, -0.70) | < 0.001 | 1.84 (0.92, 2.76) | < 0.001 |
| Trinidad and Tobago | -0.75 (-0.77, -0.73) | < 0.001 | 3.42 (1.86, 5.00) | < 0.001 | -0.73 (-0.95, -0.50) | < 0.001 | -1.30 (-1.34, -1.27) | < 0.001 | 3.66 (2.13, 5.20) | < 0.001 |
| Virgin Islands | -0.60 (-0.62, -0.58) | < 0.001 | -2.31 (-3.47, -1.13) | < 0.001 | -0.95 (-1.03, -0.87) | < 0.001 | -0.93 (-1.02, -0.85) | < 0.001 | -2.79 (-4.01, -1.56) | < 0.001 |
| **North Africa and Middle East** | | | | | | | | | | |
| **North Africa and Middle East** | | | | | | | | | | |
| Afghanistan | -0.94 (-0.96, -0.91) | < 0.001 | 0.01 (-0.17, 0.19) | 0.936 | -1.23 (-1.30, -1.16) | < 0.001 | -1.36 (-1.42, -1.3) | < 0.001 | -0.08 (-0.33, 0.17) | 0.528 |
| Algeria | -1.04 (-1.06, -1.02) | < 0.001 | -0.68 (-0.76, -0.61) | < 0.001 | -1.35 (-1.37, -1.33) | < 0.001 | -1.35 (-1.38, -1.33) | < 0.001 | -1.30 (-1.40, -1.20) | < 0.001 |
| Bahrain | -1.49 (-1.54, -1.43) | < 0.001 | -0.26 (-1.32, 0.80) | 0.629 | -1.82 (-1.90, -1.73) | < 0.001 | -1.85 (-1.90, -1.8) | < 0.001 | -1.00 (-2.31, 0.33) | 0.138 |
| Egypt | -1.08 (-1.15, -1.02) | < 0.001 | 5.03 (4.55, 5.50) | < 0.001 | -0.98 (-1.08, -0.88) | < 0.001 | -1.65 (-1.73, -1.56) | < 0.001 | 4.84 (4.33, 5.36) | < 0.001 |
| Iran | -1.29 (-1.34, -1.24) | < 0.001 | -1.38 (-1.68, -1.08) | < 0.001 | -1.52 (-1.58, -1.46) | < 0.001 | -1.44 (-1.49, -1.39) | < 0.001 | -2.01 (-2.43, -1.60) | < 0.001 |
| Iraq | -1.05 (-1.09, -1.01) | < 0.001 | -1.50 (-1.65, -1.36) | < 0.001 | -1.42 (-1.48, -1.36) | < 0.001 | -1.42 (-1.48, -1.36) | < 0.001 | -1.64 (-1.92, -1.36) | < 0.001 |
| Jordan | -0.80 (-0.84, -0.77) | < 0.001 | -2.93 (-3.48, -2.39) | < 0.001 | -1.23 (-1.27, -1.19) | < 0.001 | -1.18 (-1.21, -1.14) | < 0.001 | -3.19 (-3.65, -2.73) | < 0.001 |
| Kuwait | -0.96 (-1.02, -0.89) | < 0.001 | -2.55 (-8.63, 3.94) | 0.433 | -0.81 (-1.03, -0.59) | < 0.001 | -0.80 (-0.95, -0.66) | < 0.001 | -2.53 (-8.68, 4.04) | 0.441 |
| Lebanon | -1.29 (-1.32, -1.27) | < 0.001 | -0.69 (-0.97, -0.42) | < 0.001 | -1.46 (-1.50, -1.41) | < 0.001 | -1.47 (-1.52, -1.42) | < 0.001 | -0.64 (-1.06, -0.21) | 0.004 |
| Libya | -0.64 (-0.73, -0.54) | < 0.001 | 1.33 (1.09, 1.56) | < 0.001 | -0.47 (-0.57, -0.37) | < 0.001 | -0.57 (-0.69, -0.45) | < 0.001 | 0.78 (0.56, 1.00) | < 0.001 |
| Morocco | -0.93 (-0.94, -0.92) | < 0.001 | -0.31 (-0.51, -0.11) | 0.003 | -1.16 (-1.20, -1.13) | < 0.001 | -1.19 (-1.23, -1.15) | < 0.001 | -0.76 (-0.93, -0.59) | < 0.001 |
| Oman | -1.68 (-1.71, -1.64) | < 0.001 | -1.60 (-1.94, -1.25) | < 0.001 | -1.92 (-2.08, -1.75) | < 0.001 | -1.90 (-2.02, -1.79) | < 0.001 | -1.88 (-2.36, -1.39) | < 0.001 |
| Palestine | -1.15 (-1.19, -1.12) | < 0.001 | -2.99 (-3.41, -2.56) | < 0.001 | -1.55 (-1.59, -1.51) | < 0.001 | -1.53 (-1.57, -1.49) | < 0.001 | -3.29 (-3.78, -2.79) | < 0.001 |
| Qatar | -1.61 (-1.64, -1.57) | < 0.001 | -3.15 (-4.14, -2.14) | < 0.001 | -2.09 (-2.18, -2.00) | < 0.001 | -2.09 (-2.17, -2.00) | < 0.001 | -2.70 (-3.71, -1.68) | < 0.001 |
| Saudi Arabia | -1.13 (-1.20, -1.06) | < 0.001 | -3.82 (-4.05, -3.58) | < 0.001 | -2.28 (-2.46, -2.11) | < 0.001 | -1.90 (-2.01, -1.79) | < 0.001 | -4.12 (-4.29, -3.95) | < 0.001 |
| Sudan | -0.71 (-0.74, -0.67) | < 0.001 | -0.37 (-0.45, -0.28) | < 0.001 | -1.12 (-1.16, -1.08) | < 0.001 | -1.16 (-1.21, -1.12) | < 0.001 | -0.55 (-0.68, -0.41) | < 0.001 |
| Syria | -0.92 (-0.97, -0.87) | < 0.001 | -1.35 (-1.67, -1.02) | < 0.001 | -1.12 (-1.16, -1.08) | < 0.001 | -1.24 (-1.29, -1.19) | < 0.001 | -1.47 (-1.89, -1.05) | < 0.001 |
| Tunisia | -0.86 (-0.89, -0.83) | < 0.001 | -0.86 (-0.97, -0.75) | < 0.001 | -1.25 (-1.29, -1.20) | < 0.001 | -1.24 (-1.28, -1.19) | < 0.001 | -1.42 (-1.57, -1.27) | < 0.001 |
| Türkiye | -1.54 (-1.60, -1.47) | < 0.001 | -3.42 (-3.61, -3.22) | < 0.001 | -2.33 (-2.39, -2.27) | < 0.001 | -2.06 (-2.13, -1.98) | < 0.001 | -3.86 (-4.09, -3.62) | < 0.001 |
| United Arab Emirates | -0.95 (-1.06, -0.83) | < 0.001 | -0.10 (-2.42, 2.28) | 0.936 | -0.77 (-0.84, -0.70) | < 0.001 | -0.77 (-0.82, -0.73) | < 0.001 | -0.18 (-2.33, 2.02) | 0.875 |
| Yemen | 0.22 (0.21, 0.23) | < 0.001 | 0.19 (0.03, 0.35) | 0.017 | -0.02 (-0.04, 0.00) | 0.038 | -0.02 (-0.04, 0.00) | 0.022 | 0.00 (-0.19, 0.18) | 0.960 |
| **South Asia** | | | | | | | | | | |
| **South Asia** | | | | | | | | | | |
| Bangladesh | -1.31 (-1.35, -1.28) | < 0.001 | -0.46 (-0.66, -0.26) | < 0.001 | -1.81 (-1.85, -1.77) | < 0.001 | -1.97 (-2.00, -1.94) | < 0.001 | -0.62 (-0.76, -0.47) | < 0.001 |
| Bhutan | 0.45 (0.38, 0.51) | < 0.001 | 0.19 (-0.01, 0.39) | 0.057 | 0.01 (-0.10, 0.12) | 0.799 | 0.00 (-0.11, 0.12) | 0.974 | 0.21 (-0.11, 0.53) | 0.199 |
| India | -0.34 (-0.35, -0.34) | < 0.001 | 0.27 (-0.19, 0.73) | 0.250 | -0.96 (-0.99, -0.94) | < 0.001 | -1.05 (-1.06, -1.04) | < 0.001 | 0.14 (-0.12, 0.41) | 0.285 |
| Nepal | -0.55 (-0.66, -0.44) | < 0.001 | 0.03 (-0.05, 0.11) | 0.437 | -1.26 (-1.34, -1.17) | < 0.001 | -1.38 (-1.47, -1.29) | < 0.001 | -0.17 (-0.30, -0.04) | 0.011 |
| Pakistan | -0.17 (-0.19, -0.15) | < 0.001 | 0.58 (0.41, 0.75) | < 0.001 | -0.46 (-0.51, -0.42) | < 0.001 | -0.54 (-0.59, -0.50) | < 0.001 | 0.51 (0.14, 0.88) | 0.007 |
| **South-East Asia, East Asia, and Oceania** | | | | | | | | | | |
| **East Asia** | | | | | | | | | | |
| China | -2.59 (-2.64, -2.55) | < 0.001 | -2.49 (-2.92, -2.07) | < 0.001 | -3.04 (-3.17, -2.90) | < 0.001 | -3.01 (-3.06, -2.96) | < 0.001 | -3.23 (-3.69, -2.76) | < 0.001 |
| North Korea | -1.36 (-1.40, -1.33) | < 0.001 | 0.31 (0.25, 0.36) | < 0.001 | -1.09 (-1.13, -1.04) | < 0.001 | -1.33 (-1.40, -1.27) | < 0.001 | 0.15 (0.08, 0.22) | < 0.001 |
| Taiwan (province of China) | -1.51 (-1.54, -1.48) | < 0.001 | -2.83 (-4.08, -1.56) | < 0.001 | -1.85 (-1.93, -1.77) | < 0.001 | -1.72 (-1.78, -1.67) | < 0.001 | -3.02 (-4.11, -1.91) | < 0.001 |
| **Oceania** | | | | | | | | | | |
| American Samoa | -0.25 (-0.26, -0.25) | < 0.001 | 0.27 (-0.71, 1.26) | 0.590 | -0.21 (-0.41, 0.00) | 0.049 | -0.23 (-0.27, -0.19) | < 0.001 | -0.11 (-1.25, 1.04) | 0.849 |
| Cook Islands | -0.93 (-0.95, -0.91) | < 0.001 | -1.20 (-1.27, -1.14) | < 0.001 | -1.21 (-1.24, -1.18) | < 0.001 | -1.20 (-1.23, -1.17) | < 0.001 | -1.26 (-1.36, -1.16) | < 0.001 |
| Fiji | 0.19 (0.17, 0.21) | < 0.001 | -0.55 (-0.83, -0.27) | < 0.001 | -0.42 (-0.59, -0.25) | < 0.001 | -0.28 (-0.31, -0.25) | < 0.001 | -0.74 (-0.95, -0.53) | < 0.001 |
| Guam | -0.29 (-0.33, -0.24) | < 0.001 | -3.04 (-3.86, -2.23) | < 0.001 | -0.30 (-0.38, -0.22) | < 0.001 | -0.27 (-0.37, -0.17) | < 0.001 | -2.50 (-3.43, -1.56) | < 0.001 |
| Kiribati | -0.28 (-0.29, -0.27) | < 0.001 | 1.15 (1.04, 1.27) | < 0.001 | 0.32 (0.25, 0.39) | < 0.001 | -0.32 (-0.36, -0.28) | < 0.001 | 1.26 (1.04, 1.49) | < 0.001 |
| Marshall Islands | -0.11 (-0.12, -0.10) | < 0.001 | 0.14 (0.04, 0.24) | 0.005 | -0.05 (-0.08, -0.03) | < 0.001 | -0.07 (-0.11, -0.03) | 0.001 | 0.05 (0.00, 0.11) | 0.058 |
| Federated States of Micronesia | -0.53 (-0.55, -0.51) | < 0.001 | 0.06 (0.03, 0.09) | < 0.001 | -0.49 (-0.51, -0.46) | < 0.001 | -0.55 (-0.58, -0.53) | < 0.001 | -0.11 (-0.15, -0.08) | < 0.001 |
| Nauru | -0.34 (-0.36, -0.32) | < 0.001 | 0.18 (0.14, 0.22) | < 0.001 | -0.31 (-0.35, -0.27) | < 0.001 | -0.38 (-0.42, -0.35) | < 0.001 | 0.09 (0.04, 0.13) | < 0.001 |
| Niue | -0.56 (-0.57, -0.55) | < 0.001 | -0.07 (-0.20, 0.07) | 0.330 | -0.69 (-0.76, -0.62) | < 0.001 | -0.77 (-0.79, -0.74) | < 0.001 | 0.09 (0.03, 0.16) | 0.006 |
| Northern Mariana Islands | -0.37 (-0.49, -0.24) | < 0.001 | 0.11 (-0.77, 1.00) | 0.808 | -0.25 (-0.39, -0.11) | < 0.001 | -0.23 (-0.36, -0.10) | 0.001 | -0.21 (-0.90, 0.49) | 0.555 |
| Palau | -0.64 (-0.67, -0.60) | < 0.001 | -0.75 (-0.86, -0.64) | < 0.001 | -0.75 (-0.78, -0.71) | < 0.001 | -0.73 (-0.78, -0.68) | < 0.001 | -0.90 (-1.02, -0.78) | < 0.001 |
| Papua New Guinea | -0.40 (-0.46, -0.35) | < 0.001 | 0.33 (0.21, 0.45) | < 0.001 | -0.19 (-0.24, -0.15) | < 0.001 | -0.27 (-0.31, -0.22) | < 0.001 | 0.24 (0.14, 0.34) | < 0.001 |
| Samoa | -0.40 (-0.42, -0.38) | < 0.001 | 0.06 (0.04, 0.09) | < 0.001 | -0.54 (-0.57, -0.52) | < 0.001 | -0.62 (-0.65, -0.59) | < 0.001 | -0.05 (-0.08, -0.02) | 0.001 |
| Solomon Islands | -0.06 (-0.08, -0.05) | < 0.001 | 0.25 (0.13, 0.38) | < 0.001 | -0.06 (-0.09, -0.03) | < 0.001 | -0.09 (-0.12, -0.06) | < 0.001 | 0.14 (-0.01, 0.28) | 0.070 |
| Tokelau | -0.71 (-0.76, -0.67) | < 0.001 | -0.66 (-0.73, -0.58) | < 0.001 | -0.86 (-0.91, -0.82) | < 0.001 | -1.01 (-1.07, -0.94) | < 0.001 | -0.10 (-0.31, 0.11) | 0.340 |
| Tonga | -0.34 (-0.35, -0.33) | < 0.001 | 0.39 (0.29, 0.48) | < 0.001 | 0.03 (-0.04, 0.09) | 0.445 | 0.01 (-0.09, 0.10) | 0.853 | 0.17 (0.05, 0.29) | 0.007 |
| Tuvalu | -0.73 (-0.74, -0.72) | < 0.001 | -0.22 (-0.30, -0.14) | < 0.001 | -0.94 (-0.96, -0.92) | < 0.001 | -1.03 (-1.05, -1.01) | < 0.001 | -0.38 (-0.49, -0.27) | < 0.001 |
| Vanuatu | 0.58 (0.54, 0.62) | < 0.001 | 0.35 (0.29, 0.42) | < 0.001 | 0.74 (0.68, 0.79) | < 0.001 | 0.79 (0.73, 0.85) | < 0.001 | 0.27 (0.14, 0.39) | < 0.001 |
| **Southeast Asia** |  |  |  |  |  |  |  |  |  |  |
| Cambodia | -0.79 (-0.83, -0.75) | < 0.001 | -0.40 (-0.46, -0.34) | < 0.001 | -1.27 (-1.33, -1.21) | < 0.001 | -1.46 (-1.52, -1.41) | < 0.001 | -0.55 (-0.65, -0.45) | < 0.001 |
| Indonesia | -1.51 (-1.57, -1.45) | < 0.001 | -0.06 (-0.13, 0.01) | 0.073 | -1.05 (-1.07, -1.02) | < 0.001 | -1.36 (-1.41, -1.30) | < 0.001 | -0.33 (-0.42, -0.25) | < 0.001 |
| Laos | -0.69 (-0.72, -0.66) | < 0.001 | -0.75 (-0.83, -0.67) | < 0.001 | -1.30 (-1.34, -1.27) | < 0.001 | -1.43 (-1.49, -1.36) | < 0.001 | -0.79 (-0.91, -0.66) | < 0.001 |
| Malaysia | 0.22 (0.12, 0.32) | < 0.001 | -1.31 (-1.63, -0.99) | < 0.001 | -1.22 (-1.28, -1.16) | < 0.001 | -1.20 (-1.23, -1.18) | < 0.001 | -1.42 (-2.34, -0.50) | 0.003 |
| Maldives | -1.47 (-1.54, -1.41) | < 0.001 | -2.22 (-2.82, -1.63) | < 0.001 | -2.32 (-2.41, -2.24) | < 0.001 | -2.33 (-2.42, -2.25) | < 0.001 | -2.16 (-2.57, -1.75) | < 0.001 |
| Mauritius | -0.80 (-0.82, -0.79) | < 0.001 | -0.19 (-1.47, 1.11) | 0.776 | -0.96 (-1.27, -0.65) | < 0.001 | -1.10 (-1.14, -1.07) | < 0.001 | -0.72 (-1.94, 0.52) | 0.254 |
| Myanmar | -0.05 (-0.07, -0.02) | < 0.001 | -0.76 (-0.85, -0.66) | < 0.001 | -0.92 (-0.98, -0.86) | < 0.001 | -0.94 (-1.01, -0.88) | < 0.001 | -0.79 (-0.90, -0.68) | < 0.001 |
| Philippines | -0.90 (-0.92, -0.88) | < 0.001 | -0.76 (-0.91, -0.60) | < 0.001 | -0.58 (-0.67, -0.50) | < 0.001 | -0.44 (-0.47, -0.42) | < 0.001 | -0.86 (-1.21, -0.51) | < 0.001 |
| Seychelles | -0.82 (-0.84, -0.80) | < 0.001 | -2.78 (-2.95, -2.61) | < 0.001 | -0.83 (-0.89, -0.77) | < 0.001 | -0.75 (-0.78, -0.71) | < 0.001 | -2.92 (-3.25, -2.59) | < 0.001 |
| Sri Lanka | -1.64 (-1.68, -1.59) | < 0.001 | -2.25 (-2.67, -1.82) | < 0.001 | -1.46 (-1.52, -1.39) | < 0.001 | -1.25 (-1.28, -1.23) | < 0.001 | -2.37 (-2.89, -1.85) | < 0.001 |
| Thailand | -0.93 (-0.96, -0.91) | < 0.001 | -1.31 (-1.68, -0.94) | < 0.001 | -1.26 (-1.31, -1.20) | < 0.001 | -1.27 (-1.32, -1.22) | < 0.001 | -1.15 (-1.43, -0.87) | < 0.001 |
| Timor-Leste | -0.12 (-0.22, -0.03) | 0.01 | -0.05 (-0.21, 0.12) | 0.587 | -0.62 (-0.74, -0.51) | 0.01 | -0.82 (-0.96, -0.68) | < 0.001 | -0.20 (-0.50, 0.10) | 0.191 |
| Viet Nam | -1.26 (-1.28, -1.24) | < 0.001 | -0.63 (-0.70, -0.55) | < 0.001 | -1.37 (-1.41, -1.32) | < 0.001 | -1.50 (-1.54, -1.46) | < 0.001 | -0.80 (-0.95, -0.64) | < 0.001 |
| **Sub-Saharan Africa** | | | | | | | | | | |
| **Central sub-Saharan Africa** | | | | | | | | | | |
| Angola | -0.41 (-0.48, -0.34) | < 0.001 | -0.53 (-0.71, -0.36) | < 0.001 | -1.05 (-1.18, -0.93) | < 0.001 | -1.21 (-1.27, -1.14) | < 0.001 | -0.78 (-0.97, -0.58) | < 0.001 |
| Central African Republic | 0.30 (0.26, 0.35) | < 0.001 | -0.21 (-0.28, -0.14) | < 0.001 | 0.10 (0.01, 0.19) | < 0.001 | 0.39 (0.30, 0.48) | < 0.001 | -0.32 (-0.42, -0.21) | < 0.001 |
| Congo (Brazzaville) | -0.16 (-0.21, -0.10) | < 0.001 | -0.68 (-0.85, -0.51) | < 0.001 | -0.62 (-0.77, -0.46) | < 0.001 | -0.48 (-0.55, -0.40) | < 0.001 | -0.86 (-1.08, -0.65) | < 0.001 |
| DR Congo | -1.27 (-1.32, -1.22) | < 0.001 | -0.16 (-0.25, -0.08) | < 0.001 | -1.31 (-1.42, -1.19) | < 0.001 | -1.70 (-1.80, -1.61) | < 0.001 | -0.50 (-0.64, -0.35) | < 0.001 |
| Equatorial Guinea | -1.09 (-1.27, -0.92) | < 0.001 | -1.75 (-1.87, -1.64) | < 0.001 | -1.88 (-2.06, -1.69) | < 0.001 | -1.85 (-2.01, -1.69) | < 0.001 | -1.90 (-2.17, -1.62) | < 0.001 |
| Gabon | -0.30 (-0.37, -0.24) | < 0.001 | -0.70 (-0.95, -0.44) | < 0.001 | -0.88 (-0.97, -0.79) | < 0.001 | -0.88 (-0.96, -0.81) | < 0.001 | -0.75 (-1.05, -0.45) | < 0.001 |
| **Eastern sub-Saharan Africa** | | | | | | | | | | |
| Burundi | 0.38 (0.33, 0.43) | < 0.001 | -0.23 (-0.40, -0.05) | 0.011 | -0.15 (-0.34, 0.04) | 0.121 | 0.07 (-0.19, 0.33) | 0.599 | -0.53 (-0.72, -0.34) | < 0.001 |
| Comoros | -0.65 (-0.92, -0.37) | < 0.001 | -0.23 (-0.80, 0.35) | 0.433 | -0.38 (-0.81, 0.06) | 0.089 | -0.35 (-0.67, -0.02) | 0.035 | -0.36 (-1.25, 0.54) | 0.435 |
| Djibouti | -0.71 (-0.74, -0.67) | < 0.001 | 0.05 (-0.11, 0.20) | 0.559 | -0.50 (-0.61, -0.39) | < 0.001 | -0.73 (-0.77, -0.70) | < 0.001 | -0.09 (-0.57, 0.39) | 0.704 |
| Eritrea | -0.54 (-0.56, -0.52) | < 0.001 | 0.21 (0.08, 0.34) | 0.001 | -0.34 (-0.38, -0.29) | < 0.001 | -0.57 (-0.59, -0.55) | < 0.001 | 0.33 (0.18, 0.48) | < 0.001 |
| Ethiopia | -0.64 (-0.68, -0.61) | < 0.001 | -1.22 (-1.27, -1.18) | < 0.001 | -1.16 (-1.22, -1.10) | < 0.001 | -1.07 (-1.10, -1.04) | < 0.001 | -1.31 (-1.46, -1.16) | < 0.001 |
| Kenya | -0.17 (-0.24, -0.11) | < 0.001 | 0.13 (0.00, 0.26) | 0.052 | -0.23 (-0.38, -0.08) | 0.002 | -0.38 (-0.44, -0.32) | < 0.001 | -0.04 (-0.25, 0.18) | 0.744 |
| Madagascar | -0.45 (-0.47, -0.44) | < 0.001 | -0.37 (-0.47, -0.27) | < 0.001 | -0.82 (-0.88, -0.75) | < 0.001 | -0.94 (-1.02, -0.86) | < 0.001 | -0.38 (-0.47, -0.28) | < 0.001 |
| Malawi | -0.57 (-0.63, -0.52) | < 0.001 | -0.14 (-0.30, 0.02) | 0.089 | -1.68 (-1.82, -1.54) | < 0.001 | -2.12 (-2.28, -1.96) | < 0.001 | -0.41 (-0.73, -0.08) | 0.015 |
| Mozambique | 0.12 (0.05, 0.20) | < 0.001 | 0.03 (-0.13, 0.20) | 0.689 | 0.27 (0.20, 0.34) | < 0.001 | 0.39 (0.34, 0.43) | < 0.001 | -0.16 (-0.51, 0.19) | 0.368 |
| Rwanda | -0.94 (-1.01, -0.87) | < 0.001 | -0.80 (-0.96, -0.63) | < 0.001 | -1.24 (-1.55, -0.92) | < 0.001 | -1.55 (-1.61, -1.50) | < 0.001 | -0.81 (-1.48, -0.14) | 0.017 |
| Somalia | -0.39 (-0.44, -0.35) | < 0.001 | 0.12 (0.00, 0.24) | 0.047 | -0.59 (-0.72, -0.46) | < 0.001 | -0.91 (-0.99, -0.83) | < 0.001 | 0.04 (-0.14, 0.22) | 0.651 |
| South Sudan | -0.42 (-0.45, -0.40) | < 0.001 | 0.23 (0.06, 0.40) | 0.006 | -0.19 (-0.30, -0.08) | 0.001 | -0.40 (-0.46, -0.34) | < 0.001 | 0.18 (-0.08, 0.44) | 0.176 |
| Uganda | -0.63 (-0.71, -0.55) | < 0.001 | -0.04 (-0.25, 0.17) | 0.698 | -0.82 (-0.93, -0.70) | < 0.001 | -1.33 (-1.45, -1.22) | < 0.001 | 0.06 (-0.34, 0.46) | 0.787 |
| Tanzania | -0.94 (-1.01, -0.88) | < 0.001 | -0.47 (-0.52, -0.41) | < 0.001 | -1.76 (-1.88, -1.64) | < 0.001 | -2.23 (-2.35, -2.10) | < 0.001 | -0.43 (-0.55, -0.31) | < 0.001 |
| Zambia | 0.56 (0.47, 0.64) | < 0.001 | -0.54 (-0.67, -0.41) | < 0.001 | -0.70 (-0.78, -0.62) | < 0.001 | -0.74 (-0.79, -0.68) | < 0.001 | -0.66 (-0.86, -0.45) | < 0.001 |
| **Southern sub-Saharan Africa** | | | | | | | | | | |
| Botswana | -0.46 (-0.54, -0.39) | < 0.001 | -0.76 (-1.11, -0.41) | < 0.001 | -0.52 (-0.61, -0.42) | < 0.001 | -0.60 (-0.66, -0.54) | < 0.001 | -0.40 (-0.62, -0.18) | < 0.001 |
| Eswatini | -0.29 (-0.32, -0.26) | < 0.001 | 0.07 (-0.11, 0.26) | 0.427 | -0.05 (-0.11, 0.00) | 0.056 | -0.38 (-0.42, -0.34) | < 0.001 | 0.33 (0.13, 0.53) | 0.001 |
| Lesotho | -0.16 (-0.20, -0.12) | < 0.001 | 0.57 (0.44, 0.70) | < 0.001 | 0.21 (0.11, 0.30) | < 0.001 | -0.18 (-0.25, -0.12) | < 0.001 | 0.74 (0.56, 0.93) | < 0.001 |
| Namibia | -1.65 (-1.74, -1.56) | < 0.001 | -0.06 (-0.12, 0.00) | 0.035 | -0.76 (-0.85, -0.66) | < 0.001 | -1.33 (-1.39, -1.26) | < 0.001 | 0.15 (-0.15, 0.45) | 0.339 |
| South Africa | -0.42 (-0.44, -0.40) | < 0.001 | -0.16 (-0.36, 0.04) | 0.116 | -0.43 (-0.47, -0.38) | < 0.001 | -0.44 (-0.46, -0.42) | < 0.001 | -0.39 (-0.60, -0.17) | < 0.001 |
| Zimbabwe | 0.05 (-0.01, 0.11) | 0.083 | 0.88 (0.64, 1.13) | < 0.001 | 0.62 (0.46, 0.79) | < 0.001 | 0.34 (0.29, 0.39) | < 0.001 | 1.09 (0.66, 1.52) | < 0.001 |
| **Western sub-Saharan Africa** | | | | | | | | | | |
| Benin | 0.31 (0.27, 0.35) | < 0.001 | -0.59 (-0.89, -0.29) | < 0.001 | -0.67 (-0.91, -0.44) | < 0.001 | -0.81 (-0.86, -0.75) | < 0.001 | -0.53 (-0.99, -0.07) | 0.024 |
| Burkina Faso | 0.92 (0.79, 1.06) | < 0.001 | -0.38 (-0.52, -0.23) | < 0.001 | 0.12 (-0.04, 0.29) | 0.137 | 1.38 (1.30, 1.47) | < 0.001 | -0.33 (-0.55, -0.11) | 0.003 |
| Cabo Verde | -0.64 (-0.68, -0.61) | < 0.001 | -1.10 (-1.36, -0.85) | < 0.001 | -1.16 (-1.34, -0.98) | < 0.001 | -1.00 (-1.03, -0.96) | < 0.001 | -1.23 (-1.74, -0.71) | < 0.001 |
| Cameroon | 0.47 (0.42, 0.52) | < 0.001 | -0.49 (-0.76, -0.22) | < 0.001 | -0.35 (-0.54, -0.16) | < 0.001 | 0.04 (-0.04, 0.11) | 0.360 | -0.47 (-0.94, 0.01) | 0.053 |
| Chad | 0.16 (0.13, 0.18) | < 0.001 | 0.13 (-0.12, 0.39) | 0.312 | 0.04 (-0.14, 0.21) | 0.693 | -0.08 (-0.12, -0.04) | < 0.001 | 0.12 (-0.22, 0.47) | 0.480 |
| Côte d'Ivoire | 0.37 (0.32, 0.42) | < 0.001 | -0.39 (-0.64, -0.13) | 0.004 | -0.03 (-0.27, 0.20) | 0.777 | 0.80 (0.67, 0.93) | < 0.001 | -0.32 (-0.60, -0.03) | 0.031 |
| The Gambia | -0.02 (-0.08, 0.03) | 0.450 | -0.17 (-1.24, 0.91) | 0.759 | -0.29 (-1.11, 0.54) | 0.496 | -0.29 (-0.39, -0.19) | < 0.001 | -0.23 (-1.40, 0.96) | 0.706 |
| Ghana | 0.35 (0.28, 0.42) | < 0.001 | -0.43 (-0.62, -0.24) | < 0.001 | -0.26 (-0.51, -0.02) | 0.036 | 0.17 (0.00, 0.34) | 0.051 | -0.47 (-0.74, -0.19) | 0.001 |
| Guinea | 0.53 (0.46, 0.60) | < 0.001 | -0.52 (-0.82, -0.21) | 0.001 | -0.39 (-0.72, -0.07) | 0.017 | 0.65 (0.54, 0.77) | < 0.001 | -0.70 (-1.11, -0.29) | 0.001 |
| Guinea-Bissau | 0.08 (0.05, 0.12) | < 0.001 | -0.87 (-1.37, -0.36) | 0.001 | -0.77 (-1.58, 0.04) | 0.063 | -0.20 (-0.25, -0.15) | < 0.001 | -1.01 (-2.07, 0.05) | 0.062 |
| Liberia | 0.00 (-0.06, 0.07) | 0.902 | -1.15 (-1.54, -0.76) | < 0.001 | -1.01 (-1.35, -0.67) | < 0.001 | 0.05 (-0.07, 0.16) | 0.439 | -1.36 (-1.86, -0.85) | < 0.001 |
| Mali | 0.70 (0.65, 0.76) | < 0.001 | -0.78 (-1.20, -0.37) | < 0.001 | -0.39 (-0.84, 0.07) | 0.093 | 0.88 (0.80, 0.97) | < 0.001 | -0.99 (-1.62, -0.35) | 0.002 |
| Mauritania | -0.59 (-0.61, -0.57) | < 0.001 | -0.97 (-1.14, -0.80) | < 0.001 | -1.10 (-1.24, -0.97) | < 0.001 | -1.28 (-1.31, -1.24) | < 0.001 | -1.02 (-1.25, -0.79) | < 0.001 |
| Niger | -0.37 (-0.43, -0.32) | < 0.001 | -0.95 (-1.22, -0.68) | < 0.001 | -1.06 (-1.30, -0.82) | < 0.001 | -0.62 (-0.67, -0.57) | < 0.001 | -1.26 (-1.61, -0.91) | < 0.001 |
| Nigeria | 0.15 (0.13, 0.18) | < 0.001 | -0.32 (-0.39, -0.25) | < 0.001 | -0.17 (-0.28, -0.06) | 0.003 | -0.20 (-0.25, -0.15) | < 0.001 | -0.17 (-0.28, -0.06) | 0.002 |
| São Tomé and Príncipe | -0.12 (-0.29, 0.06) | 0.192 | -1.90 (-2.73, -1.07) | < 0.001 | -2.12 (-3.03, -1.21) | < 0.001 | -0.45 (-0.64, -0.25) | < 0.001 | -2.55 (-3.67, -1.42) | < 0.001 |
| Senegal | 0.00 (-0.03, 0.03) | 0.943 | -0.78 (-1.39, -0.16) | 0.013 | -0.97 (-1.56, -0.37) | 0.002 | -0.90 (-0.94, -0.85) | < 0.001 | -1.02 (-2.06, 0.04) | 0.059 |
| Sierra Leone | 0.30 (0.24, 0.36) | < 0.001 | -0.44 (-1.07, 0.19) | 0.170 | -0.38 (-1.02, 0.26) | 0.244 | 0.52 (0.42, 0.63) | < 0.001 | -0.56 (-1.38, 0.27) | 0.184 |
| Togo | 0.77 (0.72, 0.81) | < 0.001 | -0.45 (-0.72, -0.17) | 0.001 | -0.01 (-0.41, 0.39) | 0.963 | 1.00 (0.92, 1.08) | < 0.001 | -0.51 (-0.92, -0.10) | 0.016 |

Abbreviation: APC, annual percentage change; AAPC, average annual percentage change; *CI*, Confidence interval; DALYs, disability-adjusted life-years; rIDPs, rare infectious diseases of poverty; SDI, social-demographic index; YLL, years of life lost; YLD, years lived with disability.

**Note:** Temporal trends in age-standardized prevalence, mortality, DALY, YLD, and YLL rates for rIDPs were analyzed by the Joinpoint Regression Program (Version 5.2.0, National Cancer Institute: Rockville, MD, United States).

# Table S15. Predicted age-standardized prevalence, mortality, and YLL rates (per 100,000 population) of rIDPs from 2022-2050 with standard deviation, by sex, SDI levels, GBD super regions and sub-regions, based on the Bayesian age-period-cohort model

| **Groups** | **Prevalence** | | **Mortality** | | **YLL** | |
| --- | --- | --- | --- | --- | --- | --- |
|  | **2050**  **ASR (95% *CI*)** | **2022–2050**  **EAPC^a^ (95% *CI*)** | **2050**  **ASR (95% *CI*)** | **2022–2050**  **EAPC^a^ (95% *CI*)** | **2050**  **ASR (95% *CI*)** | **2022–2050**  **EAPC^a^ (95% *CI*)** |
| Global | 1205.17 (682.39, 1727.95) | -0.32 (-0.33, -0.32) | 0.30 (0.14, 0.46) | 0.35 (0.34, 0.36) | 20.09 (3.22, 36.96) | 0.34 (0.33, 0.34) |
| Male | 811.67 (331.42, 1291.91) | -0.40 (-0.42, -0.39) | 0.38 (0.20, 0.57) | 0.57 (0.55, 0.58) | 25.05 (5.31, 44.78) | 0.58 (0.58, 0.58) |
| Female | 1581.03 (1052.21, 2109.86) | -0.35 (-0.36, -0.34) | 0.25 (0.11, 0.38) | 0.32 (0.31, 0.32) | 15.97 (2.05, 29.90) | 0.17 (0.17, 0.18) |
| Low SDI | 2189.82 (1480.38, 2899.26) | -1.09 (-1.10, -1.08) | 1.04 (0.69, 1.38) | -0.79 (-0.80, -0.78) | 53.17 (27.14, 79.2) | -1.04 (-1.05, -1.04) |
| Low-middle SDI | 1163.52 (738.02, 1589.02) | -0.74 (-0.74, -0.74) | 0.25 (0.14, 0.36) | -0.01 (-0.01, 0.00) | 13.64 (6.37, 20.92) | -0.05 (-0.05, -0.05) |
| Middle SDI | 1335.35 (765.66, 1905.04) | -1.09 (-1.09, -1.09) | 0.17 (0.11, 0.23) | -1.56 (-1.56, -1.55) | 7.38 (3.74, 11.01) | -1.99 (-1.99, -1.99) |
| High-middle SDI | 384.74 (8.96, 778.44) | -0.26 (-0.30, -0.21) | 0.02 (0.01, 0.03) | -2.18 (-2.21, -2.15) | 0.64 (0.27, 1.00) | -2.81 (-2.84, -2.78) |
| High SDI | 228.21 (147.32, 309.10) | -1.47 (-1.49, -1.45) | 0.04 (0.02, 0.05) | 0.15 (0.13, 0.18) | 1.11 (0.57, 1.65) | -0.59 (-0.61, -0.56) |
| Central Europe, Eastern Europe, and Central Asia | 946.23 (528.55, 1363.91) | -0.36 (-0.38, -0.35) | 0.04 (0.02, 0.05) | -1.02 (-1.04, -1.01) | 1.56 (0.80, 2.32) | -1.83 (-1.85, -1.82) |
| Central Asia | 1455.49 (356.5, 2554.49) | -0.42 (-0.43, -0.42) | 0.10 (0.05, 0.16) | 1.14 (1.10, 1.18) | 4.16 (1.57, 6.76) | -0.17 (-0.19, -0.15) |
| Central Europe | 609.21 (327.24, 891.18) | -1.25 (-1.25, -1.25) | 0.00 (0.00, 0.01) | -4.68 (-4.82, -4.53) | 0.25 (0.08, 0.42) | -3.45 (-3.51, -3.39) |
| Eastern Europe | 522.98 (290.05, 755.91) | -1.29 (-1.30, -1.27) | 0.03 (0.02, 0.04) | -2.04 (-2.07, -2.01) | 1.18 (0.46, 1.91) | -2.74 (-2.76, -2.71) |
| High-income | 206.43 (129.10, 283.77) | -1.30 (-1.31, -1.28) | 0.04 (0.02, 0.05) | 0.27 (0.25, 0.28) | 1.16 (0.60, 1.72) | -0.54 (-0.55, -0.53) |
| Australasia | 228.98 (144.25, 313.71) | -0.77 (-0.78, -0.77) | 0.14 (0.02, 0.78) | 19.20 (19.08, 19.32) | 0.14 (0.06, 0.34) | -2.69 (-2.70, -2.68) |
| High-income Asia Pacific | 205.33 (129.81, 280.85) | -1.77 (-1.79, -1.75) | 0.01 (0.01, 0.01) | -2.17 (-2.3, -2.04) | 0.50 (0.04, 1.04) | -1.21 (-1.24, -1.19) |
| High-income North America | 212.78 (135.48, 290.08) | -0.67 (-0.69, -0.65) | 0.14 (0.02, 0.30) | 2.98 (2.97, 2.99) | 4.03 (0.22, 7.84) | 1.80 (1.79, 1.81) |
| Southern Latin America | 467.99 (112.62, 823.35) | -0.39 (-0.40, -0.38) | 0.04 (0.03, 0.12) | 0.55 (0.53, 0.57) | 1.59 (0.05, 3.23) | -0.64 (-0.66, -0.62) |
| Western Europe | 195.69 (114.29, 277.08) | -1.21 (-1.22, -1.20) | 0.01 (0.00, 0.01) | -3.37 (-3.46, -3.29) | 0.23 (0.11, 0.35) | -3.35 (-3.38, -3.32) |
| Latin America and Caribbean | 869.32 (469.98, 1268.65) | -0.33 (-0.34, -0.31) | 0.07 (0.02, 0.12) | -3.09 (-3.10, -3.08) | 2.44 (0.94, 3.93) | -4.36 (-4.38, -4.34) |
| Andean Latin America | 1014.00 (214.97, 1813.04) | 0.28 (0.23, 0.32) | 0.05 (0.01, 0.10) | -2.78 (-2.80, -2.76) | 2.12 (0.72, 3.52) | -3.68 (-3.69, -3.66) |
| Caribbean | 2001.71 (993.93, 3009.48) | 0.39 (0.39, 0.39) | 0.10 (0.05, 0.16) | 0.64 (0.62, 0.67) | 4.86 (1.02, 8.70) | 0.26 (0.25, 0.28) |
| Central Latin America | 417.19 (188.23, 646.15) | -1.10 (-1.10, -1.09) | 0.03 (0.00, 0.08) | -4.90 (-4.91, -4.90) | 1.36 (0.07, 2.79) | -5.98 (-5.99, -5.97) |
| Tropical Latin America | 1355.03 (817.66, 1892.40) | 0.25 (0.23, 0.28) | 0.17 (0.07, 0.26) | -1.06 (-1.08, -1.03) | 5.12 (2.30, 7.94) | -2.72 (-2.78, -2.66) |
| North Africa and Middle East | 992.53 (644.63, 1340.43) | -1.52 (-1.52, -1.51) | 0.05 (0.03, 0.07) | -1.95 (-1.97, -1.93) | 1.83 (1.11, 2.54) | -2.89 (-2.89, -2.88) |
| North Africa and Middle East | 992.53 (644.63, 1340.43) | -1.52 (-1.52, -1.51) | 0.05 (0.03, 0.07) | -1.95 (-1.97, -1.93) | 1.83 (1.11, 2.54) | -2.89 (-2.89, -2.88) |
| South Asia | 2072.27 (1160.50, 2984.03) | -0.54 (-0.54, -0.53) | 0.19 (0.08, 0.30) | 0.15 (0.15, 0.16) | 7.75 (2.42, 13.09) | 0.45 (0.44, 0.47) |
| South Asia | 2072.23 (1160.27, 2984.19) | -0.54 (-0.54, -0.53) | 0.19 (0.08, 0.30) | 0.15 (0.15, 0.16) | 7.75 (2.43, 13.08) | 0.45 (0.44, 0.47) |
| Southeast Asia, East Asia, and Oceania | 546.58 (94.47, 998.69) | -0.67 (-0.71, -0.63) | 0.06 (0.03, 0.09) | -1.29 (-1.30, -1.28) | 2.79 (0.75, 4.83) | -1.44 (-1.45, -1.43) |
| East Asia | 219.54 (64.89, 374.19) | -2.07 (-2.11, -2.03) | 0.02 (0.00, 0.04) | -2.75 (-2.79, -2.71) | 0.72 (0.05, 1.39) | -3.52 (-3.56, -3.49) |
| Oceania | 2227.24 (988.75, 3465.74) | 0.57 (0.54, 0.61) | 0.39 (0.25, 1.04) | 0.83 (0.82, 0.84) | 3.75 (2.80, 4.69) | -2.04 (-2.11, -1.98) |
| Southeast Asia | 844.91 (487.78, 1202.04) | -0.92 (-0.93, -0.91) | 0.16 (0.12, 0.21) | -0.60 (-0.60, -0.60) | 7.67 (4.83, 10.52) | -0.41 (-0.41, -0.40) |
| Sub-Saharan Africa | 1508.69 (1049.35, 1968.03) | -0.51 (-0.52, -0.51) | 0.97 (0.66, 1.28) | -0.73 (-0.73, -0.72) | 51.74 (28.62, 74.85) | -0.96 (-0.97, -0.96) |
| Central sub-Saharan Africa | 688.98 (464.98, 912.99) | -2.26 (-2.31, -2.22) | 0.43 (0.34, 0.51) | -0.31 (-0.32, -0.30) | 11.27 (9.14, 13.41) | -1.54 (-1.56, -1.53) |
| Eastern sub-Saharan Africa | 1087.84 (616.41, 1559.26) | -1.16 (-1.17, -1.15) | 0.37 (0.30, 0.44) | -1.06 (-1.07, -1.05) | 12.63 (9.28, 15.98) | -1.57 (-1.57, -1.56) |
| Southern sub-Saharan Africa | 1193.04 (247.80, 2138.28) | -0.42 (-0.42, -0.41) | 0.39 (0.15, 0.62) | 0.70 (0.69, 0.70) | 16.12 (1.03, 31.21) | 0.01 (0.00, 0.01) |
| Western sub-Saharan Africa | 2498.19 (1618.33, 3378.05) | 0.43 (0.42, 0.43) | 2.01 (1.23, 2.80) | -0.51 (-0.51, -0.50) | 120.05 (59.55, 180.55) | -0.53 (-0.53, -0.52) |

Abbreviation: ASR, age-standardized rate; *CI*, confidence interval; DALYs, disability-adjusted life-years; EAPC, estimated annual percentage change; GBD, global burden of disease; rIDPs, rare infectious diseases of poverty; SDI, socio-demographic index; YLD, years lived with disability.

**a** EAPC was used to quantify the estimated changing trends from 2022 to 2050. A linear regression model was fitted based on the natural logarithm of the ASR (*y = α + βx + ε*), where *y* was equal to logarithmically transformed ASR. EAPC was calculated as 100 × (*e^β^* − 1), and 95% *CI* was obtained from the linear regression model.


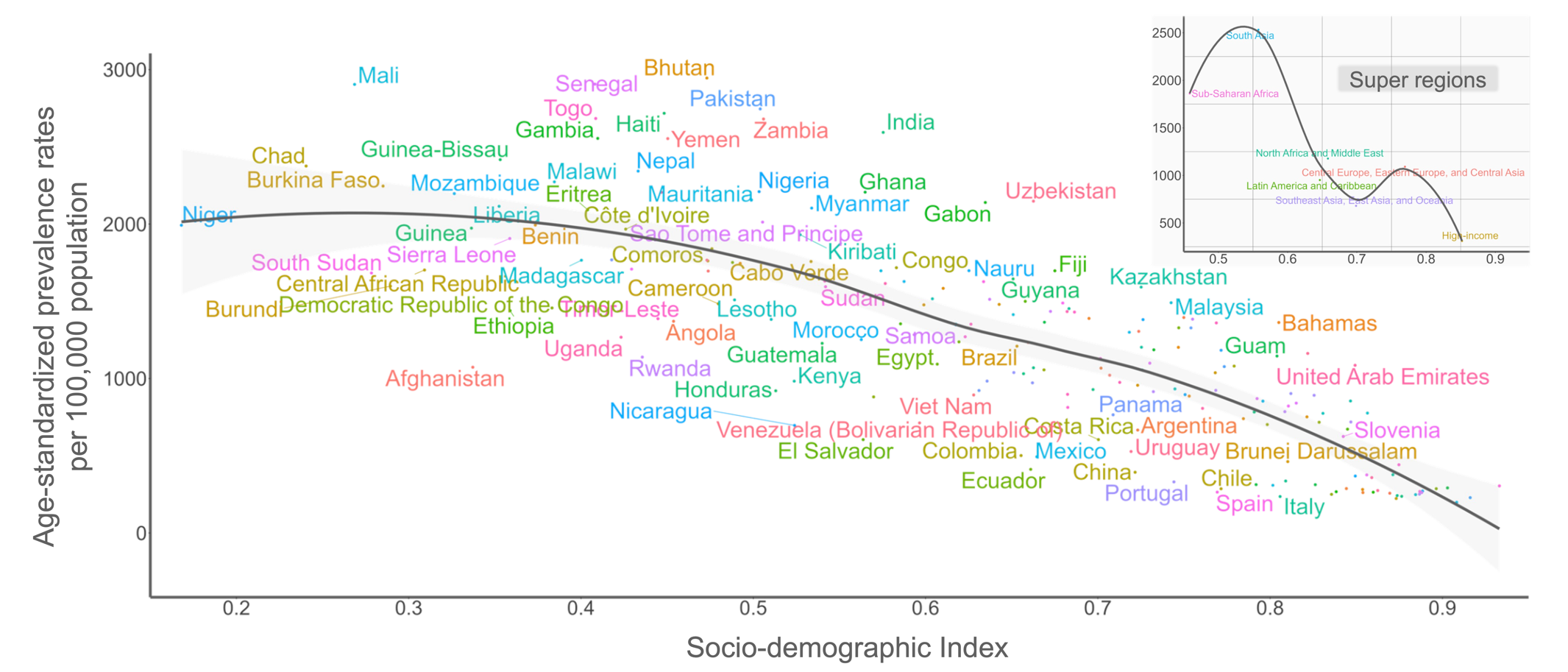


# Fig. S1 Association between age-standardized prevalence rates (per 100,000 population) of rIDPs and SDI values by countries and territories and GBD super regions in 2021. The top right panels show the classification of GBD super regions. The expected values based on age-standardized rates and SDI values by a smoothing spline model with Locally Weighted Scatterplot Smoothing method are shown in the black lines. Abbreviations: rIDP: rare infectious disease of poverty; SDI: socio-demographic index.

**
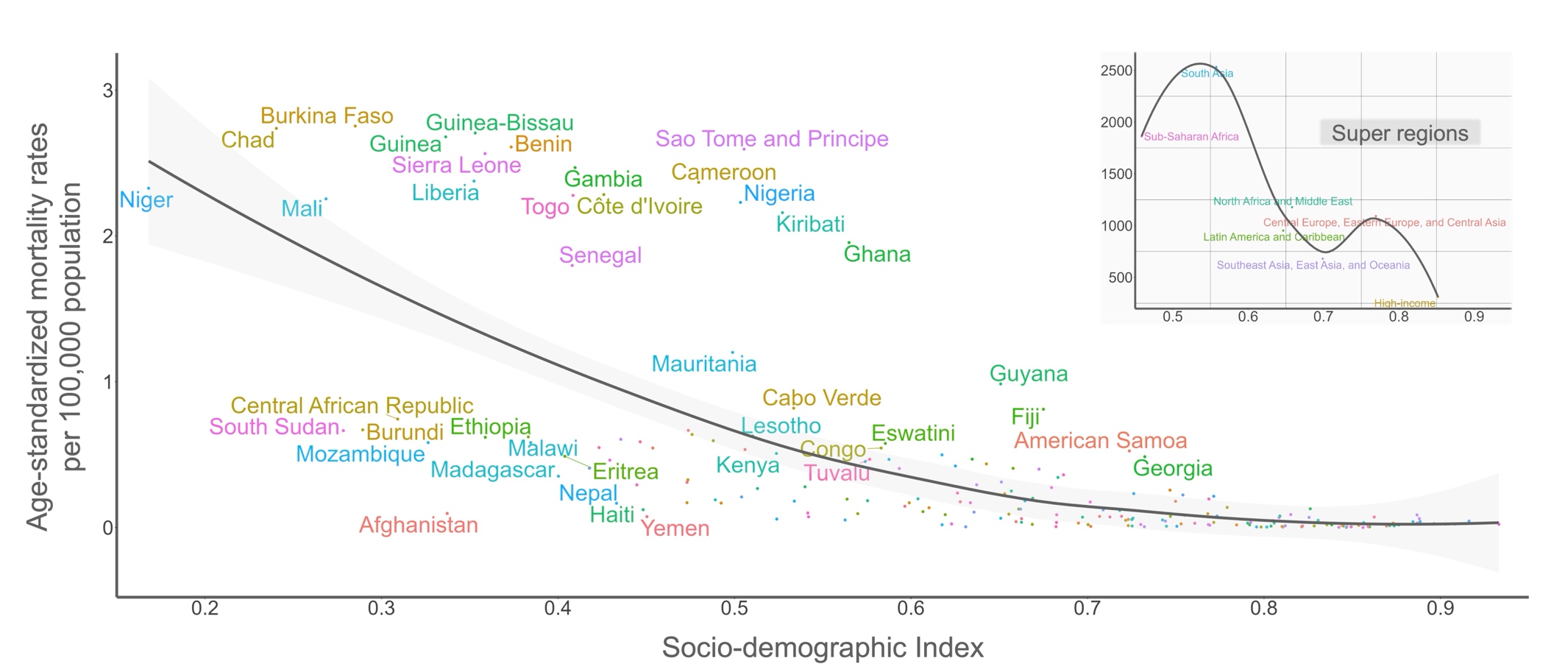
**

# Fig. S2 Association between age-standardized mortality rates (per 100,000 population) of rIDPs and SDI values by countries and territories and GBD super regions in 2021. The top right panels show the classification of GBD super regions. The expected values based on age-standardized rates and SDI values by a smoothing spline model with Locally Weighted Scatterplot Smoothing method are shown in the black lines. Abbreviations: rIDP: rare infectious disease of poverty; SDI: socio-demographic index.

**
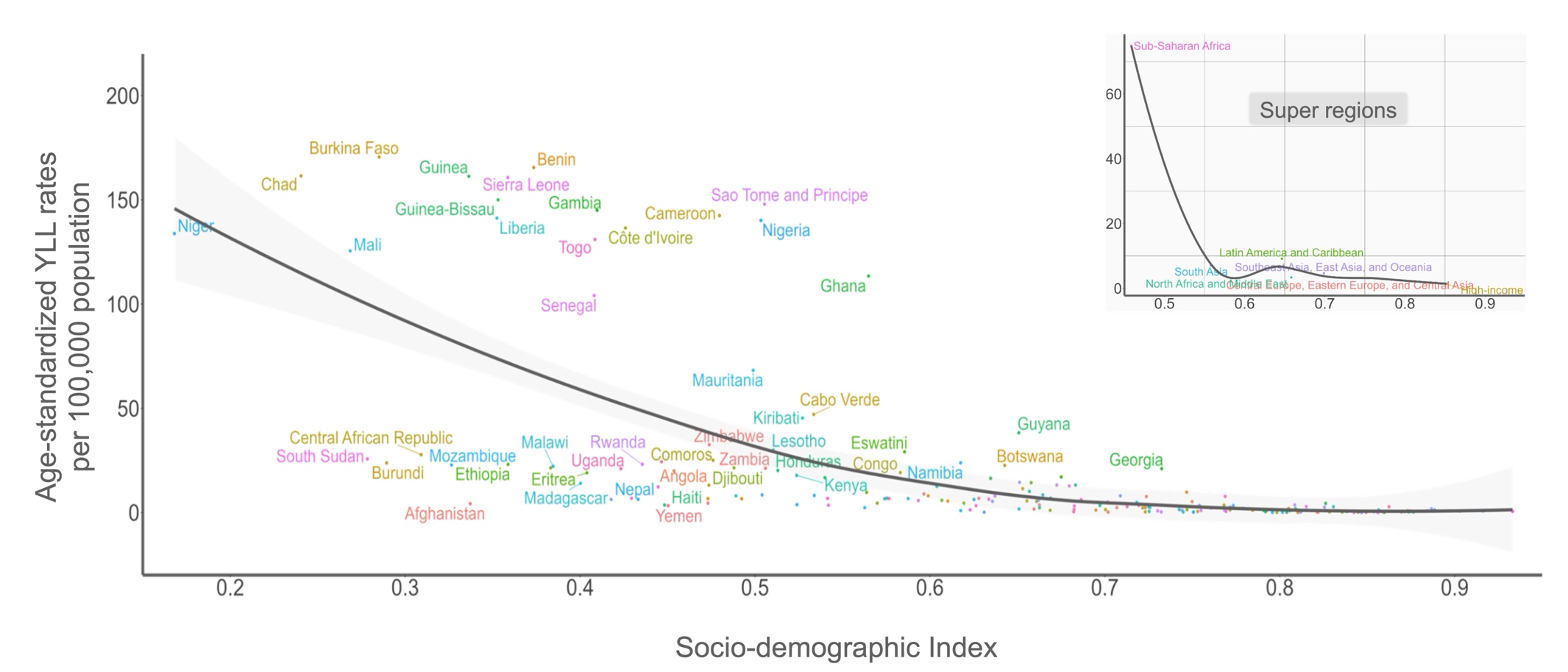
**

# Fig. S3 Association between age-standardized YLL rates (per 100,000 population) of rIDPs and SDI values by countries and territories and GBD super regions in 2021. The top right panels show the classification of GBD super regions. The expected values based on age-standardized rates and SDI values by a smoothing spline model with Locally Weighted Scatterplot Smoothing method are shown in the black lines. Abbreviations: rIDP: rare infectious disease of poverty; SDI: socio-demographic indexl; YLL: year of life lost.

**
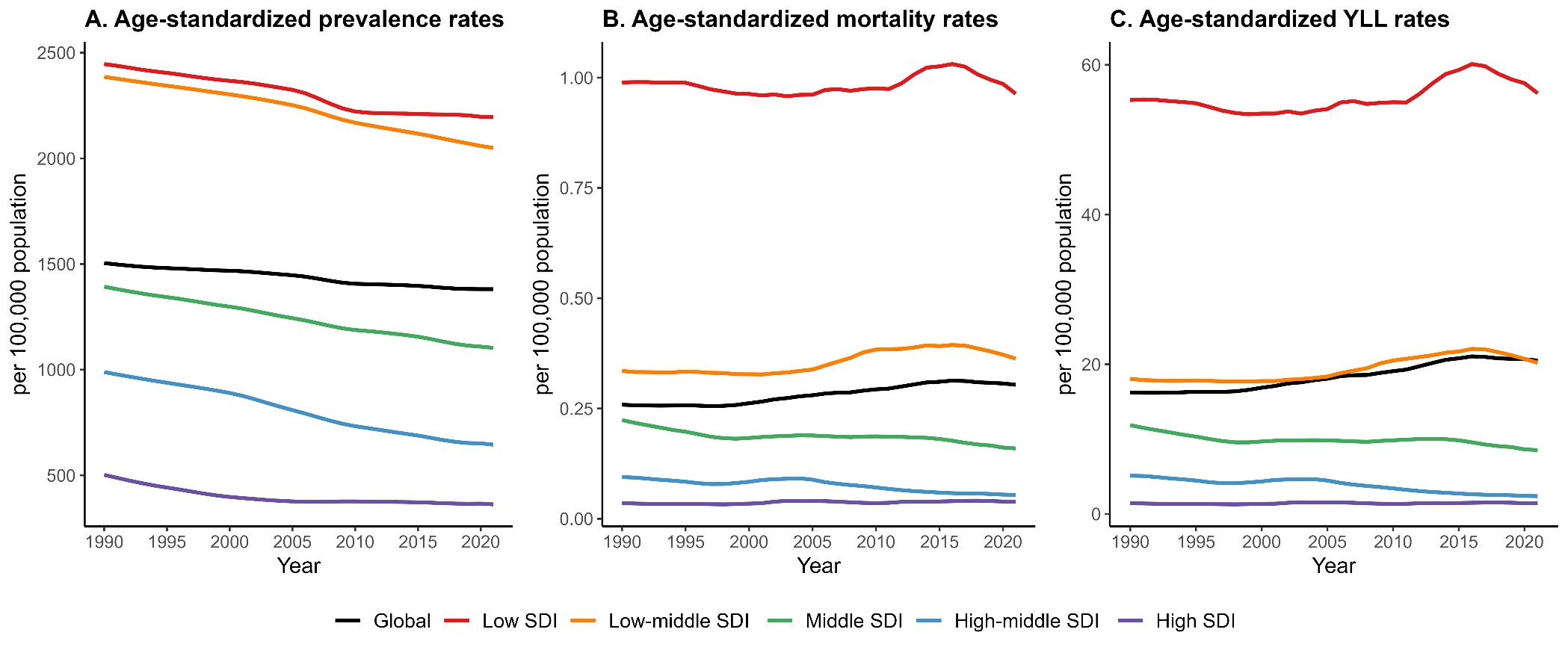
**

# Fig. S4 Temporal trends of age-standardized prevalence, mortality, and YLL rates of rIDPs by SDI levels from 1990 to 2021. Abbreviations: rIDP: rare infectious disease of poverty; SDI: socio-demographic index; YLL: year of life lost.


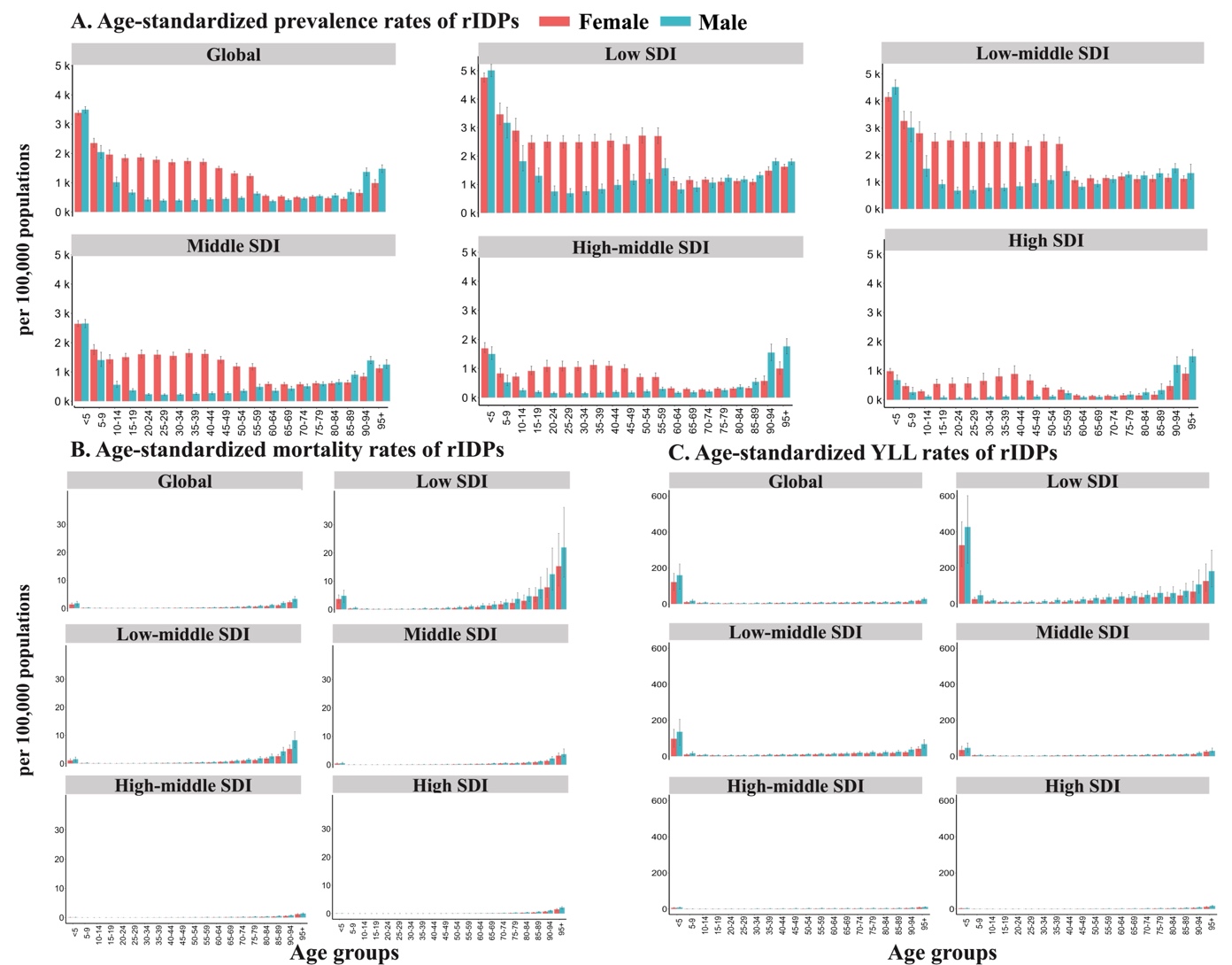


# Fig. S5 Age-standardized prevalence, mortality, and YLL rates (per 100,000 population) of rIDPs by age, sex, and SDI levels in 2021. Abbreviations: rIDP: rare infectious disease of poverty; SDI: socio-demographic index; YLL: year of life lost.
