# Supplementary material for: Global burden associated with rare infectious diseases of poverty in 2021: findings from the Global Burden of Disease Study 2021
Source: Infect Dis Poverty. 2024 Nov 13;13:85. doi: 10.1186/s40249-024-01249-6 (PMC11558835; doi:10.1186/s40249-024-01249-6)
Supplement: Supplementary file 2 — Additional file 2: Table. ISO-3 country code. [file 40249_2024_1249_MOESM2_ESM.docx]

Additional file 2

Table. ISO-3 country codes

| Countries and territories | ISO-3 |
| --- | --- |
| Afghanistan | AFG |
| Angola | AGO |
| Albania | ALB |
| Andorra | AND |
| United Arab Emirates | ARE |
| Argentina | ARG |
| Armenia | ARM |
| American Samoa | ASM |
| Antigua and Barbuda | ATG |
| Australia | AUS |
| Austria | AUT |
| Azerbaijan | AZE |
| Burundi | BDI |
| Belgium | BEL |
| Benin | BEN |
| Burkina Faso | BFA |
| Bangladesh | BGD |
| Bulgaria | BGR |
| Bahrain | BHR |
| Bahamas | BHS |
| Bosnia and Herzegovina | BIH |
| Belarus | BLR |
| Belize | BLZ |
| Bermuda | BMU |
| Bolivia | BOL |
| Brazil | BRA |
| Barbados | BRB |
| Brunei | BRN |
| Bhutan | BTN |
| Botswana | BWA |
| Central African Republic | CAF |
| Canada | CAN |
| Switzerland | CHE |
| Chile | CHL |
| China | CHN |
| Côte d'Ivoire | CIV |
| Cameroon | CMR |
| DR Congo | COD |
| Congo | COG |
| Cook Islands | COK |
| Colombia | COL |
| Comoros | COM |
| Cabo Verde | CPV |
| Costa Rica | CRI |
| Cuba | CUB |
| Cyprus | CYP |
| Czechia | CZE |
| Germany | DEU |
| Djibouti | DJI |
| Dominica | DMA |
| Denmark | DNK |
| Dominican Republic | DOM |
| Algeria | DZA |
| Ecuador | ECU |
| Egypt | EGY |
| Eritrea | ERI |
| Spain | ESP |
| Estonia | EST |
| Ethiopia | ETH |
| Finland | FIN |
| Fiji | FJI |
| France | FRA |
| Federated States of Micronesia | FSM |
| Gabon | GAB |
| United Kingdom | GBR |
| Georgia | GEO |
| Ghana | GHA |
| Guinea | GIN |
| Gambia | GMB |
| Guinea-Bissau | GNB |
| Equatorial Guinea | GNQ |
| Greece | GRC |
| Grenada | GRD |
| Greenland | GRL |
| Guatemala | GTM |
| Guam | GUM |
| Guyana | GUY |
| Honduras | HND |
| Croatia | HRV |
| Haiti | HTI |
| Hungary | HUN |
| Indonesia | IDN |
| India | IND |
| Ireland | IRL |
| Iran | IRN |
| Iraq | IRQ |
| Iceland | ISL |
| Israel | ISR |
| Italy | ITA |
| Jamaica | JAM |
| Jordan | JOR |
| Japan | JPN |
| Kazakhstan | KAZ |
| Kenya | KEN |
| Kyrgyzstan | KGZ |
| Cambodia | KHM |
| Kiribati | KIR |
| Saint Kitts and Nevis | KNA |
| South Korea | KOR |
| Kuwait | KWT |
| Laos | LAO |
| Lebanon | LBN |
| Liberia | LBR |
| Libya | LBY |
| Saint Lucia | LCA |
| Sri Lanka | LKA |
| Lesotho | LSO |
| Lithuania | LTU |
| Luxembourg | LUX |
| Latvia | LVA |
| Morocco | MAR |
| Monaco | MCO |
| Moldova | MDA |
| Madagascar | MDG |
| Maldives | MDV |
| Mexico | MEX |
| Marshall Islands | MHL |
| North Macedonia | MKD |
| Mali | MLI |
| Malta | MLT |
| Myanmar | MMR |
| Montenegro | MNE |
| Mongolia | MNG |
| Northern Mariana Islands | MNP |
| Mozambique | MOZ |
| Mauritania | MRT |
| Mauritius | MUS |
| Malawi | MWI |
| Malaysia | MYS |
| Namibia | NAM |
| Niger | NER |
| Nigeria | NGA |
| Nicaragua | NIC |
| Niue | NIU |
| Netherlands | NLD |
| Norway | NOR |
| Nepal | NPL |
| Nauru | NRU |
| New Zealand | NZL |
| Oman | OMN |
| Pakistan | PAK |
| Panama | PAN |
| Peru | PER |
| Philippines | PHL |
| Palau | PLW |
| Papua New Guinea | PNG |
| Poland | POL |
| Puerto Rico | PRI |
| North Korea | PRK |
| Portugal | PRT |
| Paraguay | PRY |
| Palestine | PSE |
| Qatar | QAT |
| Romania | ROU |
| Russia | RUS |
| Rwanda | RWA |
| Saudi Arabia | SAU |
| Sudan | SDN |
| Senegal | SEN |
| Singapore | SGP |
| Solomon Islands | SLB |
| Sierra Leone | SLE |
| El Salvador | SLV |
| San Marino | SMR |
| Somalia | SOM |
| Serbia | SRB |
| South Sudan | SSD |
| São Tomé and Príncipe | STP |
| Suriname | SUR |
| Slovakia | SVK |
| Slovenia | SVN |
| Sweden | SWE |
| Eswatini | SWZ |
| Seychelles | SYC |
| Syria | SYR |
| Chad | TCD |
| Togo | TGO |
| Thailand | THA |
| Tajikistan | TJK |
| Tokelau | TKL |
| Turkmenistan | TKM |
| Timor-Leste | TLS |
| Tonga | TON |
| Trinidad and Tobago | TTO |
| Tunisia | TUN |
| Türkiye | TUR |
| Tuvalu | TUV |
| Tanzania | TZA |
| Uganda | UGA |
| Ukraine | UKR |
| Uruguay | URY |
| United States of America | USA |
| Uzbekistan | UZB |
| Saint Vincent and the Grenadines | VCT |
| Venezuela | VEN |
| Virgin Islands | VIR |
| Viet Nam | VNM |
| Vanuatu | VUT |
| Samoa | WSM |
| Yemen | YEM |
| South Africa | ZAF |
| Zambia | ZMB |
| Zimbabwe | ZWE |
